# Metal‐Mediated Base Pairing of Rigid and Flexible Benzaldoxime Metallacycles

**DOI:** 10.1002/cbic.202000135

**Published:** 2020-05-05

**Authors:** Sajal Maity, Madhuri Hande, Tuomas Lönnberg

**Affiliations:** ^1^ Department of Chemistry University of Turku Vatselankatu 2 20014 Turku Finland

**Keywords:** base pairs, hybridization, mercury, palladium, oligonucleotides

## Abstract

Oligonucleotides incorporating a central C‐nucleoside with either a rigid or flexible benzaldoxime base moiety have been synthesized, and the hybridization properties of their metallacyclic derivatives have been studied by UV melting experiments. In all cases, the metallated duplexes were less stable than their unmetallated counterparts, and the metallacyclic nucleobases did not show a clear preference for any of the canonical nucleobases as a base‐pairing partner. With palladated oligonucleotides, increased flexibility translated to less severe destabilization, whereas the opposite was true for the mercurated oligonucleotides; this reflects the greater difficulties in accommodating a rigid Pd^II^‐mediated base pair than a rigid Hg^II^‐mediated base pair within the base stack of a double helix.

## Graphical abstract

**Take the strain**: A flexible Pd^II^‐mediated base pair of a benzaldoxime palladacycle disrupts oligonucleotide hybridization less than its rigid analogue, whereas the opposite is true for Hg^II^‐mediated base pairs. With a base pair of an incompatible geometry, increased flexibility alleviates some of the strain; with a strain‐free base pair it merely leads to an increased entropic penalty of hybridization.

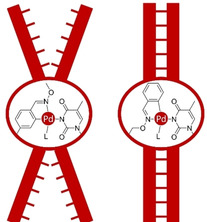

## Introduction

Double‐helical oligonucleotides incorporating metal‐mediated base pairs^1^ often exhibit higher thermal stability than their counterparts comprising solely of canonical hydrogen‐bonded base pairs, making metal‐mediated base pairing an attractive approach for conferring therapeutic oligonucleotides higher affinity for their target sequences.^2^ We have explored this possibility by studying the base pairing preferences of various organometallic nucleobase surrogates.^3^ Palladacyclic modifications have been of particular interest owing to the very high affinity of Pd^II^ for the canonical nucleobases^4^ and the wealth of pharmacological data already available on small molecular palladacyclic complexes.^5^ Recently, we were able to demonstrate the feasibility of cyclopalladated therapeutic oligonucleotides through splice‐correction in various human cell lines.^6^

Although a number of Pd^II^‐mediated base pairs have been reported since 1999,^7^, ^8^ stabilization of oligonucleotide duplexes by Pd^II^‐mediated base pairing remained elusive for a long time and even today the most compelling results have been obtained on duplexes having the Pd^II^‐mediated base pair(s) at a terminal position.^9^, ^10^ The difficulties in clearly demonstrating duplex stabilization by a central Pd^II^‐mediated base pair could stem from suboptimal geometries of the base pairs reported so far. Indeed, a thorough study of Pd^II^‐mediated base pairing between terpyridine and various azoles has revealed that a planar base pair was only formed with 1‐methyltetrazole, having no substituents (not even hydrogens) facing the Pd^II^‐terpyridine complex.^8^ The palladacyclic nucleobase surrogates leave the Pd^II^ center somewhat more exposed than the Pd^II^‐terpyridine complex but achieving a coplanar orientation of the palladacycle and the canonical nucleobase may still be difficult. As the steric requirements for base pairing are less strict at the termini than within the base stack, the stabilization provided by the high binding affinity of Pd^II^ is not offset by strain imposed by the unfavorable geometry of the Pd^II^‐mediated base pair.

**Figure 1:**
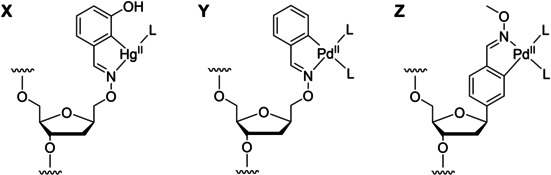
Structures of the organometallic nucleoside residues used in this study.

In this article, we present a comparison of the hybridization properties of oligonucleotides incorporating either a rigid or a flexible benzaldoxime palladacycle in the middle of their sequence (Figure 1). Both structures can place the Pd^II^ ion in the same position but the former (Figure 1Z) should be more favorably preorganized for base pairing with a complementary oligonucleotide. On the other hand, the latter (Figure 1Y) should be able to relieve at least some of the strain of accommodating a non‐planar base pair within the base stack of a double helix. Finally, an oligonucleotide incorporating a flexible covalently mercurated benzaldoxime (Figure 1X) was also included in the study. The linear coordination geometry of Hg^II^ complexes has been shown to be amenable to base pairing within a double helix and the resulting Hg^II^‐mediated base pairs are essentially strain‐free.^11^ Increased flexibility would, hence, not be expected to result in increased hybridization affinity in the case of Hg^II^‐mediated base pairs but the results will nonetheless serve as useful reference material for the interpretation of the results obtained on the palladacyclic structures.

## Results and Discussion

### Building block synthesis

**Scheme 1:**
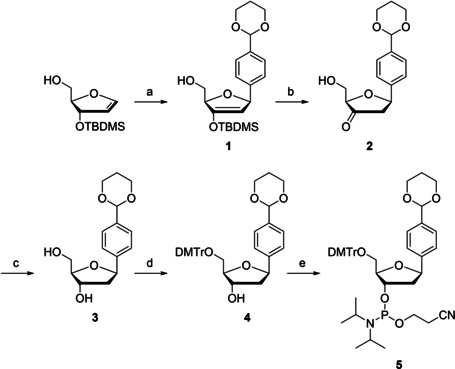
Synthesis of the protected benzaldehyde C‐nucleoside **3** and its phosphoramidite building block **5**. a) 2‐(4‐Bromophenyl)‐1,3‐dioxane, (*t*Bu_3_P)_2_Pd(0), DIPEA, Bu_4_NBr, 1,4‐dioxane, Ar atmosphere, 70 °C, 16 h; b) Et_3_N ⋅ 3 HF, THF, Ar atmosphere, 0 °C, 20 min; c) NaBH(OAc)_3_, MeCN, 0 °C, 2 h; d) DMTrCl, pyridine, 25 °C, 12 h; e) 2‐cyanoethyl‐*N*,*N*‐diisopropylchlorophosphoramidite, Et_3_N, CH_2_Cl_2_, N_2_ atmosphere, 25 °C, 2 h.

Synthesis of the protected benzaldehyde C‐nucleoside **3** and its phosphoramidite building block **5** is presented in Scheme 1. First, compound **1** was prepared by Heck coupling between {(2*R*,3*S*)‐3‐[(*tert*‐butyldimethylsilyl)oxy]‐2,3‐dihydrofuran‐2‐yl}methanol and 2‐(4‐bromophenyl)‐1,3‐dioxane. Compound **1** was then desilylated to the ketone intermediate **2** which, in turn, was reduced to the protected C‐nucleoside **3**. 5′‐Dimethoxytritylation of compound **3** afforded intermediate **4** and subsequent 3′‐phosphitylation the phosphoramidite building block **5**. Synthesis of compound **5** has been described previously^12^ but the pathway presented herein is significantly higher‐yielding.

**Scheme 2:**
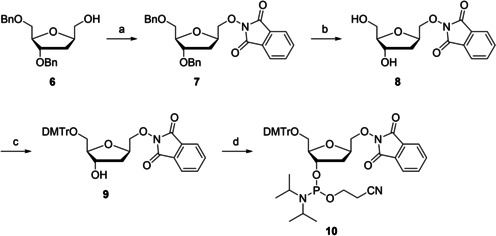
Synthesis of protected aminooxymethyl C‐nucleoside **8** and its phosphoramidite building block **10**. a) DIAD, HONPhth, Ph_3_P, THF, 25 °C, 12 h; b) Pd(OH)_2_/C, EtOAc, H_2_ atmosphere, 25 °C, 1 h; c) DMTrCl, pyridine, 25 °C, 12 h; d) 2‐cyanoethyl‐*N*,*N*‐diisopropylchlorophosphoramidite, Et_3_N, CH_2_Cl_2_, N_2_ atmosphere, 25 °C, 2 h.

Phthaloyl‐protected aminooxymethyl C‐nucleoside **8** and its phosphoramidite building block **10** were synthesized following the pathway outlined in Scheme 2. The fully protected aminooxymethyl C‐nucleoside **7** was first prepared by a Mitsunobu reaction between 3,5‐di‐*O*‐benzyl‐*C*‐hydroxymethyl‐2‐deoxy‐β‐d‐ribofuranose (**6**)^13^ and *N*‐hydroxhphthalimide. Removal of the benzyl protections by Pd(OH)_2_/C‐catalyzed hydrogenation afforded compound **8**. Preparation of compound **8** has been reported previously^14^ but the pathway presented herein is simpler and higher‐yielding. Finally, compound **8** was 5′‐dimethoxytritylated to give compound **9** which, in turn, was 3′‐phosphitylated to give the phosphoramidite building block **10**. These last two steps were carried out as described in the literature.^14^

### Oligonucleotide synthesis

Table 1 summarizes the sequences of the oligonucleotides used in the present study. Apart from the central variable residue, the sequences were identical to those used in our previous studies,^15^, ^16^ allowing a direct comparison of the melting temperatures. The modified oligonucleotides **ON1x**, **ON1y** and **ON1b** were assembled on an automated DNA/RNA synthesizer by the conventional phosphoramidite strategy, employing an extended coupling time for building blocks **5** and **10**. Normal (ca. 99 %) coupling yields were observed throughout the synthesis. The acetal and phthaloyl protections of the benzaldehyde and aminooxymethyl residues were removed on‐support by treatment with dichloroacetic acid in wet CH_2_Cl_2_ or hydrazine acetate in pyridine, respectively. The newly exposed aminooxy function was immediately allowed to react with either 3‐hydroxybenzaldehyde or benzaldehyde. Finally, oligonucleotides **ON1x**, **ON1y** and **ON1b** were released from the solid support and deprotected by conventional ammonolysis and purified by RP‐HPLC. **ON1x** eluted as two barely separable peaks, presumably attributable to *E* and *Z* isomers of the oxime bond. The stereoselectivity of oximation of benzaldehydes is known to be highly sensitive to the reaction conditions^17^ and, especially in the case of a potential hydrogen bond donor and acceptor, such as the hydroxy group (originally included to activate the aromatic ring towards *ortho*‐mercuration), the oligonucleotide environment probably affects as well. Unfortunately, the isolated amounts of the putative isomers of **ON1x** were not sufficient for more detailed characterization.

**Table 1:** Oligonucleotides used in this study.

Oligonucleotide	Sequence^[a]^
**ON1x**	5′‐CGAGCXCTGGC‐3′
**ON1x**−Hg	5′‐CGAGCX ^Hg^CTGGC‐3′
**ON1y**	5′‐CGAGCYCTGGC‐3′
**ON1y**−Pd	5′‐CGAGCY ^Pd^CTGGC‐3′
**ON1b**	5′‐CGAGCBCTGGC‐3′
**ON1z**	5′‐CGAGCZCTGGC‐3′
**ON1z**−Pd	5′‐CGAGCZ ^Pd^CTGGC‐3′
**ON2a**	5′‐GCCAGAGCTCG‐3′
**ON2c**	5′‐GCCAGCGCTCG‐3′
**ON2g**	5′‐GCCAGGGCTCG‐3′
**ON2t**	5′‐GCCAGTGCTCG‐3′

^[a]^ X refers to 3‐hydroxybenzylideneaminooxymethyl, X^Hg^ to (2‐mercuri‐3‐hydroxybenzylidene)aminooxymethyl, Y to benzylideneaminooxymethyl, Y^Pd^ to (2‐palladabenzylidene)aminooxymethyl, B to 4‐formylphenyl, Z to 4‐(methoxyiminomethyl)phenyl and Z^Pd^ to 3‐pallada‐4‐(methoxyiminomethyl)phenyl. In each sequence, the residue varied in the hybridization experiments has been underlined.

**ON1b** was treated with an aqueous solution of methoxylamine to convert the central benzaldehyde residue into an *O*‐methylbenzaldoxime residue. Oligonucleotide **ON1z** thus obtained was purified by RP‐HPLC and cyclopalladated in an aqueous solution of Li_2_PdCl_4_. The product mixture was fractioned on RP‐HPLC to afford the palladacyclic oligonucleotide **ON1z**−Pd. Finally, oligonucleotides **ON1b**, **ON1x**, **ON1y**, **ON1z** and **ON1z**−Pd were characterized by ESI‐TOF‐MS and quantified by UV spectrophotometry. Synthesis of the modified oligonucleotides **ON1x**−Hg and **ON1y**−Pd has been described previously.^14^

### Hybridization studies

Hybridization properties of the modified oligonucleotides **ON1x**, **ON1x**−Hg, **ON1y**, **ON1y**−Pd, **ON1z** and **ON1z**−Pd were studied by conventional UV melting experiments. Each modified oligonucleotide was mixed with each of the unmodified counterparts **ON2a**, **ON2c**, **ON2g** and **ON2t**, pairing the artificial residue with adenine, cytosine, guanine or thymine, respectively. The oligonucleotide concentrations of the samples were 1.0 μM, the pH 7.4 (20 mM cacodylate buffer) and the ionic strength 0.10 M (adjusted with sodium perchlorate). Melting profiles for unmodified duplexes of otherwise identical sequence but with cytosine in place of the modified residue have been reported previously.^16^

**Figure 2:**
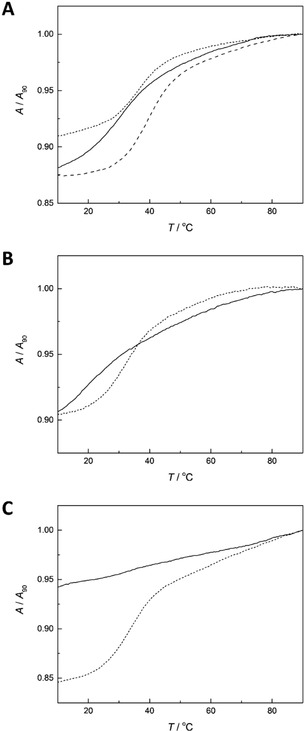
UV melting profiles for duplexes A) **ON1x** ⋅ **ON2t** (dotted and dashed line for faster and more slowly eluting isomer, respectively) and **ON1x**−Hg ⋅ **ON2t** (solid line), B) **ON1y** ⋅ **ON2t** (dotted line) and **ON1y**−Pd ⋅ **ON2t** (solid line) and C) **ON1z** ⋅ **ON2t** (dotted line) and **ON1z**−Pd ⋅ **ON2t** (solid line); pH 7.4 (20 mM cacodylate buffer); [oligonucleotides]=1.0 μM; *I*(NaClO_4_)=0.10 M.

Figure 2 depicts UV melting profiles of duplexes **ON1x** ⋅ **ON2t**, **ON1x**−Hg ⋅ **ON2t**, **ON1y** ⋅ **ON2t**, **ON1y**−Pd ⋅ **ON2t**, **ON1z** ⋅ **ON2t** and **ON1z**−Pd ⋅ **ON2t** as representative examples (all melting profiles can be found in the Supporting Information). With the notable exception of **ON1z**−Pd, monophasic sigmoidal melting curves were observed in most cases. Melting temperatures of the unmetallated duplexes formed by **ON1y**, **ON1z** and the faster‐eluting isomer of **ON1x** ranged from 33 to 36 °C, typical for 11‐mer oligodeoxynucleotides containing a single mismatch (Table 2). The more slowly eluting isomer of **ON1x**, in turn, exhibited a somewhat higher affinity for all of the unmodified oligonucleotides, the melting temperatures being approximately 40 °C. All of the metallated duplexes were less stable than their unmetallated counterparts. The destabilization was 3–5 °C for **ON1x**−Hg and 15–17 °C for **ON1y**−Pd, whereas with **ON1z**−Pd duplex formation was not observed at all over the temperature range used (10–90 °C). Apparently Pd^II^‐mediated base pairing by the benzaldoxime palladacycles of **ON1y**−Pd and **ON1z**−Pd causes strain that in the former case is to some extent relieved by the flexible structure of the palladacycle but in the latter case prevents formation of a double helix. Disruption of Watson‐Crick base pairing by a neighboring metal‐mediated base pairing has been described previously in detail with Ag(I) as the bridging metal ion.^18^ As an alternative explanation, destabilization of the metallated duplexes could be at least partly accounted for by competing intrastrand metal‐mediated base pairing, suggested previously for highly T‐rich sequences bearing organomercury nucleobases.^19^

**Table 2:** Melting temperatures of duplexes formed between the modified oligonucleotides **ON1x**, **ON1x**−Hg, **ON1y**, **ON1y**−Pd, **ON1z** and **ON1z**−Pd and the unmodified oligonucleotides **ON2a**, **ON2c**, **ON2g** and **ON2t**; pH 7.4 (20 mM cacodylate buffer); [oligonucleotides]=1.0 μM; *I*(NaClO_4_)=0.10 M.

	**ON2a**	**ON2c**	**ON2g**	**ON2t**
**ON1x**	36.4±0.7^[a]^	33.9±0.6^[a]^	36.3±0.7^[a]^	35.4±0.7^[a]^
	40.9±0.5^[b]^	39.6±0.4^[b]^	40.6±0.2^[b]^	39.4±0.6^[b]^
**ON1x**−Hg	30.9±0.8	31.0±0.9	31.6±0.6	32.3±0.8
**ON1y**	34.9±0.5	32.2±0.2	36.3±0.4	32.4±0.9
**ON1y**−Pd	18.0±0.7	15.9±0.9	n.a.^[c]^	17±1
**ON1z**	34.4±0.8	35.0±0.2	33.3±0.9	34.0±0.6
**ON1z**−Pd	n.a.^[c]^	n.a.^[c]^	n.a.^[c]^	n.a.^[c]^

[a] Faster‐eluting isomer. [b] Slower eluting isomer. [c] No sigmoidal melting curve was observed.

When applicable, thermodynamic parameters of hybridization were determined as described previously^20^ to gain further insight into the factors behind the different stabilities of the metallated and unmetallated duplexes. The enthalpies (Table 3) and entropies (Table 4) of hybridization of the unmetallated duplexes were similar to those previously reported for respective duplexes with a single mismatch in the middle of the sequence.^15^, ^21^ The origin of the different hybridization affinities of the two isomers of **ON1x** remained elusive as no consistent pattern of different enthalpies and entropies emerged. The relatively low stability of the duplexes formed by the mercurated oligonucleotide **ON1x**−Hg compared to respective duplexes incorporating a 5‐mercuricytosine^15^ or 3‐fluoro‐2‐mercuri‐6‐methylaniline^21^ residue in place of the (2‐mercuri‐3‐hydroxybenzylidene)aminooxymethyl residue, on the other hand, could be correlated with a relatively high entropy of hybridization. While this value was lower for **ON1x**−Hg (500–600 J mol^−1^ K^−1^) than for its unmercurated counterpart **ON1x** (700–800 J mol^−1^ K^−1^), it was still considerably higher than the respective value reported for those more stable mercurated duplexes (200–400 J mol^−1^ K^−1^). Hg^II^‐mediated base pairing generally leads to a reduced entropic penalty of hybridization owing to dehydration of the bridging Hg^II^ ion^15^, ^21^, ^22^ but with the flexible (2‐mercuri‐3‐hydroxybenzylidene)aminooxymethyl base of **ON1x**−Hg some of this effect is offset by restricted rotation of several σ bonds. The enthalpies of hybridization were similar for the duplexes formed by **ON1x**−Hg and those containing 5‐mercuricytosine, approximately 200 kJ mol^−1^.

**Table 3:** Enthalpies of hybridization for duplexes formed by the modified oligonucleotides **ON1x**, **ON1x**−Hg, **ON1y**, **ON1y**−Pd, **ON1z** and **ON1z**−Pd and the unmodified oligonucleotides **ON2a**, **ON2c**, **ON2g** and **ON2t**; pH 7.4 (20 mM cacodylate buffer); [oligonucleotides]=1.0 μM; *I*(NaClO_4_)=0.10 M.

	Δ*H*° [kJ mol^−1^]			
	**ON2a**	**ON2c**	**ON2g**	**ON2t**
**ON1x**	−279±3^[a]^	−259±2^[a]^	−252±2^[a]^	−285±4^[a]^
	−259±1^[b]^	−296±3^[b]^	−277±1^[b]^	−278±2^[b]^
**ON1x**−Hg	−219±2	−183±3	−234±4	−239±3
**ON1y**	−286±3	−278±3	−304±5	−320±6
**ON1y**−Pd	−166±2	−109±2	n.a.^[c]^	−177±4
**ON1z**	−336±5	−270±10	−232±3	−285±3
**ON1z**−Pd	n.a.^[c]^	n.a.^[c]^	n.a.^[c]^	n.a.^[c]^

^[a]^ Faster‐eluting isomer. ^[b]^ Slower eluting isomer. ^[c]^ No sigmoidal melting curve was observed.

**Table 4:** Entropies of hybridization for duplexes formed by the modified oligonucleotides **ON1x**, **ON1x**−Hg, **ON1y**, **ON1y**−Pd, **ON1z** and **ON1z**−Pd and the unmodified oligonucleotides **ON2a**, **ON2c**, **ON2g** and **ON2t**; pH 7.4 (20 mM cacodylate buffer); [oligonucleotides]=1.0 μM; *I*(NaClO_4_)=0.10 M.

	Δ*S*° [J mol^−1^ K^−1^]			
	**ON2a**	**ON2c**	**ON2g**	**ON2t**
**ON1x**	−774±9^[a]^	−725±6^[a]^	−693±6^[a]^	−800±10^[a]^
	−708±2^[b]^	−820±10^[b]^	−762±4^[b]^	−770±4^[b]^
**ON1x**−Hg	−591±6	−486±8	−640±10	−660±10
**ON1y**	−815±8	−794±8	−870±20	−930±20
**ON1y**−Pd	−445±5	−259±6	n.a.	−480±10
**ON1z**	−980±20	−770±30	−638±8	−808±7
**ON1z**−Pd	n.a.^[c]^	n.a.^[c]^	n.a.^[c]^	n.a.^[c]^

^[a]^ Faster‐eluting isomer. ^[b]^ Slower eluting isomer. ^[c]^ No sigmoidal melting curve was observed.

Compared to the well‐documented Hg^II^‐mediated base pairing, detailed thermodynamic data on Pd^II^‐mediated base pairing is scanty but reanalysis of UV melting profiles reported previously for octamer duplexes with an unpalladated or palladated benzylamine attached to the 5′ terminus through a flexible linker revealed more negative enthalpies and entropies of hybridization for the palladated oligonucleotides.^10^ Presumably Pd^II^‐mediated base pairing itself is an enthalpy‐driven process, whereas the entropic penalty is consistent with conformational restriction of the flexible linker. In the present case, however, both the enthalpies and the entropies of hybridization were less negative with **ON1y**−Pd than with **ON1y** and the differences were actually considerably larger than between **ON1x**−Hg and **ON1x**. This result, while in apparent conflict with the data obtained on oligonucleotides featuring terminal palladacyclic modifications, could be understood if formation of the Pd^II^‐mediated base prevented formation of some (in the case of **ON1y**−Pd) or all (in the case of **ON1z**−Pd) of the canonical Watson‐Crick base pairs.

All samples were also analyzed CD spectropolarimetrically for more detailed information on the secondary structures adopted by the oligonucleotides. The spectra were recorded between 10 and 90 °C at 10 °C intervals. At the low end of the temperature range, the spectra of the unmetallated duplexes were characteristic of B‐type double helices, with negative and positive Cotton effects of nearly equal intensity at 250 and 280 nm, respectively (spectra presented in the Supporting Information). The corresponding spectra of the duplexes formed by **ON1x**−Hg and **ON1y**−Pd were largely similar except for **ON1x**−Hg ⋅ **ON2g**, in which case the signal at 250 nm was hardly detectable. With **ON1z**−Pd the spectra were much more distorted and the ellipticity lower, in line with the inability of this oligonucleotide to form a double helix with any of the unmodified counterparts at temperatures above 10 °C. Gradual thermal diminution of the Cotton effects, consistent with denaturation of the double helix, was observed with all of the duplexes studied.

## Conclusion

Increased flexibility of the metallated nucleoside analog was found to have profoundly different effects on the melting temperatures of oligonucleotide duplexes containing a central Pd^II^‐ or Hg^II^‐mediated base pair. The oligonucleotide incorporating a flexible palladacyclic nucleoside formed more stable duplexes than its more rigid counterpart whereas comparison of the present data with those reported previously revealed the opposite to be true for oligonucleotides incorporating organomercury nucleosides. These results can be understood in terms of the different geometries of Pd^II^‐ and Hg^II^‐mediated base pairs. The former may be unable to assume the coplanar orientation preferred within the base stack of a double helix and in such a case a flexible structure might be able to alleviate some of the resulting strain. The latter, on the other hand, are usually nearly planar and geometrically very similar to canonical Watson‐Crick base pairs and in such a case increased flexibility will only result in an increased entropic penalty as the flexible structure is constrained within the base stack.

## Experimental Section

**General methods**: All experiments involving air and/or moisture sensitive compounds were performed using oven‐dried glassware under argon atmosphere. For preparation of HPLC elution buffers, freshly distilled triethylamine was used. Other commercially available chemicals were used without further purification unless otherwise stated. The solvents for organic synthesis were of reagent grade and dried over 4 Å molecular sieves. All reactions were monitored by thin layer chromatography (TLC), performed on Merck 60 (silica gel F254) plates. Chromatographic purification of products was accomplished using flash column chromatography on silica gel (230–400 mesh). ^1^H, ^13^C and ^31^P NMR spectra were recorded in deuterated solvents on a Bruker Biospin 500 or 600 MHz NMR spectrometer. Chemical shifts (δ, ppm) are quoted relative to the residual solvent peak as internal standard. Mass spectra were recorded on a Bruker Daltonics micrOTOF‐Q ESI mass spectrometer.

**Oligonucleotide synthesis**: Synthesis of the modified oligonucleotides **ON1x**−Hg and **ON1y**−Pd has been described previously. The other modified oligonucleotides (**ON1x**, **ON1y** and **ON1b**) were assembled on an Applied Biosystems 3400 automated DNA/RNA synthesizer by conventional phosphoramidite strategy. For the acetal protected benzaldehyde and the phtahaloyl‐protected aminooxymethyl C‐nucleoside phosphoramidite building blocks **2** and **8**, the coupling time was extended to 300 s. Based on trityl response, all couplings proceeded with normal efficiency. Removal of the acetal protecting group of the benzaldehyde residue was carried out on‐support by treatment with 2 % dichloroacetic acid and 1 % H_2_O in CH_2_Cl_2_ at 25 °C for 2 h.^23^ Removal of the phthaloyl protection of the aminooxy residue, in turn, was accomplished by on‐support treatment with hydrazine acetate in pyridine at 25 °C for 45 min.^24^ The support‐bound aminooxy‐functionalized oligonucleotide thus obtained was further treated with either 3‐hydroxybenzaldehyde (10 mg, 82 μmol) in CH_2_Cl_2_ (3.0 mL) or neat benzaldehyde (3.0 mL, 29 mmol) to eventually afford oligonucleotides **ON1x** and **ON1y**, respectively. Finally, oligonucleotides **ON1x**, **ON1y** and **ON1b** were released from the solid support and the phosphate and base moieties were deprotected by incubation in 25 % aqueous ammonia at 55 °C for 12 h and purified by RP‐HPLC on a Hypersil ODS C18 column (250×4.6 mm, 5 μm) eluting with a linear gradient of MeCN (5–30 % over 30 min) in 50 mM aqueous triethylammonium acetate. The flow rate was 1.0 mL min^−1^ and the detection wavelength 260 nm.

The central benzaldehyde residue of oligonucleotide **ON1b** was converted into an *O*‐methylbenzaldoxime residue by treatment with a mixture of methoxylamine hydrochloride (1.0 μmol) and NaOAc (1.0 μmol) in H_2_O (100 μL) at 25 °C for 12 h. The crude oligonucleotide **ON1z** thus obtained was purified by RP‐HPLC as described above. For cyclopalladation, **ON1z** (40 nmol), Li_2_PdCl_4_ (60 nmol) and NaOAc (240 nmol) were dissolved in H_2_O (10 μL) and the resulting mixture was incubated at 55 °C for 40 h. The crude product **ON1z**−Pd was purified by RP‐HPLC as described above. As reported previously for other cyclopalladated oligonucleotides,^16^ **ON1z**−Pd eluted as a broad and convoluted peak even after purification, presumably due to the relatively slow ligand‐exchange of Pd^II^ giving rise to a variety of slow‐equilibrating structures involving intra‐ as well as interstrand Pd^II^‐mediated base pairing. All of the newly synthesized oligonucleotides (**ON1b**, **ON1x**, **ON1y**, **ON1z** and **ON1z**−Pd) were characterized by ESI‐TOF‐MS and quantified UV spectrophotometrically using molar absorptivities calculated by an implementation of the nearest‐neighbors method.

**UV melting experiments**: UV melting profiles were acquired on a PerkinElmer Lambda 35 UV‐Vis spectrometer equipped with a Peltier temperature control unit, using quartz glass cuvettes with 10.00 mm optical path length. The samples were prepared by mixing appropriate oligonucleotides (1.0 μM) in 20 mM cacodylate buffer (pH 7.4), the ionic strength of which was adjusted to 0.10 M with NaClO_4_. Before each experiment, the samples were annealed by heating to 90 °C for 30 min and then allowing to gradually cool down to room temperature. Three denaturing and renaturing ramps were performed (10–90 °C, 0.5 °C min^−1^) with each sample and absorbance at *λ*=260 nm was recorded at 0.5 °C intervals. The melting temperatures were determined as inflection points on the UV melting curves.

**CD experiments**: CD spectra were recorded on an Applied Photophysics Chirascan spectropolarimeter equipped with a Peltier temperature control unit, using quartz glass cuvettes with 10.00 mm optical path length. Sample preparation was identical to the procedures used for the UV melting experiments above. Nine spectra were acquired for each sample at 10 °C intervals over *T*=10–90 °C and *λ*=200–400 nm. Before each measurement, the samples were allowed to equilibrate at the appropriate temperature for either 120 s (unmetallated duplexes) or 1800 s (metallated duplexes).

**2‐{4‐[3‐*O*‐(*tert*‐Butyldimethylsilyl)‐2‐deoxy‐2,3‐didehydro‐β‐d‐*erythro*‐pentofuranosyl]phenyl}‐1,3‐dioxane (1)**: 2‐(4‐Bromophenyl)‐1,3‐dioxane (158.6 mg, 0.652 mmol), {(2*R*,3*S*)‐3‐[(*tert*‐butyldimethylsilyl)oxy]‐2,3‐dihydrofuran‐2‐yl}methanol (150.0 mg, 0.652 mmol), Bu_4_NBr (631.0 mg, 1.96 mmol), DIPEA (340 μL, 1.96 mmol) and (^*t*^Bu_3_P)_2_Pd(0) (26.7 mg, 0.0520 mmol) were taken in a round‐bottomed flask and purged with argon. 1,4‐dioxane (3.0 mL, degassed) was added and the mixture stirred at 70 °C for 16 h. The volatiles were evaporated and the residue was co‐evaporated twice from toluene. The crude product was purified by silica gel flash column chromatography (hexane/EtOAc 3 : 1, *v*/*v*) affording compound **1** (210.0 mg, 82 %) as a colorless foam. ^1^H NMR (400 MHz, CDCl_3_): *δ*=7.48 (d, *J*=8.0 Hz, 2H, H3 & H5), 7.39 (d, *J*=8.0 Hz, 2H, H2 & H6), 5.74 (d, *J*=3.6 Hz, 1H, H1′), 5.50 (s, 1H, dioxane−H1), 4.83 (s, 1H, H2′), 4.62 (m, 1H, H4′), 4.25 (dd, *J*=11.2, 5.2 Hz, 2H, dioxane−H3 & H5), 3.97 (td, *J*=12.6, 2.0 Hz, 2H, dioxane−H3 & H5), 3.74–3.66 (m, 2H, H5′ & H5′′), 2.22 (m, 1H, dioxane−H4), 1.95 (s, 1H, 5′‐OH), 1.43 (d, *J*=13.2, 1H, dioxane−H4), 0.96 (s, 9H, SiC(CH_3_)), 0.24 (s, 3H, SiCH_3_), 0.21 (s, 3H, SiCH_3_). ^13^C NMR (100 MHz, CDCl_3_): *δ*=151.5 (C3′), 143.0 (C1), 138.8 (C4), 127.1 (C3 & C5), 126.3 (C2 & C6), 101.4 (dioxane−C1), 101.3 (C2′), 84.6 (C4′), 83.3 (C1′), 67.3 (dioxane−C3 & C5), 63.0 (C5′), 25.8 (dioxane−C5), 25.6 (SiC(CH_3_)_3_), 18.1 (SiC(CH_3_)_3_), −4.9 (SiCH_3_), −5.0 (SiCH_3_). HRMS (ESI^+^): *m*/*z*: calcd for C_21_H_32_O_5_SiNa [*M*+Na]^+^: 415.1911; found: 415.1894.

**2‐{4‐[(2*R*,5*R*)‐5‐(Hydroxymethyl)‐4‐oxotetrahydrofuran‐2‐yl]phenyl}‐1,3‐dioxane (2)**: Et_3_N ⋅ 3 HF (126.0 μL, 0.712 mmol) was added to a stirred solution of compound **1** (202.0 mg, 0.515 mmol) in dry THF (2.0 mL) under argon at 0 °C. After 20 min, the reaction mixture was passed through a short silica gel bed which was then washed with EtOAc. The filtrate was evaporated and the residue purified by silica gel flash column chromatography (hexane/EtOAc 1 : 2, *v*/*v*) affording compound **2** (129.1 mg, 90 %) as a white solid. ^1^H NMR (400 MHz, CDCl_3_): *δ*=7.53 (d, *J*=8.0 Hz, 2H, H3 & H5), 7.43 (d, *J*=8.0 Hz, 2H, H2 & H6), 5.52 (s, 1H, dioxane−H1), 5.22 (dd, *J*=11.8, 5.6 Hz, H1′), 4.28 (dd, *J*=11.6, 4.8 Hz, 2H, dioxane−H3 & H5), 4.04–3.93 (m, 5H, dioxane−H3 & H5, H5′ & H5′′, H4′), 2.86 (dd, *J*=18.4, 6.0 Hz, 1H, H2′), 2.48 (dd, *J*=18.4, 11.2 Hz, 1H, H2′′), 2.21 (m, 1H, dioxane−H4), 1.46 (dd, *J*=8.0, 0.8 Hz, 1H, dioxane−H4). ^13^C NMR (100 MHz, CDCl_3_): *δ*=213.7 (C3′), 140.3 (C1), 139.1 (C4), 126.5 (C3 & C5), 126.0 (C2 & C6), 101.2 (dioxane−C1), 82.3 (C4′), 77.4 (C1′), 67.4 (dioxane−C3 & C5), 61.6 (C5′), 45.4 (C2′), 25.7 (dioxane−C4). HRMS (ESI^+^): *m*/*z*: calcd for C_15_H_18_O_5_Na [*M*+Na]^+^: 301.1046; found: 301.1045.

**2‐[4‐(2‐Deoxy‐β‐d‐*erythro*‐pentofuranosyl)phenyl]‐1,3‐dioxane (3)**: NaBH(OAc)_3_ (674.1 mg, 3.14 mmol) was added to a stirred solution of the ketone **2** (294.0 mg, 1.06 mmol) in dry MeCN (7.0 mL) at 0 °C. Stirring was continued at 0 °C for 2 h, after which the reaction was quenched by addition of MeOH, and the volatiles were evaporated. The residue was purified by silica gel flash column chromatography (MeOH/CH_2_Cl_2_ 1 : 9, *v*/*v*) affording compound **3** (281.4 mg, 95 %) as a yellow oil. ^1^H NMR (400 MHz, MeOD): *δ*=7.43 (d, *J*=8.0 Hz, 2H, H3 & H5), 7.39 (d, *J*=8.4 Hz, 2H, H2 & H6), 5.51 (s, 1H, dioxane−H1), 5.13 (dd, *J*=10.4, 5.6 Hz, 1H, H1′), 4.31 (d, *J*=5.6 Hz, 1H, H3′), 4.19 (dd, *J*=11.6, 5.2 Hz, 2H, dioxane−H3 & H5), 4.02–3.94 (m, 3H, dioxane−H3 & H5, H4′), 3.67 (m, 2H, H5′ & H5′′), 2.21–2.07 (m, 2H, H2′, dioxane−H4), 1.91 (m, 1H, H2′′), 1.44 (d, *J*=13.6 Hz, 1H, dioxane−H4). ^13^C NMR (100 MHz, MeOD): *δ*=142.5 (C1), 138.3 (C4), 125.9 (C3 & C5), 125.5 (C2 & C6), 101.3 (dioxane−C1), 87.8 (C4′), 79.9 (C1′), 73.0 (C3′), 67.0 (dioxane−C3 & C5), 62.7 (C5′), 43.6 (C2′), 25.6 (dioxane−C4). HRMS (ESI^+^): *m*/*z*: calcd for C_15_H_20_O_5_Na [*M*+Na]^+^: 303.1203; found: 303.1202.

**2‐{4‐[5‐*O*‐(4,4′‐Dimethoxytrityl)‐2‐deoxy‐β‐d‐*erythro*‐pentofuranosyl]phenyl}‐1,3‐dioxane (4)**: Compound **3** (227.0 mg, 0.810 mmol) was coevaporated twice from dry pyridine (20 mL), and the residue was dissolved in dry pyridine (10 mL). DMTrCl (302.0 mg, 0.891 mmol) was added and the resulting mixture stirred at 25 °C for 12 h, after which it was concentrated under reduced pressure. The residue was dissolved in CH_2_Cl_2_ (100 mL), washed with saturated aq. NaHCO_3_ (100 mL), dried over anhydrous Na_2_SO_4_, and evaporated to dryness. The residue was purified by silica gel flash chromatography (Et_3_N/hexane/EtOAc 2 : 19 : 19, *v*/*v*/*v*) affording compound **4** (425.1 mg, 90 %) as a white foam. ^1^H NMR (500 MHz, CDCl_3_): *δ*=7.52–7.47 (m, 4H, H3 & H5, Ph−H2 & H6), 7.41–7.38 (m, 6H, H2 & H6, MeOPh−H2 & H6), 7.31 (t, *J*=7.5 Hz, 2H, Ph−H3 & H5), 7.24 (t, *J*=7.5 Hz, 1H, Ph−H4), 6.87–6.84 (m, 4H, MeOPh−H3 & H5), 5.52 (s, 1H, dioxane−H1), 5.18 (dd, *J*=10.0, 6.0 Hz, 1H, H1′), 4.38 (m, 1H, H3′), 4.30–4.27 (m, 2H, dioxane−H3 & H5), 4.08 (td, *J*=6.0, 2.0 Hz, 1H, H4′), 4.01 (td, *J*=12.0, 3.0 Hz, 2H, dioxane−H3 & H5), 3.81 (s, 6H, OCH_3_), 3.37 (dd, *J*=9.5, 4.5 Hz, 1H, H5′), 3.30 (dd, *J*=9.5, 4.5 Hz, 1H, H5′′), 2.23 (m, 1H, dioxane−H4), 2.16 (ddd, *J*=13.0, 5.5, 2.0 Hz, 1H, H2′), 1.96 (m, 1H, H2′′), 1.47 (m, 1H, dioxane−H4). ^13^C NMR (125 MHz, CDCl_3_): *δ*=158.5 (MeOPh−C4), 144.9 (Ph−C1), 142.7 (C1), 138.0 (C4), 136.1 (MeOPh−C1), 130.14 (MeOPh−C2 & C6), 130.12 (MeOPh−C2 & C6), 128.3 (Ph−C3 & C5), 127.9 (Ph−C2 & C6), 126.8 (Ph−C4), 126.1 (C3 & C5), 125.8 (C2 & C6), 113.2 (MeOPh−C3 & C5), 101.5 (dioxane−C1), 86.4 (C4′), 86.2 (CAr_3_), 79.8 (C1′), 74.5 (C3′), 67.4 (dioxane−C3 & C5), 64.5 (C5′), 55.2 (OCH_3_), 44.1 (C2′), 25.8 (dioxane−C4). HRMS (ESI^+^): *m*/*z*: calcd for C_36_H_38_O_7_Na [*M*+Na]^+^: 605.2510; found: 605.2511.

**2‐(4‐{3‐*O*‐[(2‐Cyanoethoxy)(*N***,***N*** **‐diisopropylamino)phosphinyl]‐5‐*O*‐(4,4′‐dimethoxytrityl)‐2‐deoxy‐β‐d‐*erythro*‐pentofuranosyl}phenyl)‐1,3‐dioxane (5)**: Et_3_N (467.0 μL, 3.35 mmol) and 2‐cyanoethyl‐*N*,*N*‐diisopropylchlorophosphoramidite (149.0 μL, 0.669 mmol) were added to a stirred solution of compound **4** (325.0 mg, 0.558 mmol) in dry CH_2_Cl_2_ (3.0 mL) at 25 °C under N_2_. The reaction mixture was stirred for 2 h, after which it was diluted with CH_2_Cl_2_ (50 mL) and washed with saturated aq. NaHCO_3_ (50 mL). The organic layer was dried over Na_2_SO_4_ and evaporated to dryness. The residue was purified by silica gel flash column chromatography (Et_3_N/EtOAc/hexane 1 : 19 : 30, *v*/*v*/*v*) affording compound **5** (388.0 mg, 89 % combined yield of two diastereomers) as a white foam. ^1^H NMR (500 MHz, CDCl_3_, faster‐eluting diastereomer): *δ*=7.52–7.43 (m, 6H, Ph−H2 & H6, H2 & H6, H3 & H5), 7.41–7.37 (m, 4H, MeOPh−H2 & H6), 7.32–7.29 (m, 2H, Ph−H3 & H5), 7.24 (m, 1H, Ph−H4), 6.86–6.83 (m, 4H, MeOPh−H3 & H5), 5.52 (s, 1H, dioxane−H1), 5.19 (dd, *J*=10.5, 5.0 Hz, 1H, H1′), 4.52 (m, 1H, H3′), 4.30–4.26 (m, 3H, dioxane−H3 & H5, H4′), 4.01 (m, 2H, dioxane−H3 & H5), 3.82 (s, 6H, OCH_3_), 3.74–3.69 (m, 2H, OCH_2_CH_2_CN), 3.67–3.61 (m, 2H, NCHMe_2_), 3.36 (dd, *J*=10.0, 4.0 Hz, 1H, H5′), 3.28 (dd, *J*=10.0, 4.5 Hz, 1H, H5′′), 2.47 (m, 2H, CH_2_CN), 2.33 (dd, *J*=13.0, 5.5 Hz, 1H, H2′), 2.26 (m, 1H, dioxane−H4), 2.01 (m, 1H, H2′′), 1.47 (m, 1H, dioxane−H4), 1.21 (d, *J*=7.0 Hz, 6H, NCH(CH_3_)_2_), 1.18 (d, *J*=7.0 Hz, 6H, NCH(CH_3_)_2_). ^13^C NMR (125 MHz, CDCl_3_, faster‐eluting diastereomer): *δ*=158.5 (MeOPh−C4), 144.9 (Ph−C1), 142.5 (C1), 138.0 (C4), 136.2 (MeOPh−C1), 136.1 (MeOPh−C1), 130.18 (MeOPh−C2 & C6), 130.15 (MeOPh−C2 & C6), 128.3 (Ph−C3 & C5), 127.8 (Ph−C2 & C6), 126.7 (Ph−C4), 126.0 (C3 & C5), 125.9 (C2 & C6), 117.5 (CN), 113.1 (MeOPh−C3 & C5), 101.5 (dioxane−C1), 86.1 (CAr_3_), 86.0 (d, *J*=3.8 Hz, C4′), 80.1 (C1′), 75.7 (d, *J*=16.5 Hz, C3′), 67.4 (dioxane−C3 & C5), 64.1 (C5′), 58.3 (d, *J*=18.8 Hz, OCH_2_CH_2_CN), 55.2 (OCH_3_), 43.5 (d, *J*=4.5 Hz, C2′), 43.2 (d, J=12.4 Hz, NCHMe_2_), 25.8 (dioxane−C4), 24.6 (d, *J*=7.3 Hz, NCH(CH_3_)_2_), 24.5 (d, *J*=7.1 Hz, NCH(CH_3_)_2_), 20.2 (d, *J*=7.0 Hz, CH_2_CN). ^31^P (162 MHz, CDCl_3_, faster‐eluting diastereomer): *δ*=148.1. ^1^H NMR (500 MHz, CDCl_3_, more slowly eluting diastereomer): *δ*=7.51–7.43 (m, 6H, Ph−H2 & H6, H2 & H6, H3 & H5), 7.40–7.36 (m, 4H, MeOPh−H2 & H6), 7.30–7.27 (m, 2H, Ph−H3 & H5), 7.22 (m, 1H, Ph−H4), 6.85–6.81 (m, 4H, MeOPh−H3 & H5), 5.52 (s, 1H, dioxane−H1), 5.19 (dd, *J*=10.5, 5.0 Hz, 1H, H1′), 4.52 (m, 1H, H3′), 4.29 (m, 2H, dioxane−H3 & H5), 4.24 (m, 1H, H4′), 4.01 (m, 2H, dioxane−H3 & H5), 3.87–3.77 (m, 2H, OCH_2_CH_2_CN), 3.80 (s, 6H, OCH_3_), 3.62–3.53 (m, 2H, NCHMe_2_), 3.31 (dd, *J*=10.0, 4.5 Hz, H5′), 3.26 (dd, *J*=10.0, 4.5 Hz, 1H, H5′′), 2.63 (t, *J*=6.5 Hz, 2H, CH_2_CN), 2.40 (dd, *J*=13.0, 5.0 Hz, 1H, H2′), 2.25 (m, 1H, dioxane−H4), 1.99 (m, 1H, H2′′), 1.47 (m, 1H, dioxane−H4), 1.19 (d, *J*=6.5 Hz, 6H, NCH(CH_3_)_2_), 1.10 (d, *J*=7.0 Hz, 6H, NCH(CH_3_)_2_). ^13^C NMR (125 MHz, CDCl_3_, more slowly eluting diastereomer): *δ*=158.4 (MeOPh−C4), 144.9 (Ph−C1), 142.5 (C1), 138.0 (C4), 136.13 (MeOPh−C1), 136.11 (MeOPh−C1), 130.17 (MeOPh−C2 & C6), 130.13 (MeOPh−C2 & C6), 128.3 (Ph−C3 & C5), 127.8 (Ph−C2 & C6), 126.7 (Ph−C4), 126.0 (C3 & C5), 125.8 (C2 & C6), 117.5 (CN), 113.1 (MeOPh−C3 & C5), 101.5 (dioxane−C1), 86.0 (CAr_3_), 85.7 (d, *J*=5.8 Hz, C4′), 80.0 (C1′), 76.1 (d, *J*=17.1 Hz, C3′), 67.4 (dioxane−C3 & C5), 64.2 (C5′), 58.4 (d, *J*=18.9 Hz, OCH_2_CH_2_CN), 55.2 (OCH_3_), 43.5 (d, J=4.2 Hz, C2′), 43.2 (d, *J*=12.8 Hz, NCHMe_2_), 25.8 (dioxane−C4), 24.6 (d, *J*=7.4 Hz, NCH(CH_3_)_2_), 24.5 (d, *J*=7.2 Hz, NCH(CH_3_)_2_), 20.4 (d, *J*=7.2 Hz, CH_2_CN). ^31^P (162 MHz, CDCl_3_, more slowly eluting diastereomer): *δ*=147.8. HRMS (ESI^+^): *m*/*z*: calcd for C_45_H_55_N_2_O_8_PK [*M*+K]^+^: 821.3328; found: 821.3325.

**3,5‐Di‐*O*‐benzyl‐*C*‐phthalimidooxymethyl‐2‐deoxy‐β‐d‐ribofuranose (7)**: A solution of DIAD (1.51 mL, 7.64 mmol) in dry THF (8.0 mL) was added dropwise to a stirred solution of compound **6^[^** ^13]^ (1.93 g, 5.88 mmol), *N*‐hydroxyphthalimide (1.25 g, 7.64 mmol) and PPh_3_ (2.0 g, 7.64 mmol) in dry THF (65.0 mL) at 0 °C. The resulting mixture was stirred at 0 °C for 2 h and then at 25 °C for 12 h, after which it was evaporated to dryness. The residue was suspended in H_2_O and extracted with EtOAc (2×50 mL). The combined extracts were dried over Na_2_SO_4_ and evaporated to dryness. The residue was purified by silica gel flash column chromatography (hexane/EtOAc 7 : 3, *v*/*v*) affording compound **7** (2.39 g, 86 %) as a colorless syrup. ^1^H NMR (500 MHz, CDCl_3_): *δ*=7.83 (m, 2H, phthaloyl), 7.74 (m, 2H, phthaloyl), 7.37–7.27 (m, 10H, phenyl), 4.58 (m, 1H, H1′), 4.55 (d, *J*=4.5 Hz, 2H, PhCH_2_), 4.52 (brs, 2H, PhCH_2_), 4.35–4.29 (m, 2H, CH_2_ON), 4.19 (m, 1H, H4′), 4.11 (m, 1H, H3′), 3.54 (dd, *J*=10.0, 5.0 Hz, 1H, H5′), 3.47 (dd, *J*=10.0, 5.0 Hz, 1H, H5′′), 2.21 (ddd, *J*=13.0, 6.0, 2.0 Hz, 1H, H2′), 2.04 (ddd, *J*=13.0, 10.0, 6.5 Hz, 1H, H2′′). ^13^C NMR (125 MHz, CDCl_3_): *δ*=163.3 (C=O), 138.2 (phenyl−C1), 138.1 (phenyl−C1), 134.4 (phthaloyl−C3 & C4), 129.0 (phthaloyl−C1 & C6), 128.43 (phenyl−C3 & C5), 128.37 (phenyl−C3 & C5), 127.70 (phenyl−C4), 127.68 (phenyl−C4), 127.60 (phenyl−C2 & C6), 127.57 (phenyl−C2 & C6), 123.5 (phthaloyl−C2 & C5), 84.1 (C4′), 80.6 (C3′), 79.6 (CH_2_ON), 76.4 (C1′), 73.4 (PhCH_2_), 71.2 (PhCH_2_), 70.9 (C5′), 34.6 (C2′). HRMS (ESI^+^): *m*/*z*: calcd for C_28_H_27_O_6_NNa [*M*+Na]^+^: 496.1731; found: 496.1728.

***C*** **‐phthalimidooxymethyl‐2‐deoxy‐β‐d‐ribofuranose (8)**: Compound **7** (622.0 mg, 1.315 mmol) was dissolved in EtOAc (50.0 mL). 20 % Pd(OH)_2_/C (190.0 mg) was added, the reaction vessel was filled with H_2_, and the reaction mixture was stirred at 25 °C under H_2_ atmosphere (balloon pressure) for 1 h. The mixture was filtered through celite, and the filter was washed with EtOAc (2×20 mL). The combined filtrates were concentrated under vacuum and the residue was purified by silica gel flash column chromatography (MeOH/CH_2_Cl_2_ 1 : 9, *v*/*v*) affording compound **8** (231.0 mg, 60 %) as colorless syrup. ^1^H NMR (500 MHz, MeOD) *δ*=7.87–7.83 (m, 4H, phthaloyl), 4.51 (m, 1H, H1′), 4.30–4.24 (m, 3H, CH_2_ON, H3′), 3.82 (m, 1H, H4′), 3.57–3.51 (m, 2H, H5′ & H5′′), 2.09–1.97 (m, 2H, H2′ & H2′′). *δ*=163.5 (C=O), 134.4 (phthaloyl−C3 & C4), 128.9 (phthaloyl−C1 & C6), 122.9 (phthaloyl−C2 & C5), 87.7 (C4′), 79.5 (CH_2_ON), 76.1 (C1′), 72.1 (C3′), 62.4 (C5′), 36.6 (C2′). *m*/*z*: calcd for C_14_H_15_O_6_NNa [*M*+Na]^+^: 316.0792; found: 316.0792.

**5‐*O*‐(4,4′‐Dimethoxytrityl)‐*C*‐phthalimidooxymethyl‐2‐deoxy‐β‐d‐ribofuranose (9)**: Compound **9** was prepared from compound **8** as described in the literature^14^ and its NMR and mass spectra were identical to those reported previously.

**3‐*O*‐[(2‐Cyanoethoxy)(*N***,***N*** **‐diisopropylamino)phosphinyl]‐5‐*O*‐(4,4′‐dimethoxytrityl)‐*C*‐phthalimidooxymethyl‐2‐deoxy‐β‐d‐ribofuranose (10)**: Compound **10** was prepared from compound **9** as described in the literature^14^ and its NMR and mass spectra were identical to those reported previously.

## Conflict of interest

The authors declare no conflict of interest.

## Supporting information

As a service to our authors and readers, this journal provides supporting information supplied by the authors. Such materials are peer reviewed and may be re‐organized for online delivery, but are not copy‐edited or typeset. Technical support issues arising from supporting information (other than missing files) should be addressed to the authors.

Supplementary
