# Supplementary material for: Metal‐Mediated Base Pairing of Rigid and Flexible Benzaldoxime Metallacycles
Source: Chembiochem. 2020 May 5;21(16):2321–8. doi: 10.1002/cbic.202000135 (PMC7496235; doi:10.1002/cbic.202000135)
Supplement: Supplementary file 1 — Supplementary [file CBIC-21-2321-s001.pdf]

ChemBioChem

Supporting Information

# Metal-Mediated Base Pairing of Rigid and Flexible Benzaldoxime Metallacycles

Sajal Maity, Madhuri Hande, and Tuomas Lönnberg*© 2020 The Authors. Published by Wiley-VCH Verlag GmbH & Co. KGaA. This is an open access article under the terms of the Creative Commons Attribution License, which permits use, distribution and reproduction in any medium, provided the original work is properly cited.

**Contents**


| | |
|---|---|
| $^{1}H$ NMR spectrum of compound **1** | S3 |
| $^{13}C$ NMR spectrum of compound **1** | S4 |
| $^{1}H$ NMR spectrum of compound **2** | S5 |
| $^{13}C$ NMR spectrum of compound **2** | S6 |
| $^{1}H$ NMR spectrum of compound **3** | S6 |
| $^{13}C$ NMR spectrum of compound **3** | S7 |
| $^{1}H$ NMR spectrum of compound **4** | S8 |
| $^{13}C$ NMR spectrum of compound **4** | S11 |
| $^{1}H$ NMR spectrum of compound **5** (faster eluting diastereomer) | S12 |
| $^{13}C$ NMR spectrum of compound **5** (faster eluting diastereomer) | S17 |
| $^{31}P$ NMR spectrum of compound **5** (faster eluting diastereomer) | S19 |
| $^{1}H$ NMR spectrum of compound **5** (slower eluting diastereomer) | S20 |
| $^{13}C$ NMR spectrum of compound **5** (slower eluting diastereomer) | S24 |
| $^{31}P$ NMR spectrum of compound **5** (slower eluting diastereomer) | S27 |
| $^{1}H$ NMR spectrum of compound **7** | S27 |
| $^{13}C$ NMR spectrum of compound **7** | S30 |
| $^{1}H$ NMR spectrum of compound **8** | S31 |
| $^{13}C$ NMR spectrum of compound **8** | S33 |
| **Figure S1.** HPLC traces of crude and purified oligonucleotide **ON1x**. | S34 |
| **Figure S2.** Mass spectrum of the faster-eluting isomer of oligonucleotide **ON1x**. | S35 |
| **Figure S3.** Mass spectrum of the faster-eluting isomer of oligonucleotide **ON1x**. | S36 |
| **Figure S4.** HPLC traces of crude and purified oligonucleotide **ON1y**. | S37 |
| **Figure S5.** Mass spectrum of oligonucleotide **ON1y**. | S38 |
| **Figure S6.** HPLC traces of crude and purified oligonucleotide **ON1b**. | S39 |
| **Figure S7.** Mass spectrum of oligonucleotide **ON1b**. | S40 |
| **Figure S8.** HPLC traces of crude and purified oligonucleotide **ON1z**. | S41 |
| **Figure S9.** Mass spectrum of oligonucleotide **ON1z**. | S42 |
| **Figure S10.** HPLC traces of crude and purified oligonucleotide **ON1z-Pd**. | S43 |
| **Figure S11.** Mass spectrum of oligonucleotide **ON1z-Pd**. | S44 |
| **Figure S12.** UV melting profiles for duplexes **ON1x•ON2a** and **ON1x-Hg•ON2a**. | S45 |
| **Figure S13.** UV melting profiles for duplexes **ON1x•ON2c** and **ON1x-Hg•ON2c**. | S45 |
| **Figure S14.** UV melting profiles for duplexes **ON1x•ON2g** and **ON1x-Hg•ON2g**. | S46 |
| **Figure S15.** UV melting profiles for duplexes **ON1x•ON2t** and **ON1x-Hg•ON2t**. | S46 |
| **Figure S16.** UV melting profiles for duplexes **ON1y•ON2a** and **ON1y-Pd•ON2a**. | S47 |
| **Figure S17.** UV melting profiles for duplexes **ON1y•ON2c** and **ON1y-Pd•ON2c**. | S47 |
| **Figure S18.** UV melting profiles for duplexes **ON1y•ON2g** and **ON1y-Pd•ON2g**. | S48 |
| **Figure S19.** UV melting profiles for duplexes **ON1y•ON2t** and **ON1y-Pd•ON2t**. | S48 |
| **Figure S20.** UV melting profiles for duplexes **ON1z•ON2a** and **ON1z-Pd•ON2a**. | S49 |
| **Figure S21.** UV melting profiles for duplexes **ON1z•ON2c** and **ON1z-Pd•ON2c**. | S49 |
| **Figure S22.** UV melting profiles for duplexes **ON1z•ON2g** and **ON1z-Pd•ON2g**. | S50 |
| **Figure S23.** UV melting profiles for duplexes **ON1z•ON2t** and **ON1z-Pd•ON2t**. | S50 |
| **Figure S24.** CD spectra of duplex **ON1x•ON2a** (faster eluting isomer of **ON1x**). | S51 |
| **Figure S25.** CD spectra of duplex **ON1x•ON2c** (faster eluting isomer of **ON1x**). | S51 |
| **Figure S26.** CD spectra of duplex **ON1x•ON2g** (faster eluting isomer of **ON1x**). | S52 |
| **Figure S27.** CD spectra of duplex **ON1x•ON2t** (faster eluting isomer of **ON1x**). | S52 |

**Contents (continued)**


| | |
|---|---|
| **Figure S28.** CD spectra of duplex **ON1x•ON2a** (slower eluting isomer of **ON1x**). | S53 |
| **Figure S29.** CD spectra of duplex **ON1x•ON2c** (slower eluting isomer of **ON1x**). | S53 |
| **Figure S30.** CD spectra of duplex **ON1x•ON2g** (slower eluting isomer of **ON1x**). | S54 |
| **Figure S31.** CD spectra of duplex **ON1x•ON2t** (slower eluting isomer of **ON1x**). | S54 |
| **Figure S32.** CD spectra of duplex **ON1x-Hg•ON2a**. | S55 |
| **Figure S33.** CD spectra of duplex **ON1x-Hg•ON2c**. | S55 |
| **Figure S34.** CD spectra of duplex **ON1x-Hg•ON2g**. | S56 |
| **Figure S35.** CD spectra of duplex **ON1x-Hg•ON2t**. | S56 |
| **Figure S36.** CD spectra of duplex **ON1y•ON2a**. | S57 |
| **Figure S37.** CD spectra of duplex **ON1y•ON2c**. | S57 |
| **Figure S38.** CD spectra of duplex **ON1y•ON2g**. | S58 |
| **Figure S39.** CD spectra of duplex **ON1y•ON2t**. | S58 |
| **Figure S40.** CD spectra of duplex **ON1y-Pd•ON2a**. | S59 |
| **Figure S41.** CD spectra of duplex **ON1y-Pd•ON2c**. | S59 |
| **Figure S42.** CD spectra of duplex **ON1y-Pd•ON2g**. | S60 |
| **Figure S43.** CD spectra of duplex **ON1y-Pd•ON2t**. | S60 |
| **Figure S44.** CD spectra of duplex **ON1z•ON2a**. | S61 |
| **Figure S45.** CD spectra of duplex **ON1z•ON2c**. | S61 |
| **Figure S46.** CD spectra of duplex **ON1z•ON2g**. | S62 |
| **Figure S47.** CD spectra of duplex **ON1z•ON2t**. | S62 |
| **Figure S48.** CD spectra of duplex **ON1z-Pd•ON2a**. | S63 |
| **Figure S49.** CD spectra of duplex **ON1z-Pd•ON2c**. | S63 |
| **Figure S50.** CD spectra of duplex **ON1z-Pd•ON2g**. | S64 |
| **Figure S51.** CD spectra of duplex **ON1z-Pd•ON2t**. | S64 |

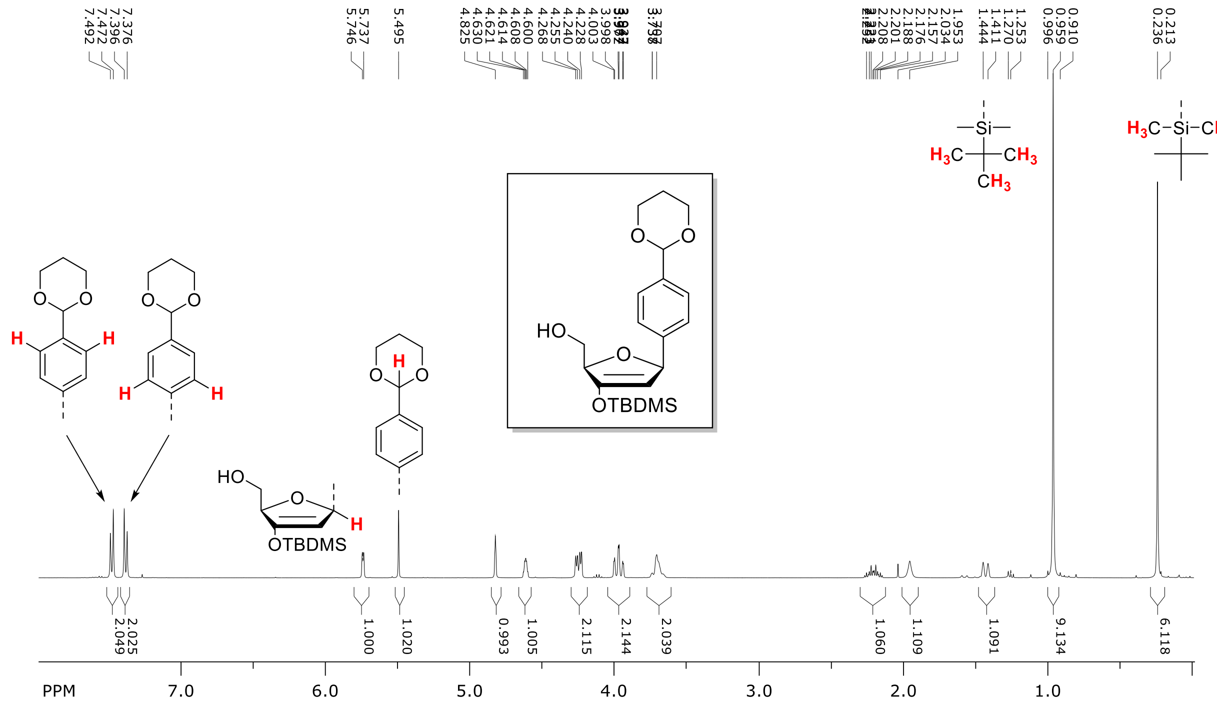

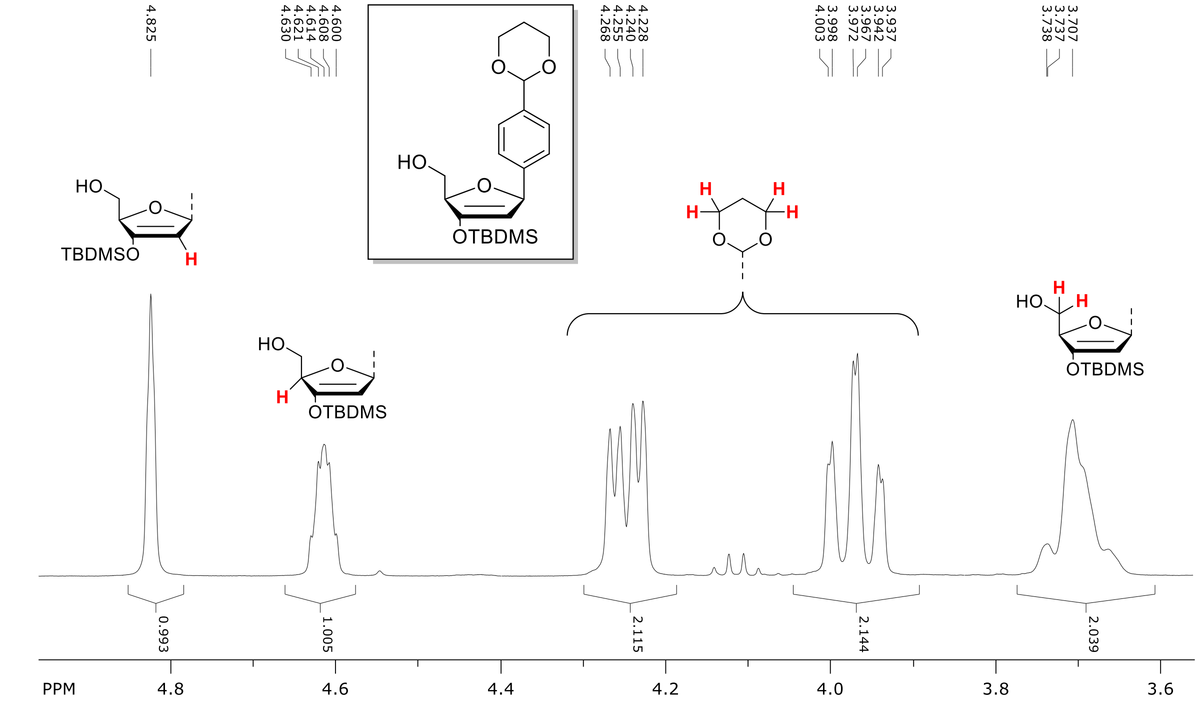

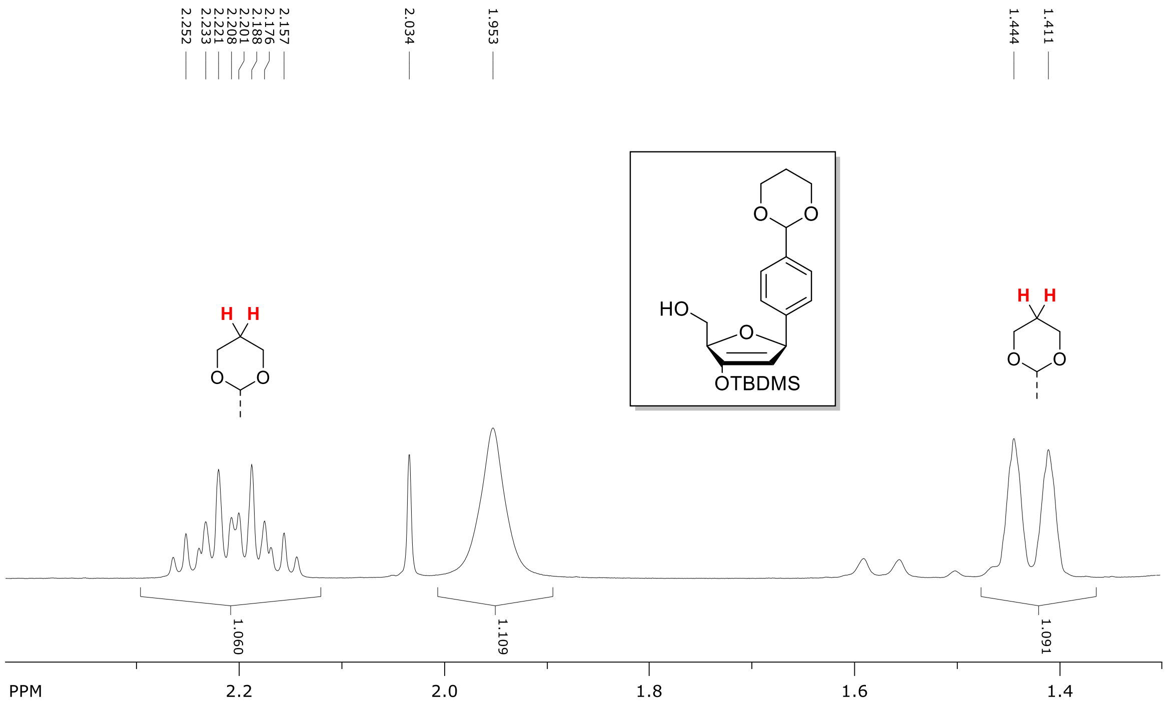

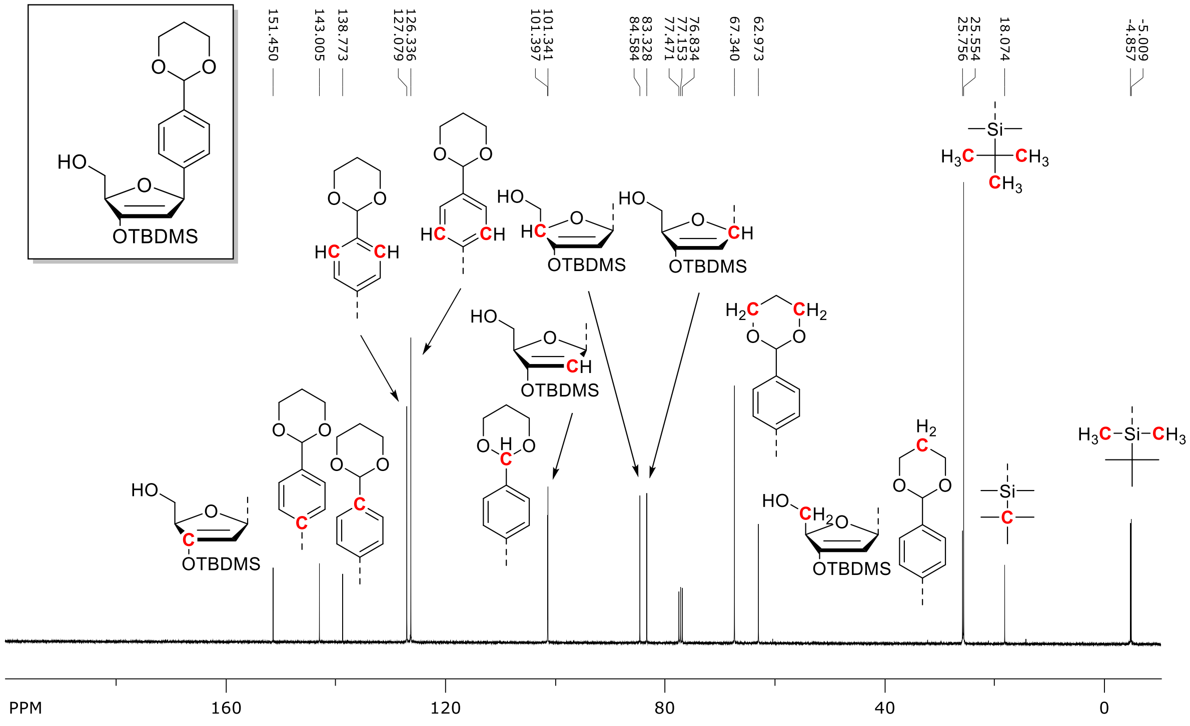

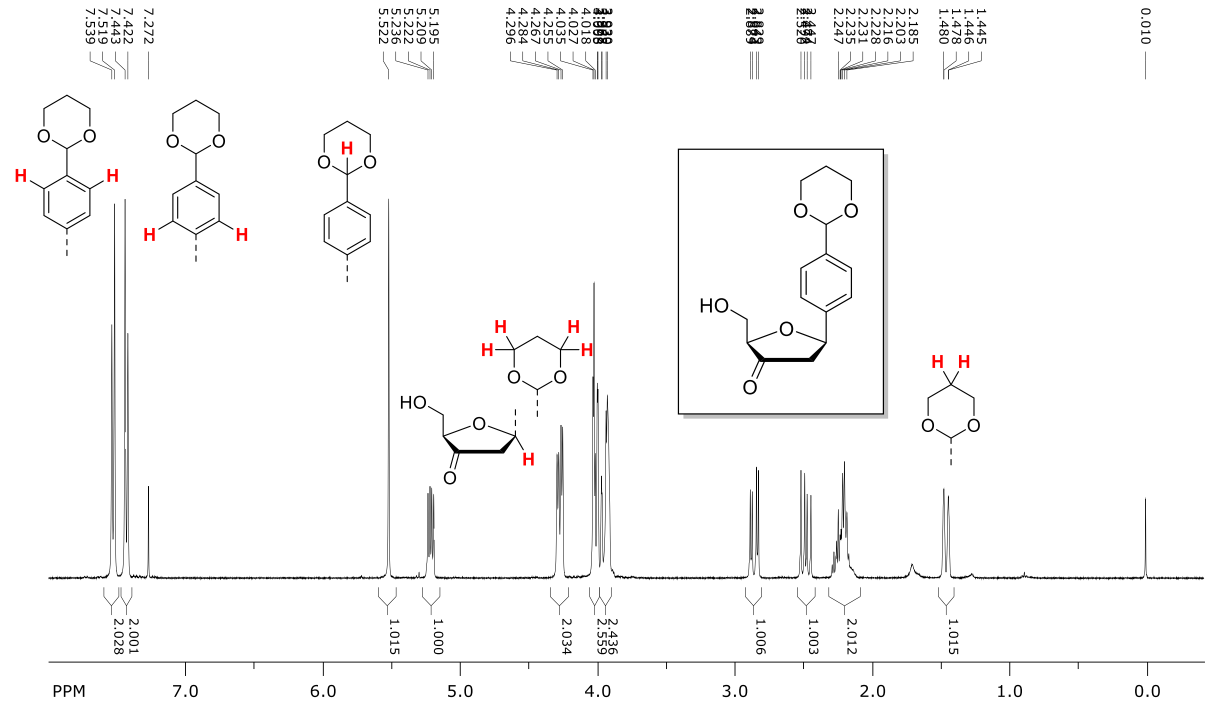
7.539 7.519 7.443 7.422 7.272
5.522 5.236 5.222 5.209 5.195
4.296 4.284 4.267 4.255 4.035 4.027 4.018
2.247 2.235 2.231 2.228 2.216 2.203 2.185
1.480 1.478 1.446 1.445
0.010
HO
O
H
2.028 2.001 1.015 1.000 2.034 2.559 2.436 1.006 1.003 2.012 1.015
PPM 7.0 6.0 5.0 4.0 3.0 2.0 1.0 0.0

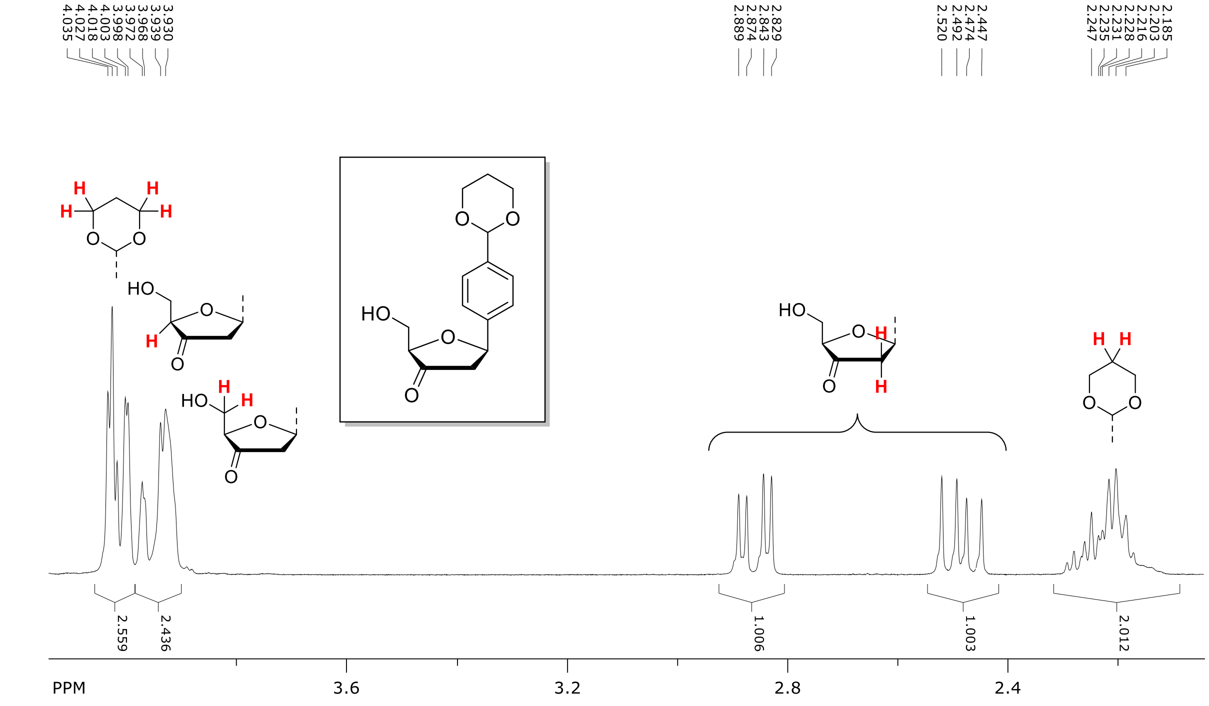
4.035 4.027 4.018 4.003 3.998 3.972 3.968 3.939 3.930
2.889 2.874 2.843 2.829
2.520 2.492 2.474 2.447
2.247 2.235 2.231 2.228 2.216 2.203 2.185
HO
O
H
2.559 2.436 1.006 1.003 2.012
PPM 3.6 3.2 2.8 2.4

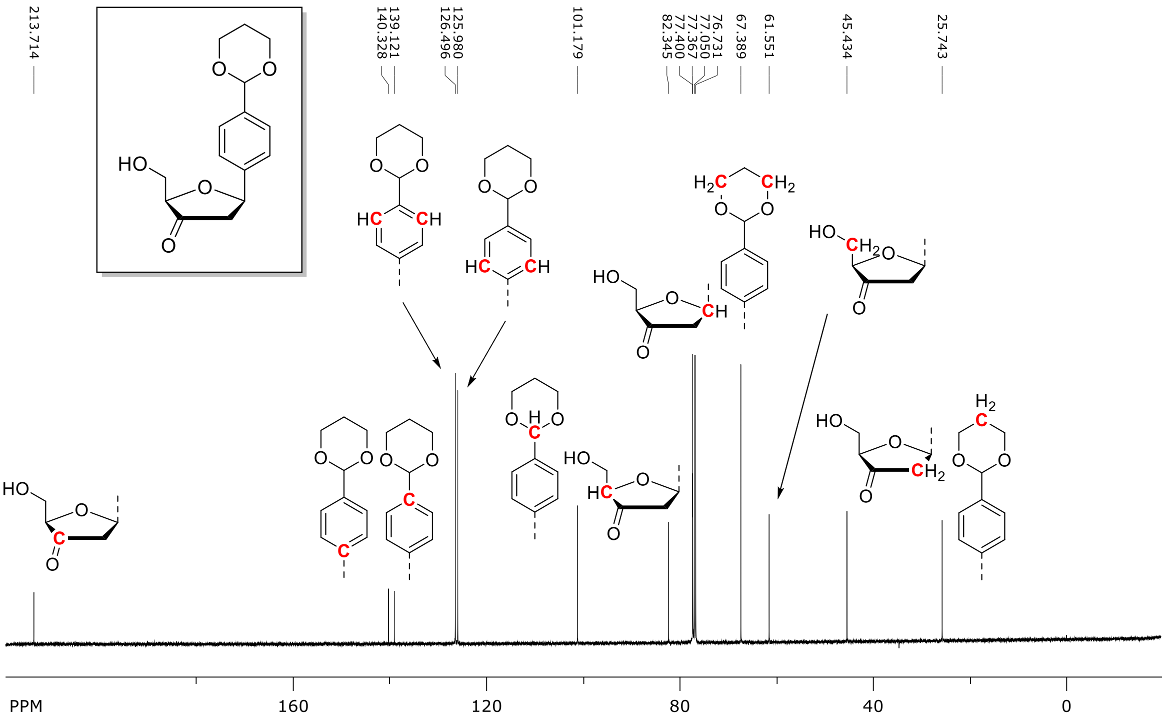
213.714
140.328
139.121
126.496
125.980
101.179
82.345
77.400
77.367
77.050
76.731
67.389
61.551
45.434
25.743
PPM
160
120
80
40
0

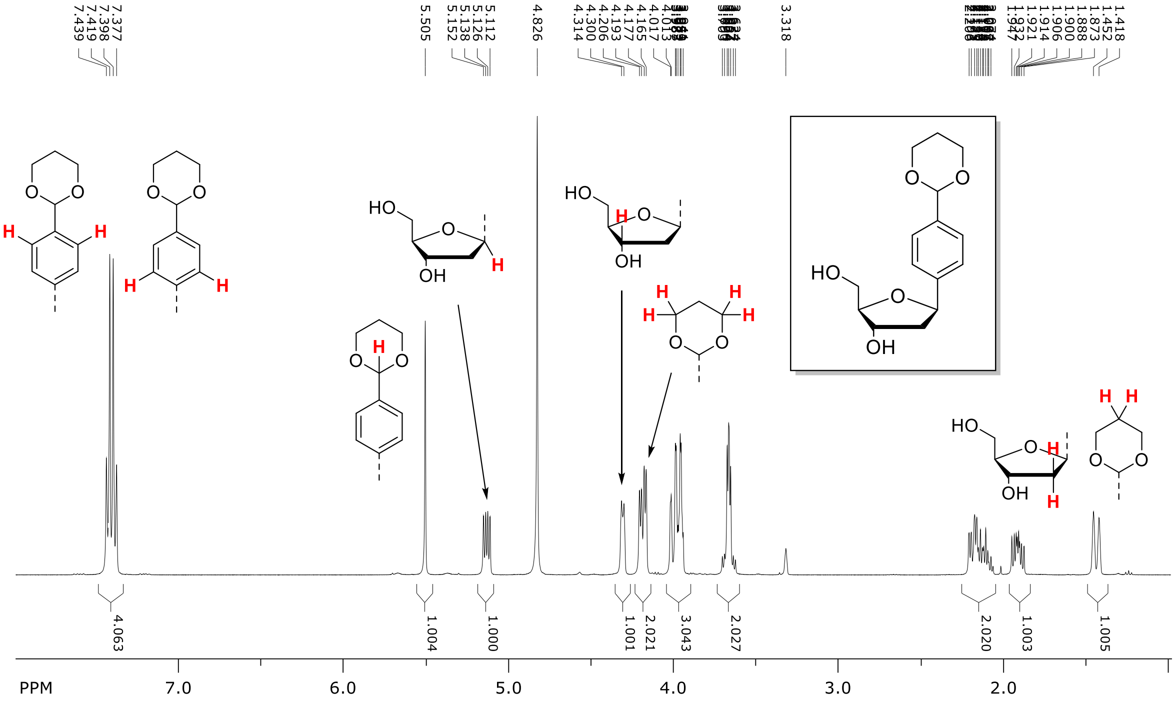
7.439
7.419
7.398
7.377
5.505
5.152
5.138
5.126
5.112
4.826
4.314
4.300
4.206
4.193
4.177
4.165
4.017
4.013
3.318
1.937
1.921
1.914
1.906
1.900
1.888
1.873
1.452
1.418
4.063
1.004
1.000
1.001
2.021
3.043
2.027
2.020
1.003
1.005
PPM
7.0
6.0
5.0
4.0
3.0
2.0

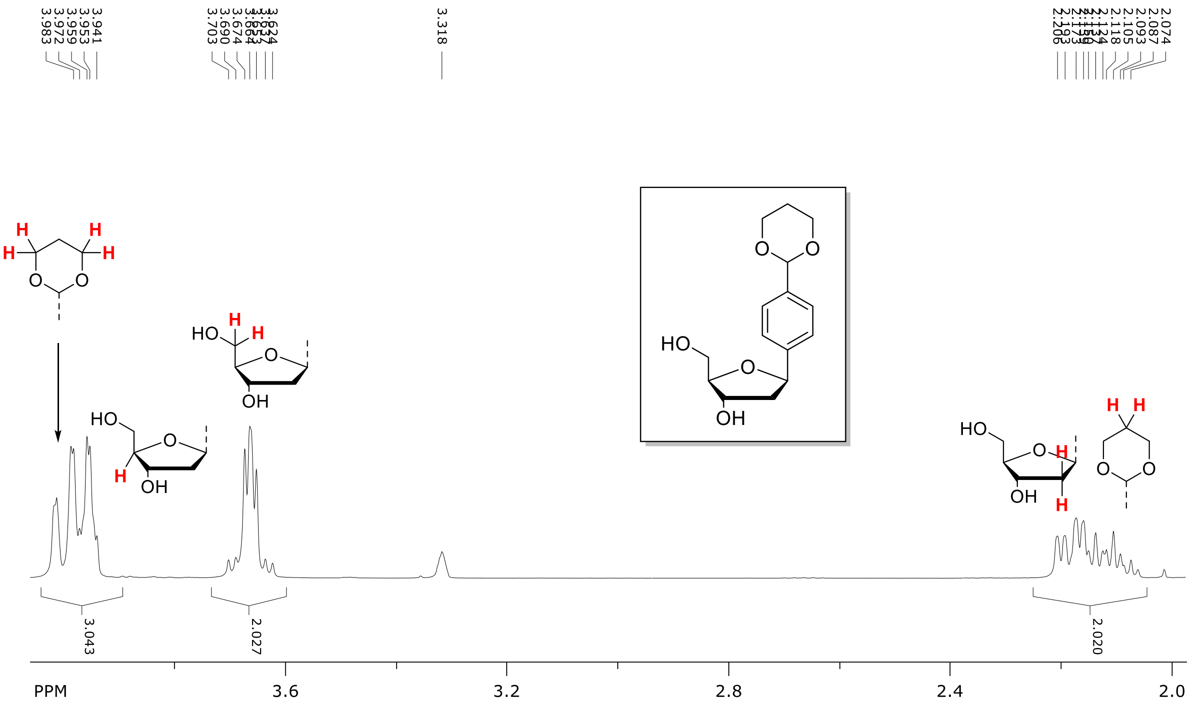
3.983
3.972
3.959
3.953
3.941
3.703
3.690
3.674
3.664
3.318
2.206
2.193
2.118
2.105
2.093
2.087
2.074
HO
OH
O
3.043
2.027
2.020
PPM
3.6
3.2
2.8
2.4
2.0

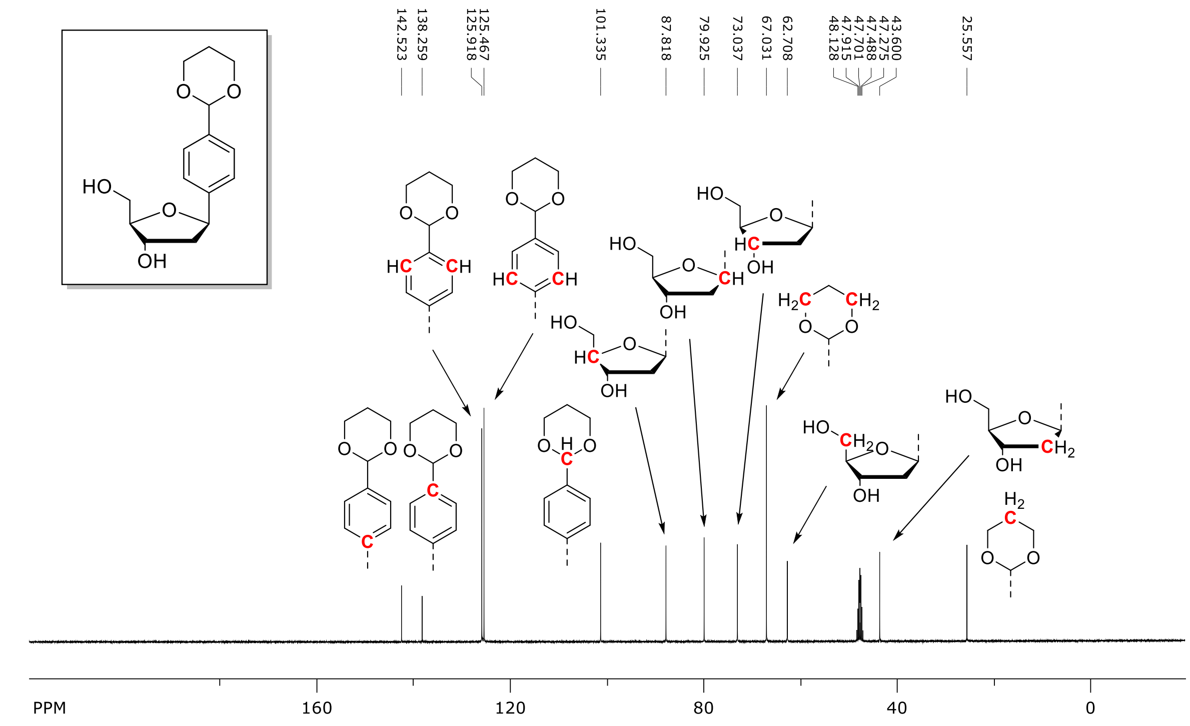
142.523
138.259
125.918
125.467
101.335
87.818
79.925
73.037
67.031
62.708
48.128
47.915
47.701
47.488
47.275
43.600
25.557
HO
OH
O
PPM
160
120
80
40
0

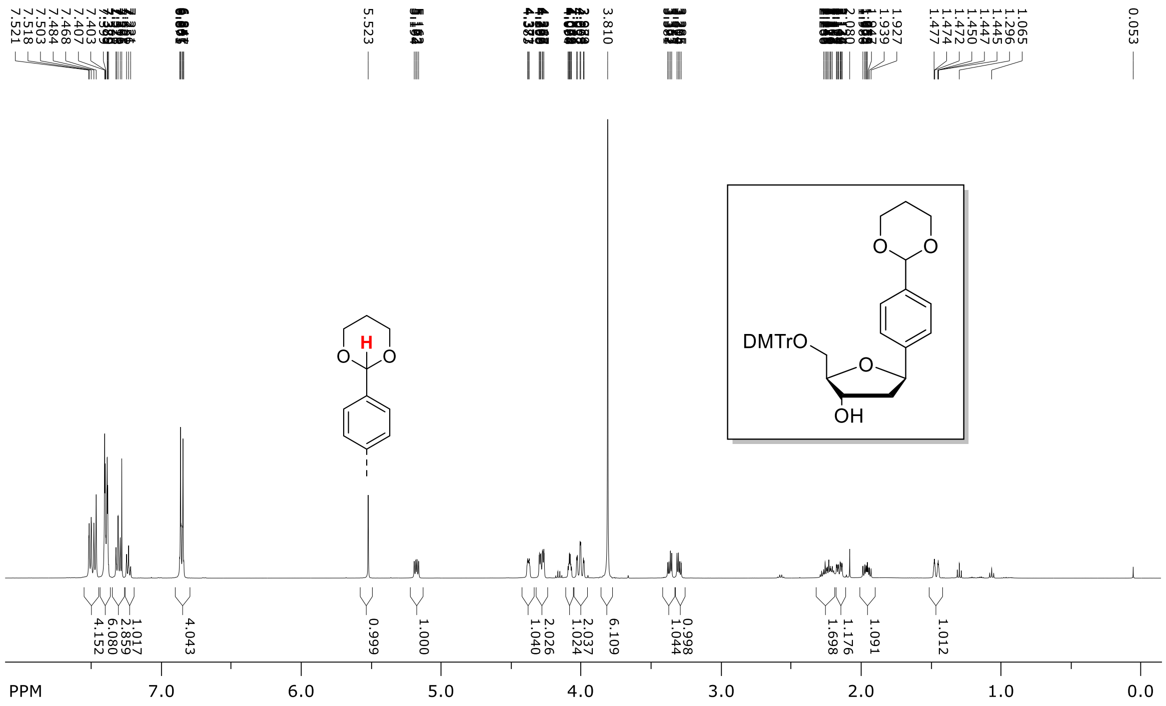

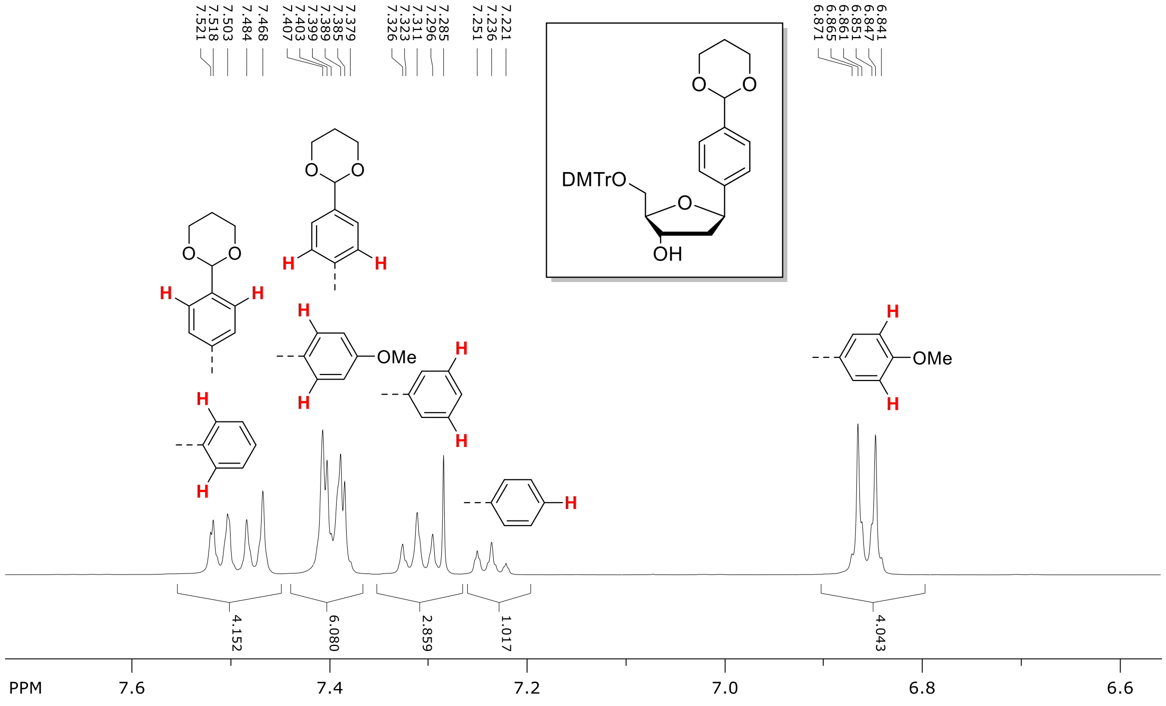

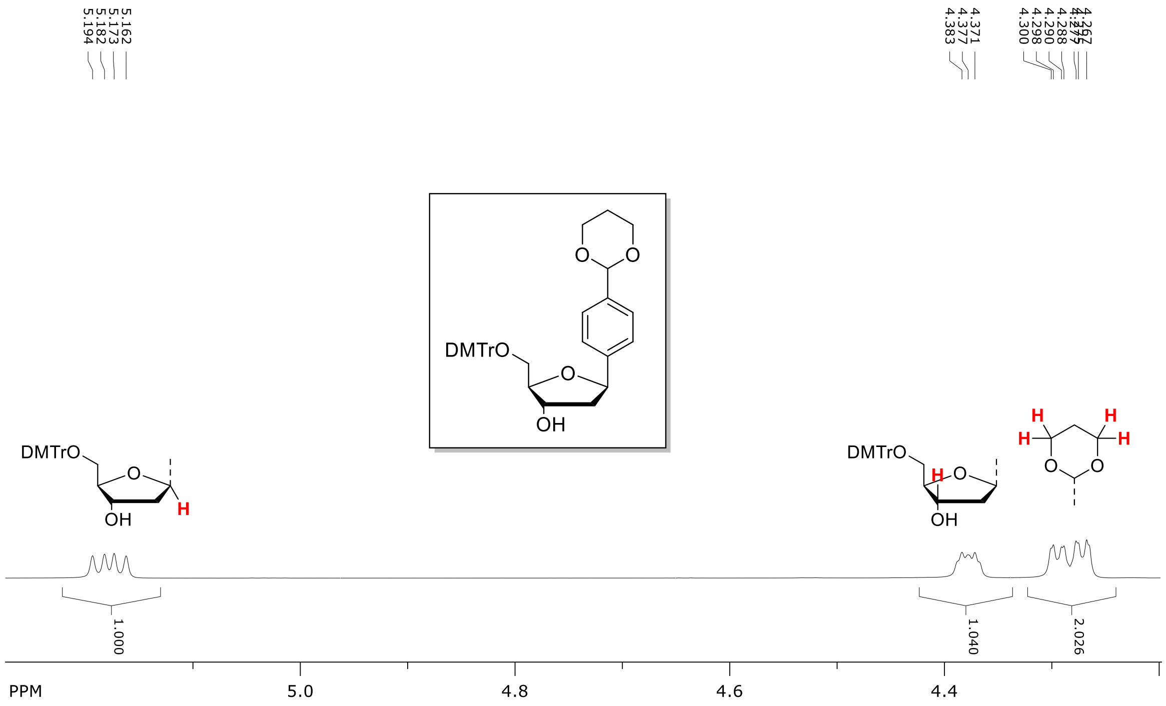
5.194
5.182
5.173
5.162
4.383
4.377
4.371
4.300
4.298
4.290
4.288
4.262
DMTrO
O
OH
H
1.000
1.040
2.026
PPM
5.0
4.8
4.6
4.4

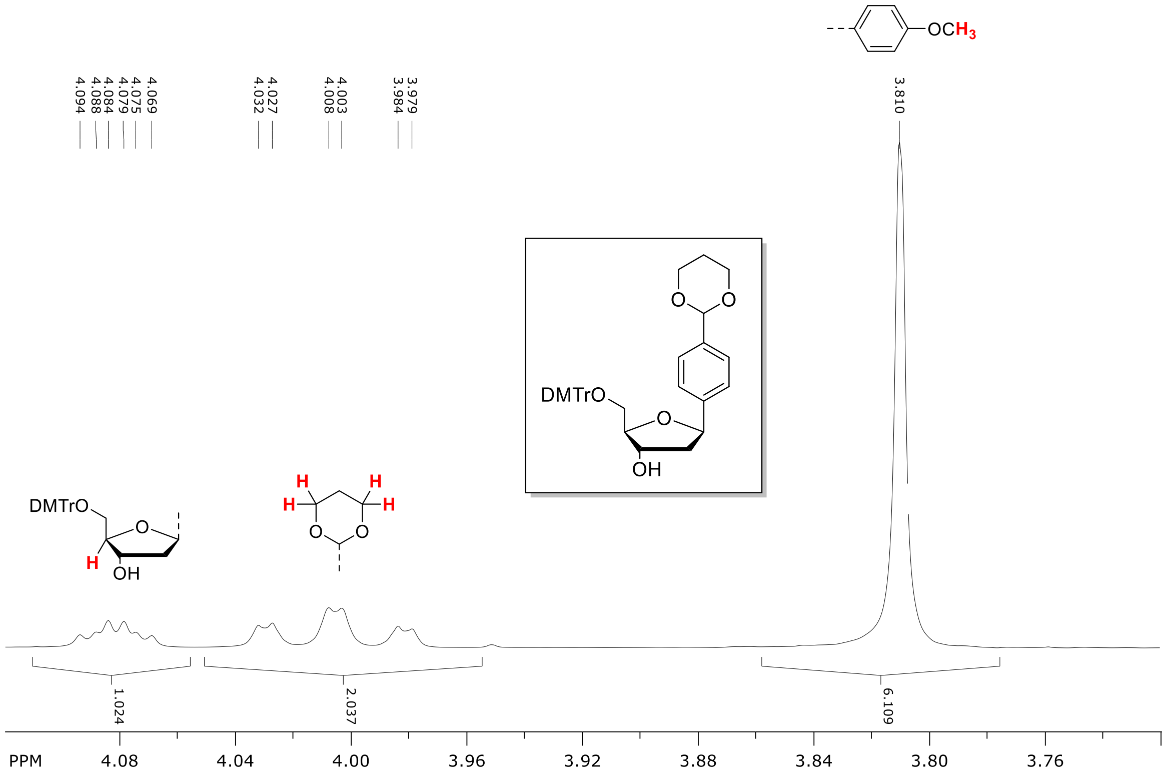
OCH3
4.094
4.088
4.084
4.079
4.075
4.069
4.032
4.027
4.008
4.003
3.984
3.979
3.810
DMTrO
O
OH
H
1.024
2.037
6.109
PPM
4.08
4.04
4.00
3.96
3.92
3.88
3.84
3.80
3.76

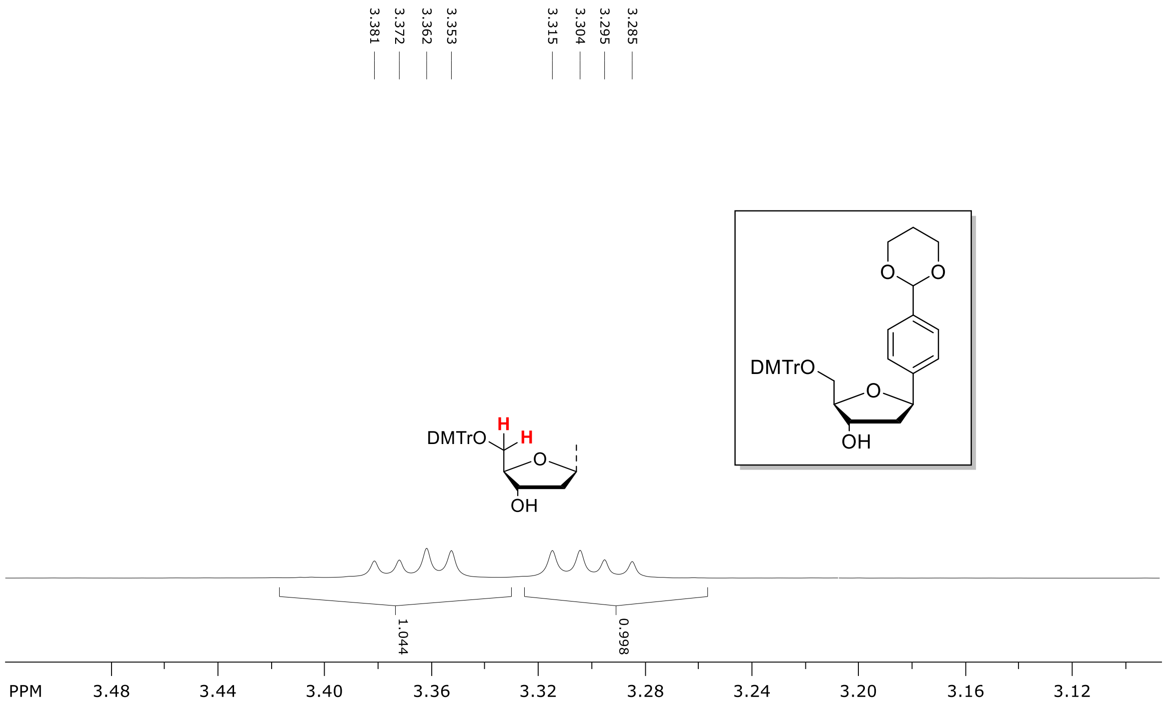
3.381
3.372
3.362
3.353
3.315
3.304
3.295
3.285
DMTrO
O
OH
1.044
0.998
PPM
3.48
3.44
3.40
3.36
3.32
3.28
3.24
3.20
3.16
3.12

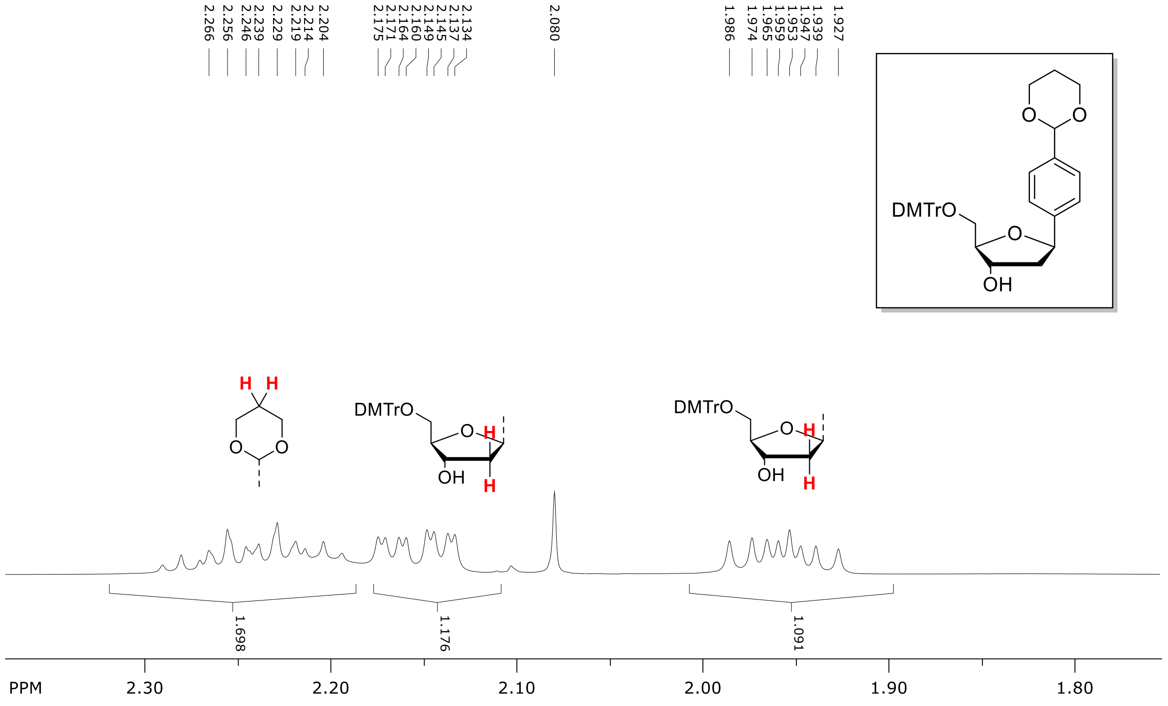
2.266
2.256
2.246
2.239
2.229
2.219
2.214
2.204
2.175
2.171
2.164
2.160
2.149
2.145
2.137
2.134
2.080
1.986
1.974
1.965
1.959
1.953
1.947
1.939
1.927
DMTrO
O
OH
1.698
1.176
1.091
PPM
2.30
2.20
2.10
2.00
1.90
1.80

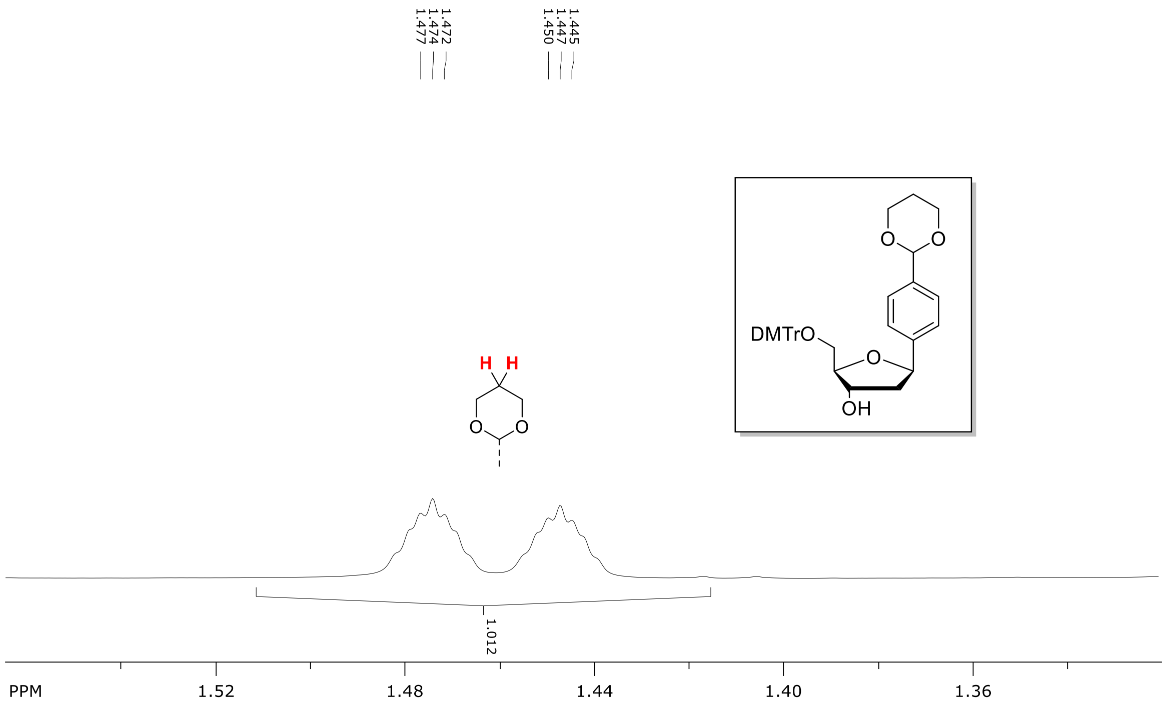
1.477
1.474
1.472
1.450
1.447
1.445
DMTrO
O
OH
1.012
PPM
1.52
1.48
1.44
1.40
1.36

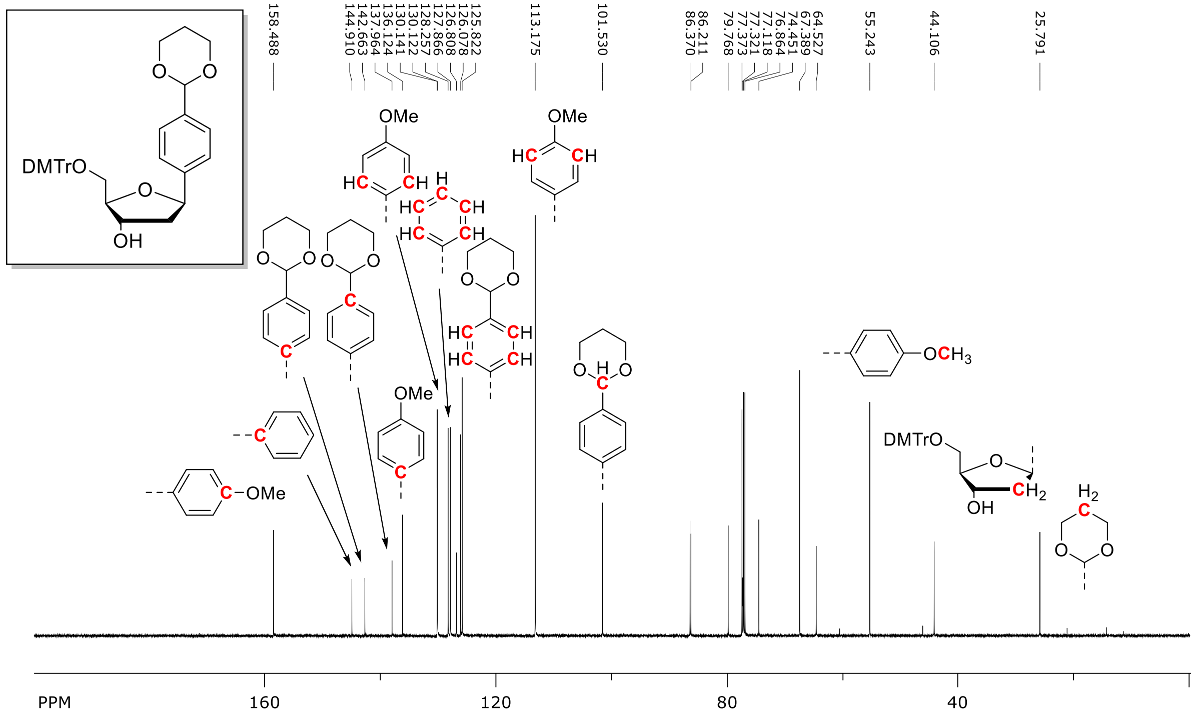
158.488
144.910
142.663
137.964
136.124
130.141
130.122
128.257
127.866
126.808
126.078
125.822
113.175
101.530
86.211
86.370
79.768
77.373
77.321
77.118
76.864
74.451
67.389
64.527
55.243
44.106
25.791
DMTrO
OH
OMe
OCH3
PPM
160
120
80
40

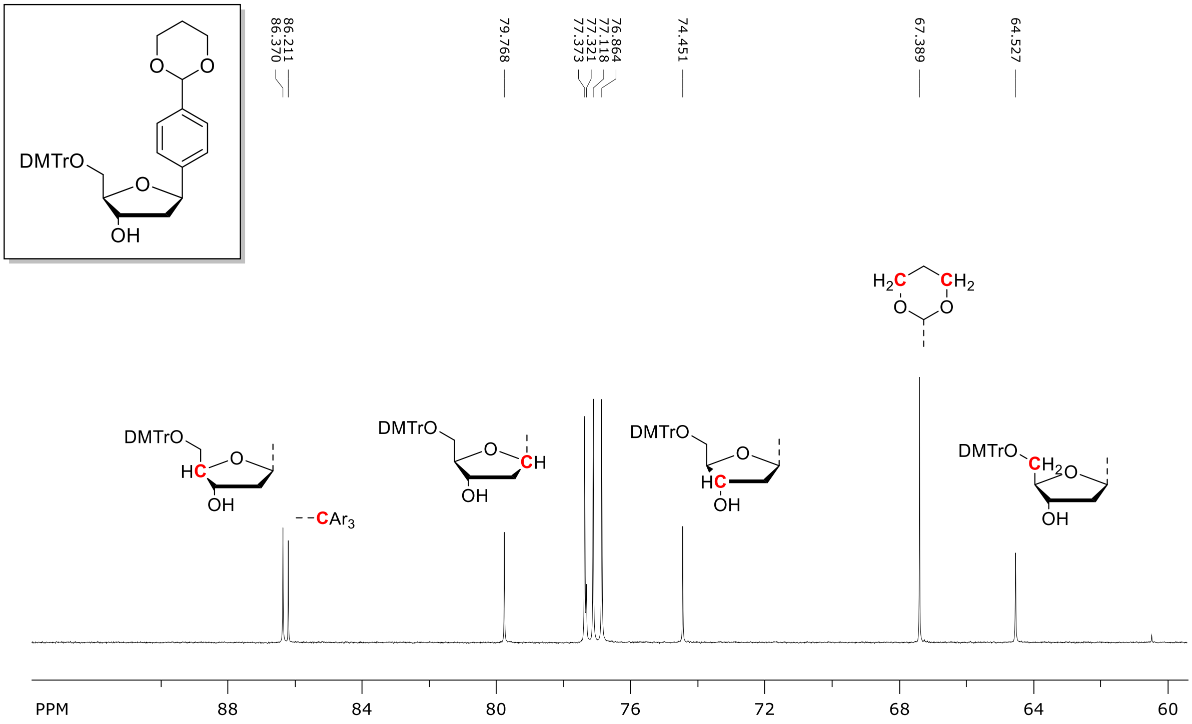

86.211
86.370
79.768
77.373
77.321
77.118
76.864
74.451
67.389
64.527
DMTrO
OH
CAr3
PPM
88
84
80
76
72
68
64
60


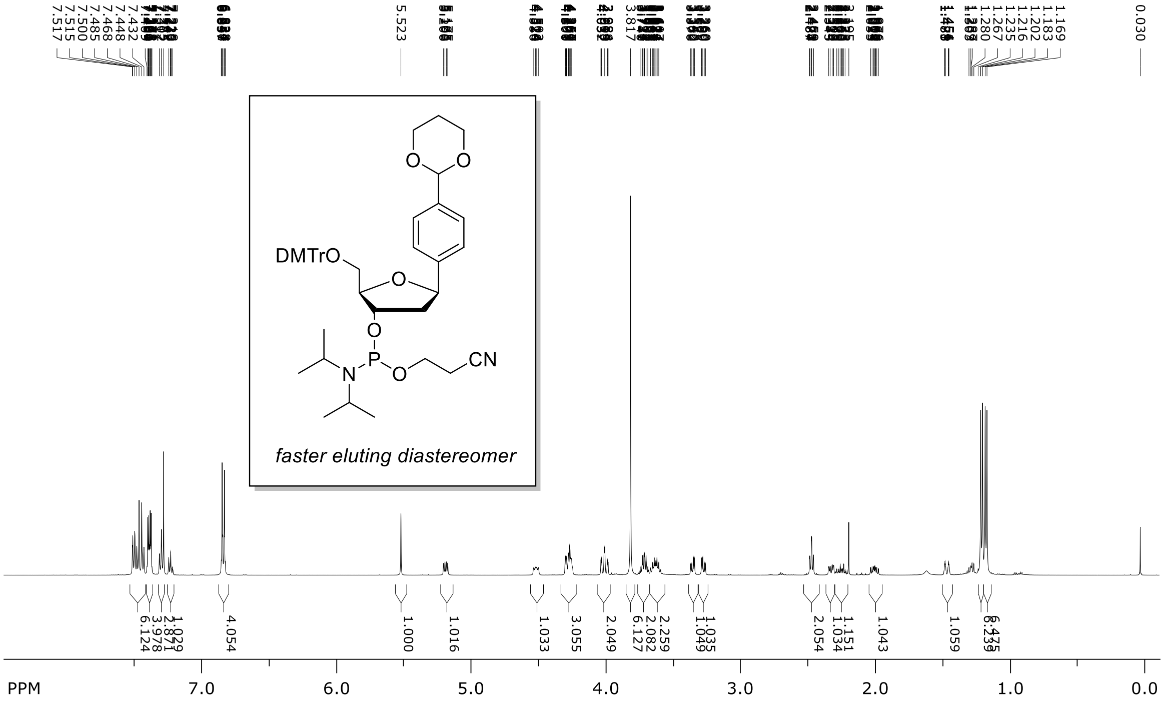

7.517
7.515
7.500
7.485
7.468
7.448
7.432
5.523
3.817
1.280
1.267
1.235
1.216
1.202
1.183
1.169
0.030
DMTrO
O
P
N
CN
faster eluting diastereomer
6.124
3.978
2.871
1.029
4.054
1.000
1.016
1.033
3.055
2.049
6.127
2.082
2.259
1.049
1.035
2.054
1.034
1.151
1.043
1.059
PPM
7.0
6.0
5.0
4.0
3.0
2.0
1.0
0.0

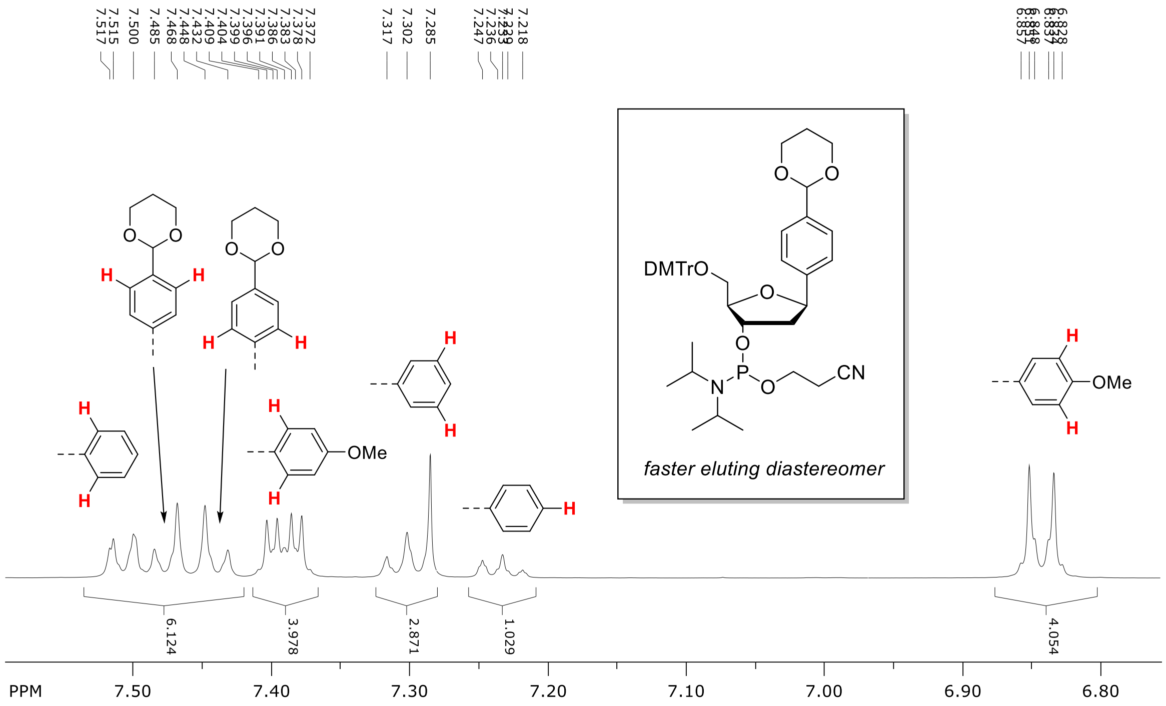

faster eluting diastereomer
DMTrO
CN
OMe
PPM
7.50
7.40
7.30
7.20
7.10
7.00
6.90
6.80
6.124
3.978
2.871
1.029
4.054


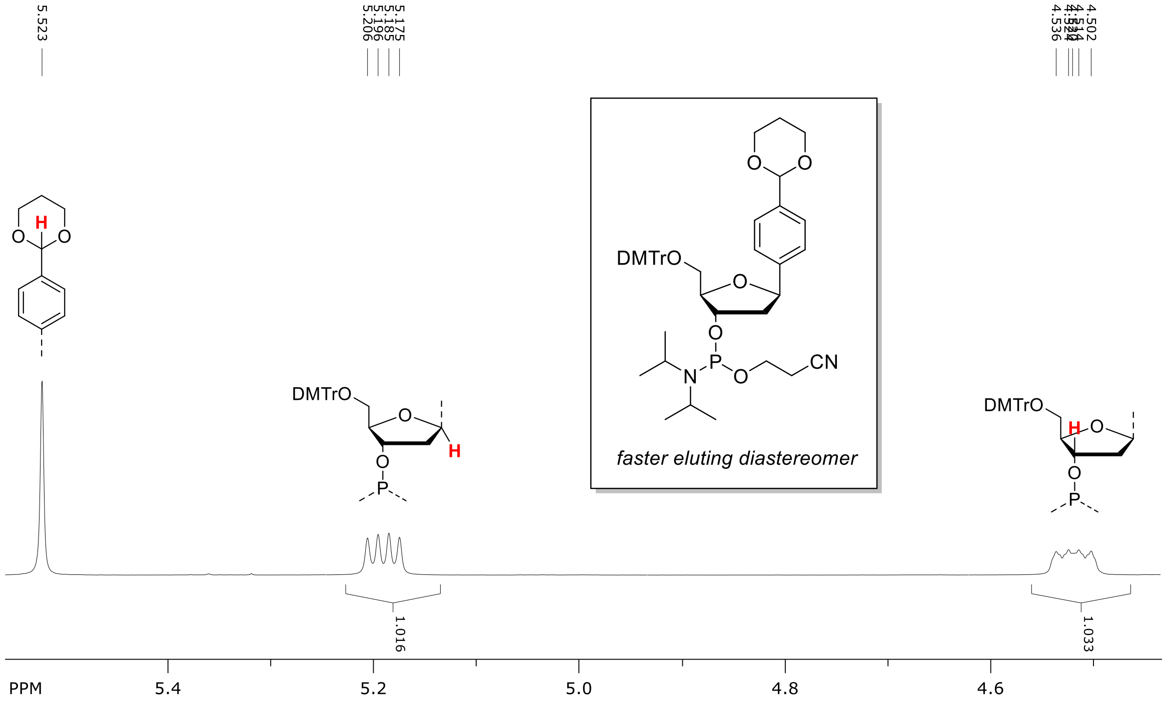

faster eluting diastereomer
DMTrO
CN
5.523
PPM
5.4
5.2
5.0
4.8
4.6
1.016
1.033

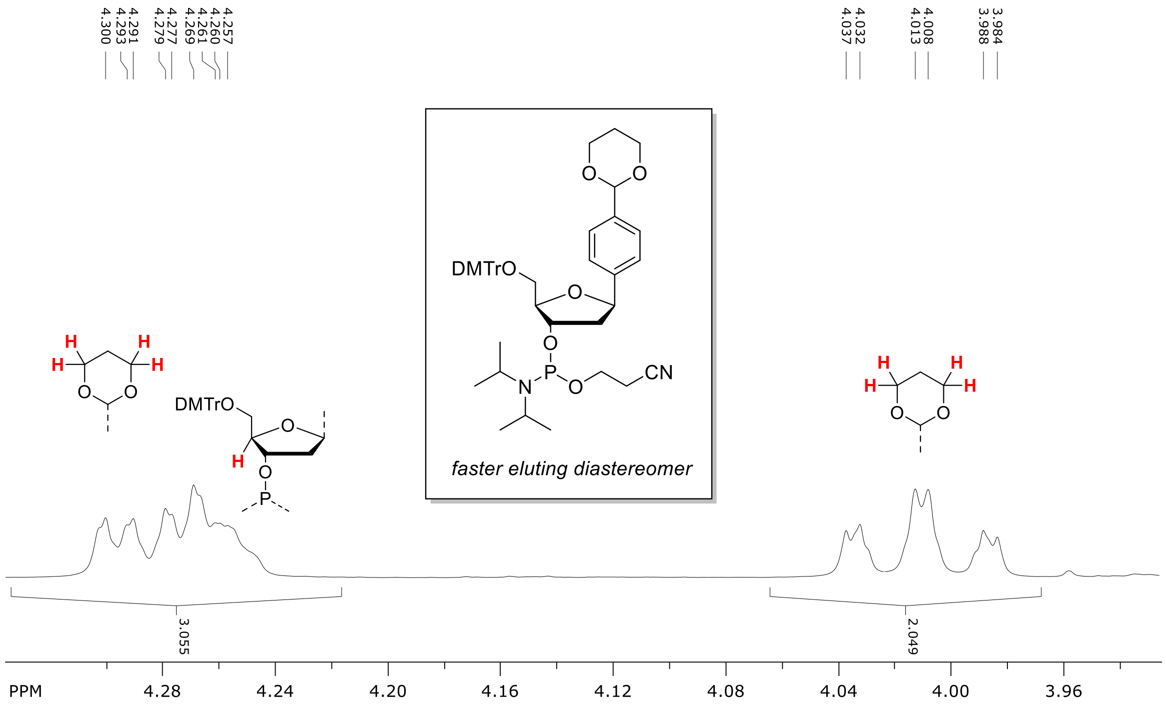

4.300
4.293
4.291
4.279
4.277
4.269
4.261
4.260
4.257
4.037
4.032
4.013
4.008
3.988
3.984
DMTrO
faster eluting diastereomer
3.055
2.049
PPM
4.28
4.24
4.20
4.16
4.12
4.08
4.04
4.00
3.96


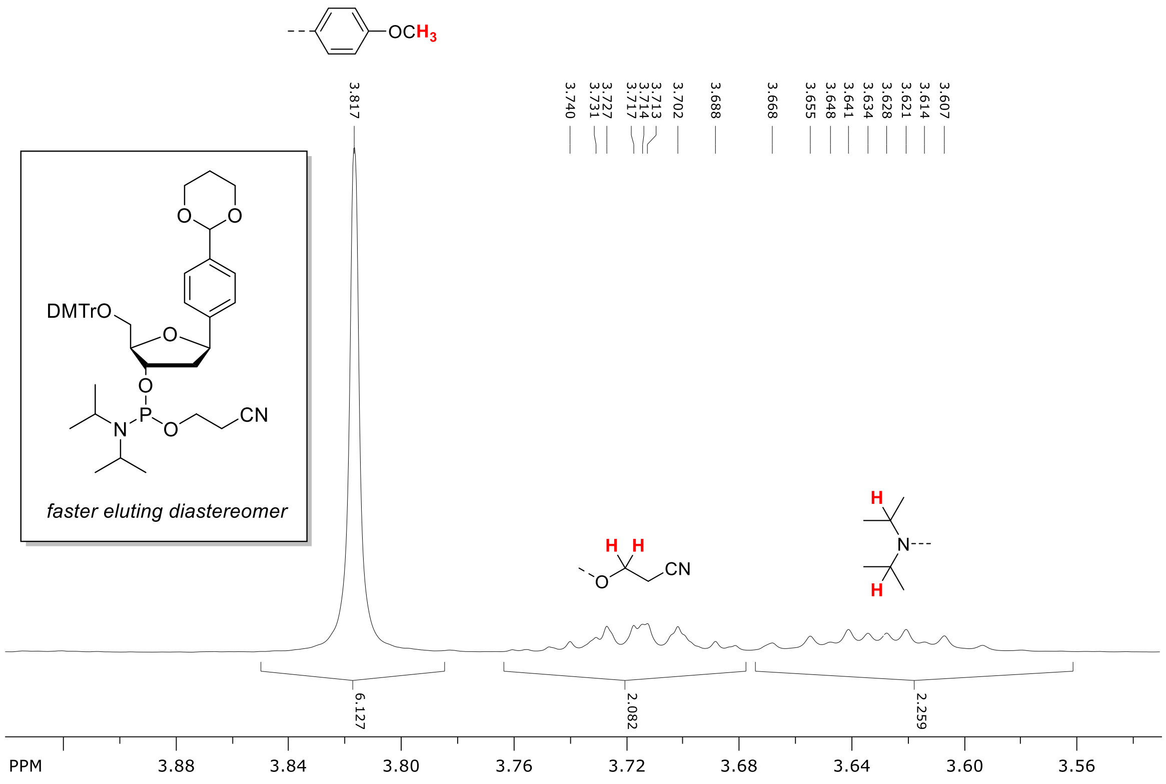

OCH3
3.817
3.740
3.731
3.727
3.717
3.714
3.713
3.702
3.688
3.668
3.655
3.648
3.641
3.634
3.628
3.621
3.614
3.607
DMTrO
faster eluting diastereomer
6.127
2.082
2.259
PPM
3.88
3.84
3.80
3.76
3.72
3.68
3.64
3.60
3.56

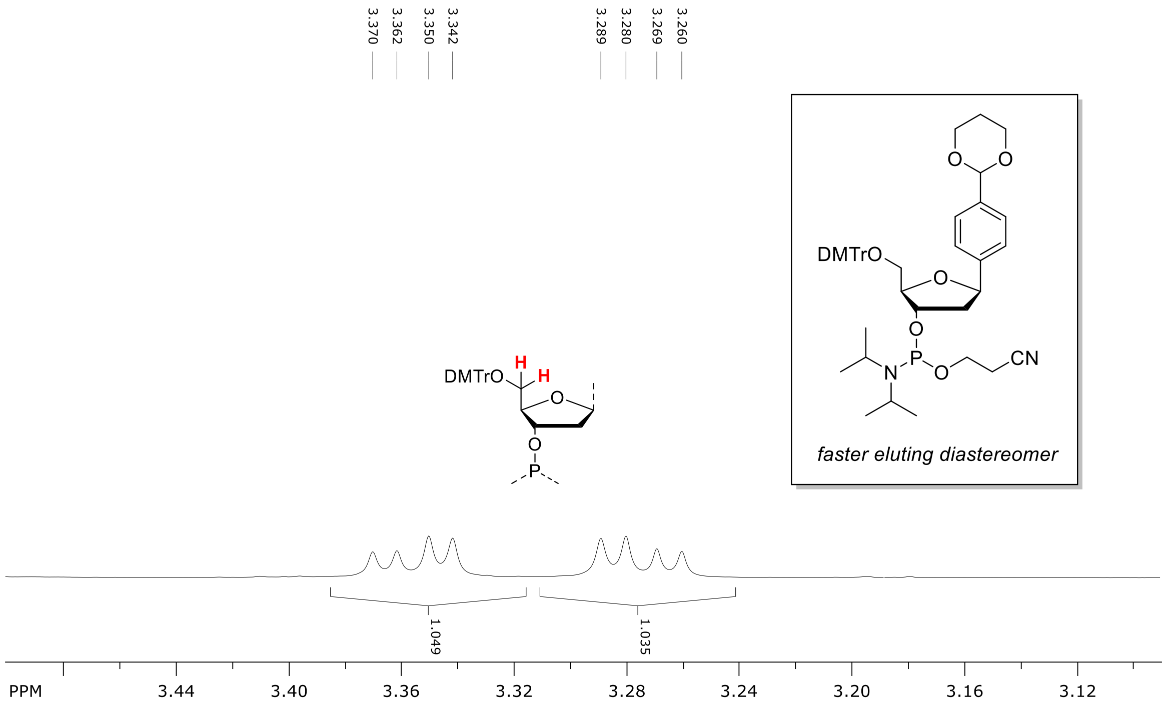
3.370
3.362
3.350
3.342
3.289
3.280
3.269
3.260
DMTrO
faster eluting diastereomer
1.049
1.035
PPM
3.44
3.40
3.36
3.32
3.28
3.24
3.20
3.16
3.12

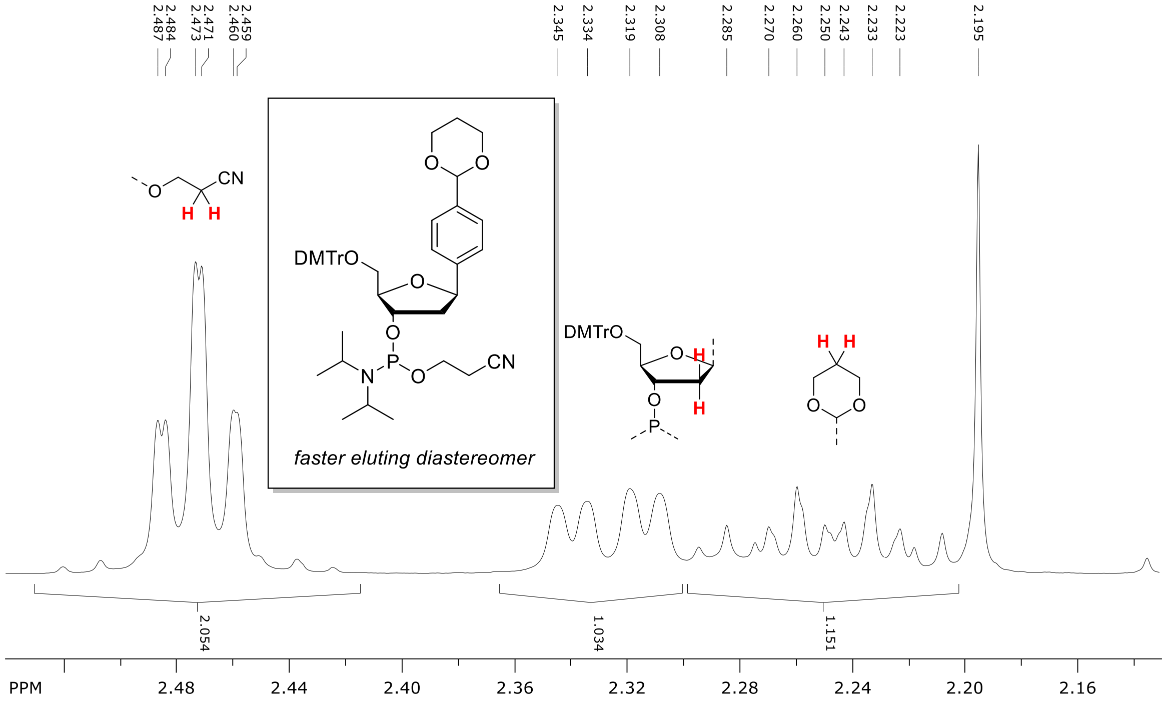
2.487
2.484
2.473
2.471
2.460
2.459
2.345
2.334
2.319
2.308
2.285
2.270
2.260
2.250
2.243
2.233
2.223
2.195
CN
DMTrO
faster eluting diastereomer
2.054
1.034
1.151
PPM
2.48
2.44
2.40
2.36
2.32
2.28
2.24
2.20
2.16

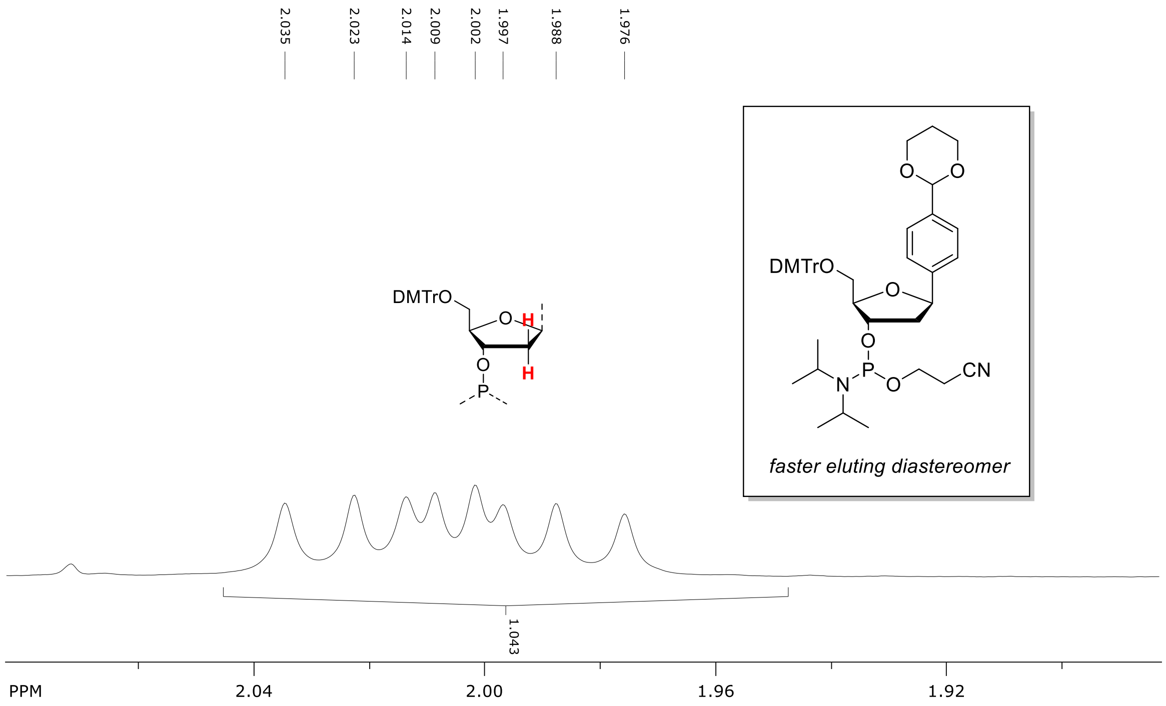
2.035
2.023
2.014
2.009
2.002
1.997
1.988
1.976
DMTrO
O
H
H
O
P
faster eluting diastereomer
CN
N
1.043
PPM
2.04
2.00
1.96
1.92

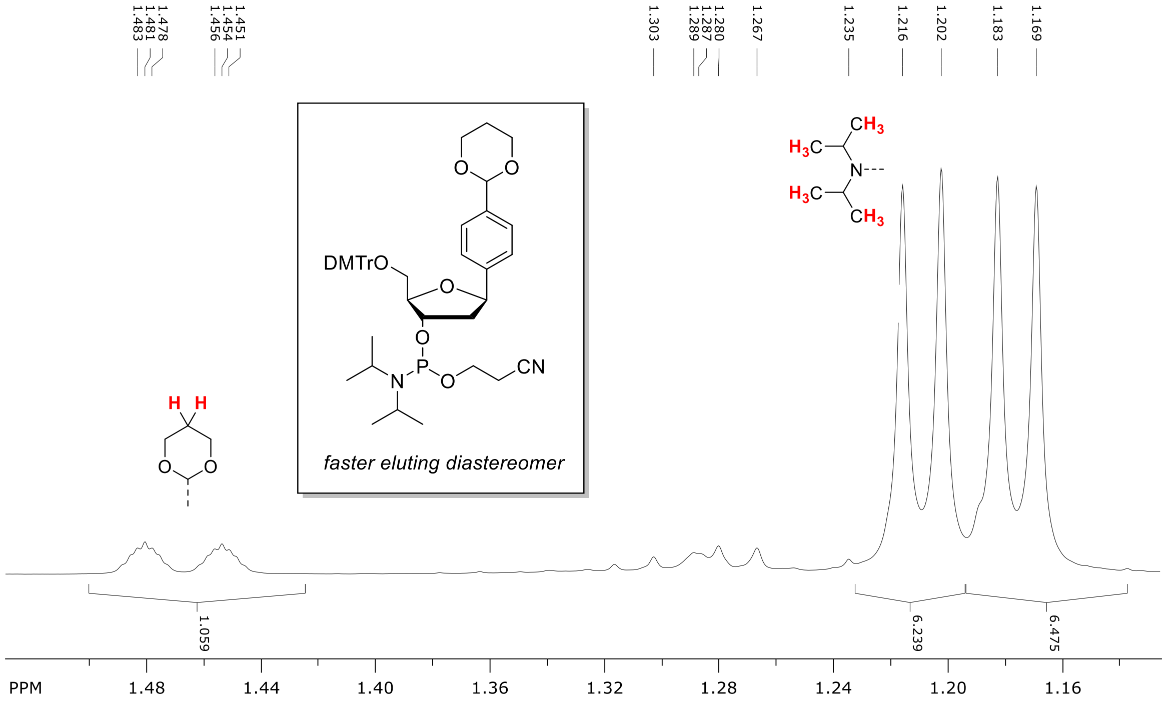
1.483
1.481
1.478
1.456
1.454
1.451
1.303
1.289
1.287
1.280
1.267
1.235
1.216
1.202
1.183
1.169
$H_3C$
$CH_3$
$H_3C$
$CH_3$
N
H H
O O
DMTrO
CN
faster eluting diastereomer
1.059
6.239
6.475
PPM
1.48
1.44
1.40
1.36
1.32
1.28
1.24
1.20
1.16

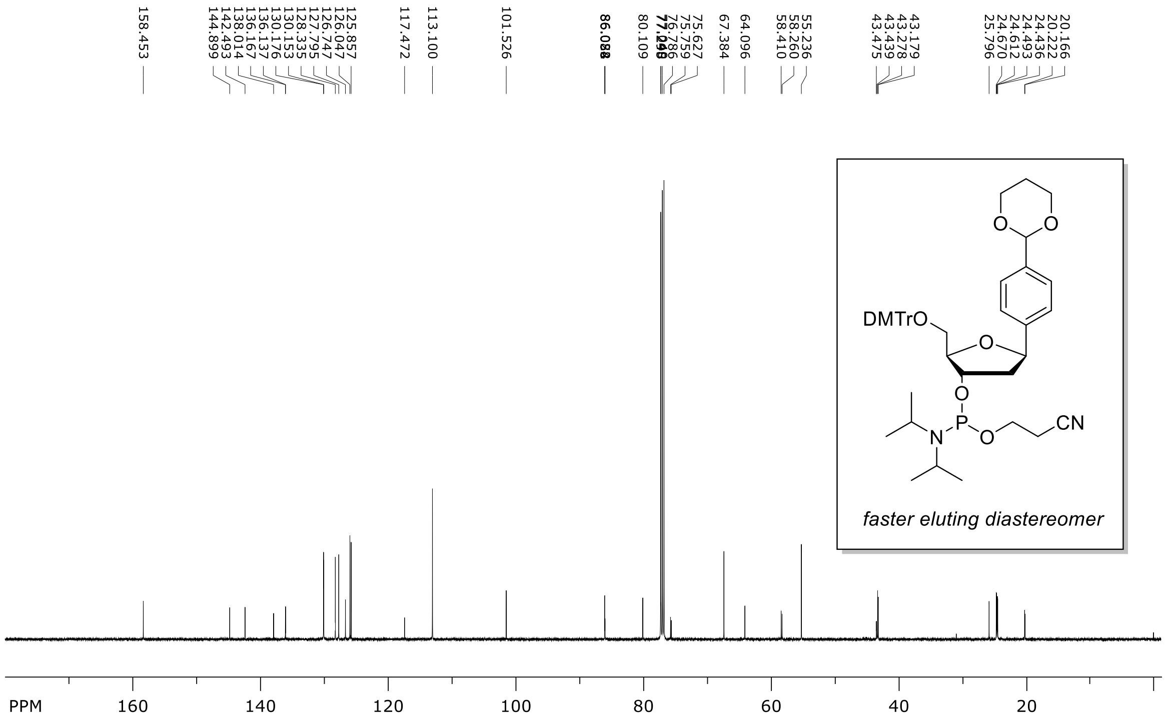
158.453
144.899
142.493
138.014
136.167
136.137
130.176
130.153
128.335
127.795
126.747
126.047
125.857
117.472
113.100
101.526
80.109
75.759
75.627
67.384
64.096
58.410
58.260
55.236
43.475
43.439
43.278
43.179
25.796
24.670
24.612
24.493
24.436
20.222
20.166
DMTrO
O
O
O
O
P
N
CN
faster eluting diastereomer
PPM
160
140
120
100
80
60
40
20

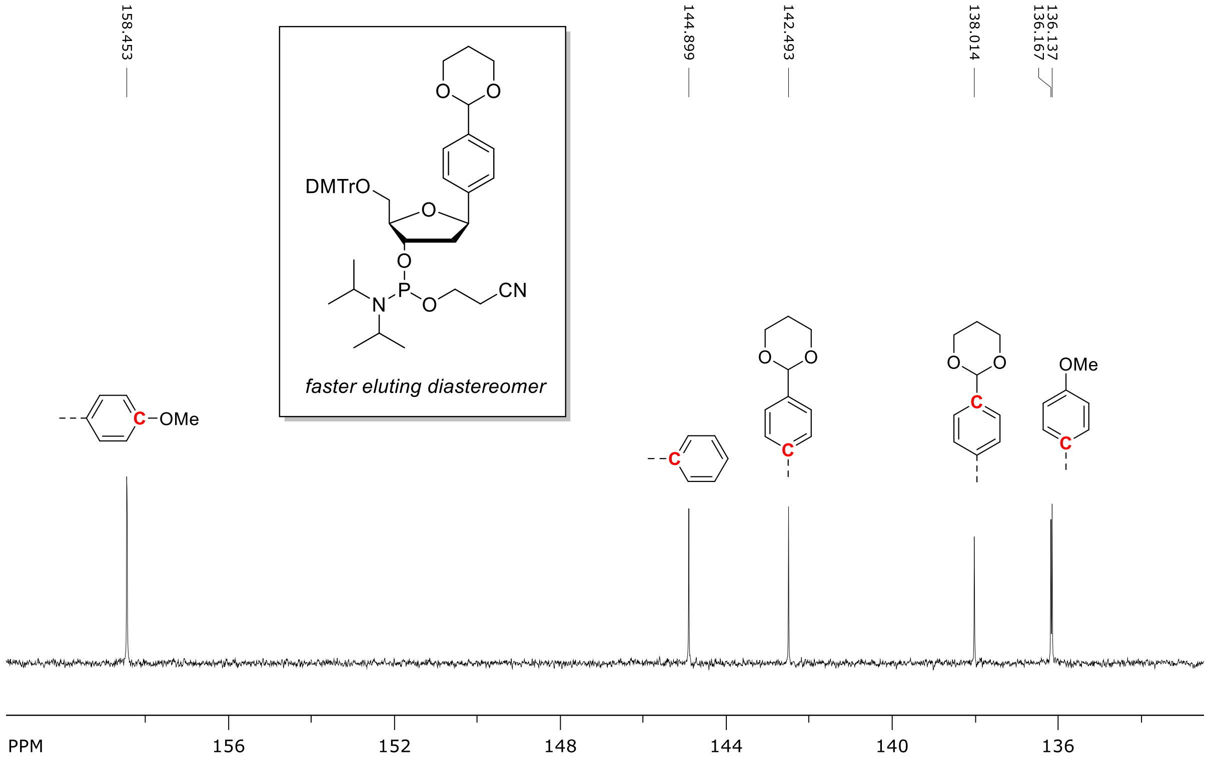
158.453
144.899
142.493
138.014
136.167
136.137
DMTrO
O
O
O
O
P
N
CN
faster eluting diastereomer
C
OMe
PPM
156
152
148
144
140
136

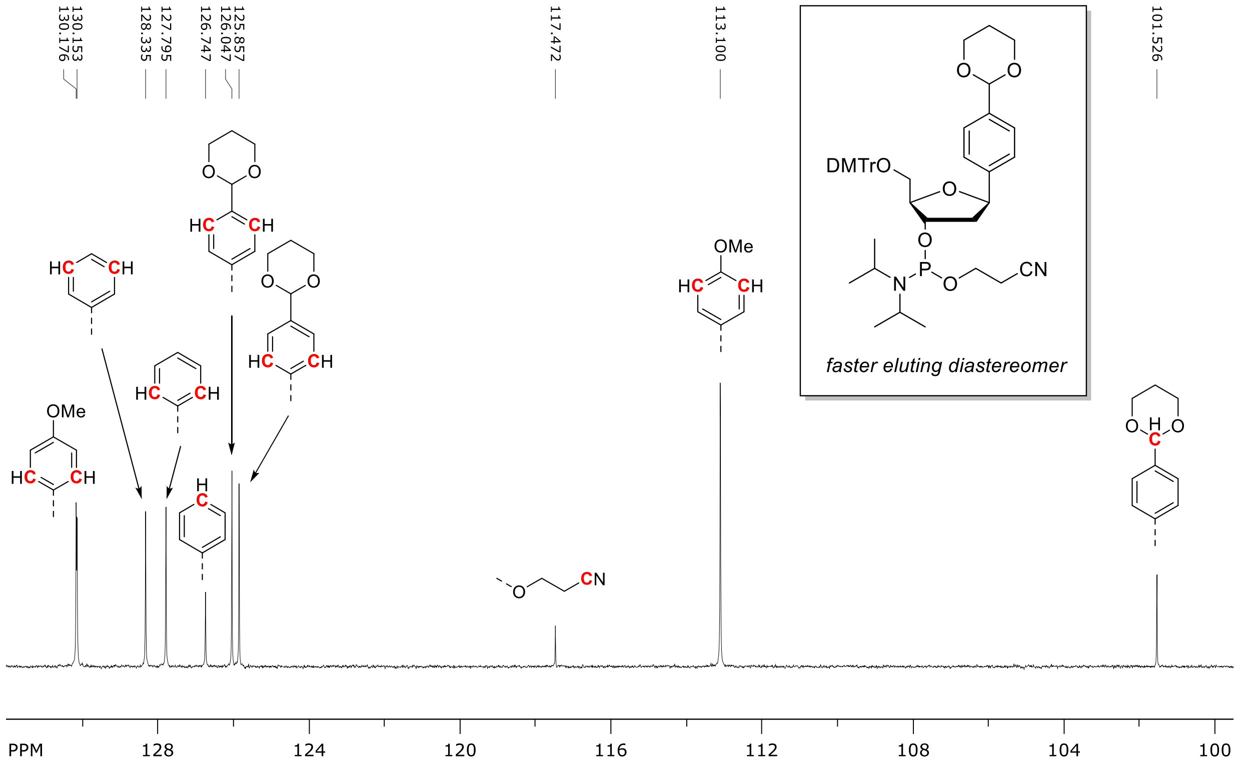

130.153
130.176
128.335
127.795
126.747
126.047
125.857
117.472
113.100
101.526
faster eluting diastereomer
PPM
128
124
120
116
112
108
104
100


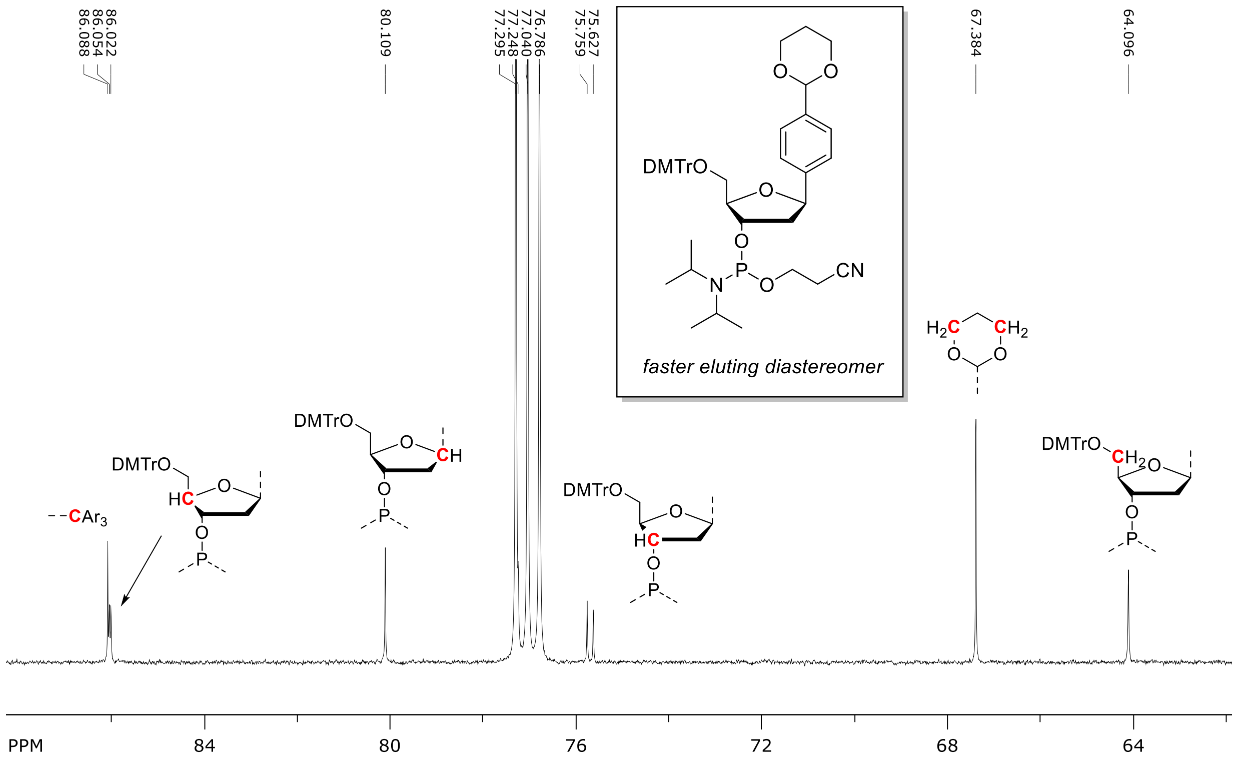

86.022
86.054
86.088
80.109
77.295
77.248
77.040
76.786
75.627
75.759
67.384
64.096
faster eluting diastereomer
PPM
84
80
76
72
68
64

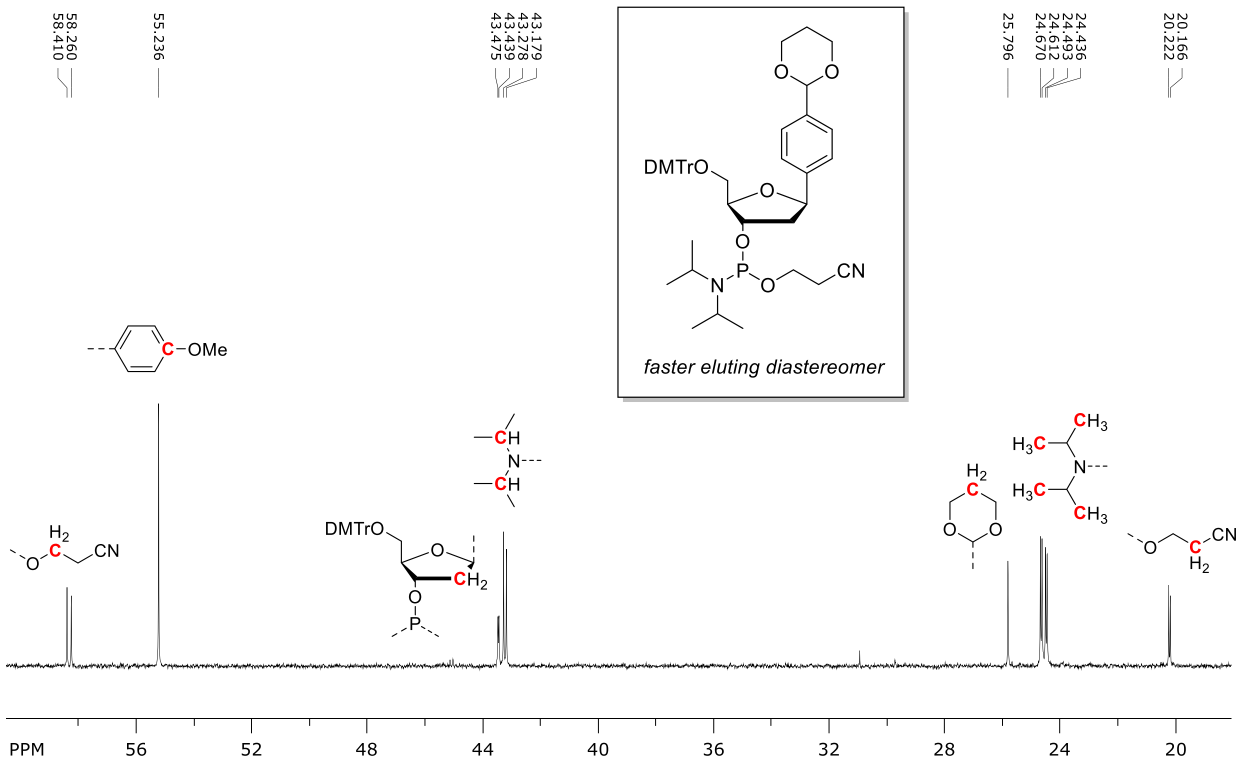
58.410
58.260
55.236
43.475
43.439
43.278
43.179
25.796
24.670
24.612
24.493
24.436
20.222
20.166
DMTrO
O
O
O
O
P
N
O
CN
faster eluting diastereomer
OMe
DMTrO
CH
N
CH
H2
CN
CH2
H3C
CH3
PPM
56
52
48
44
40
36
32
28
24
20

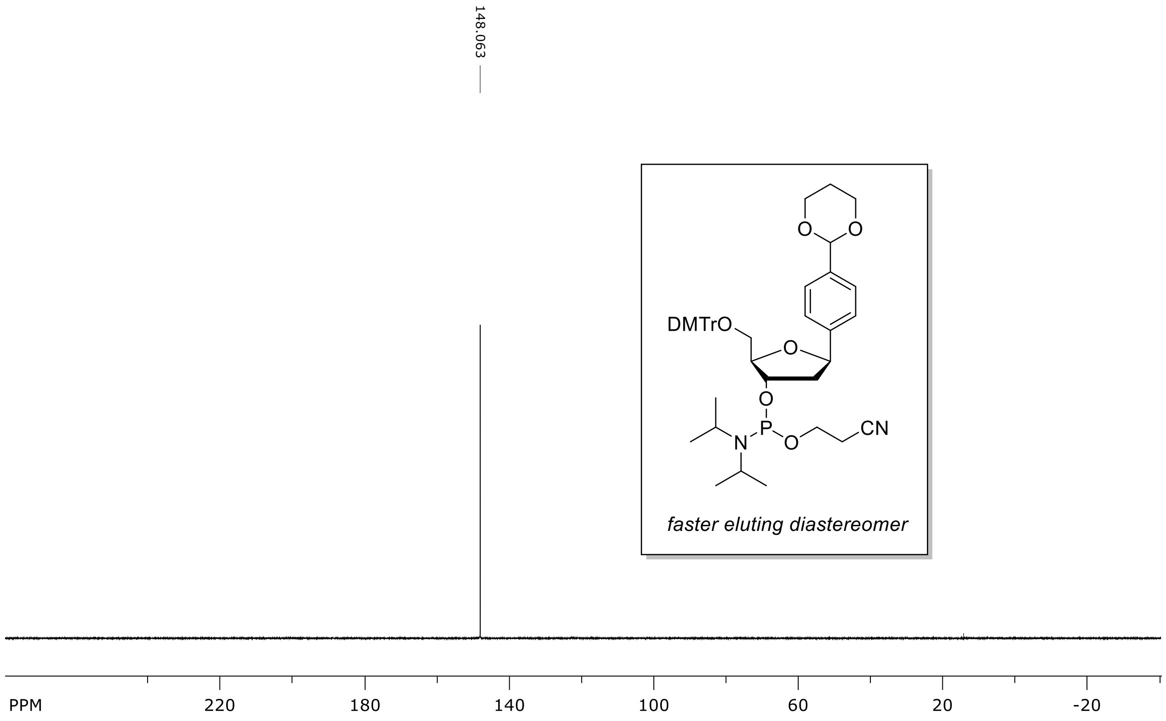
148.063
DMTrO
O
O
O
O
P
N
O
CN
faster eluting diastereomer
PPM
220
180
140
100
60
20
-20

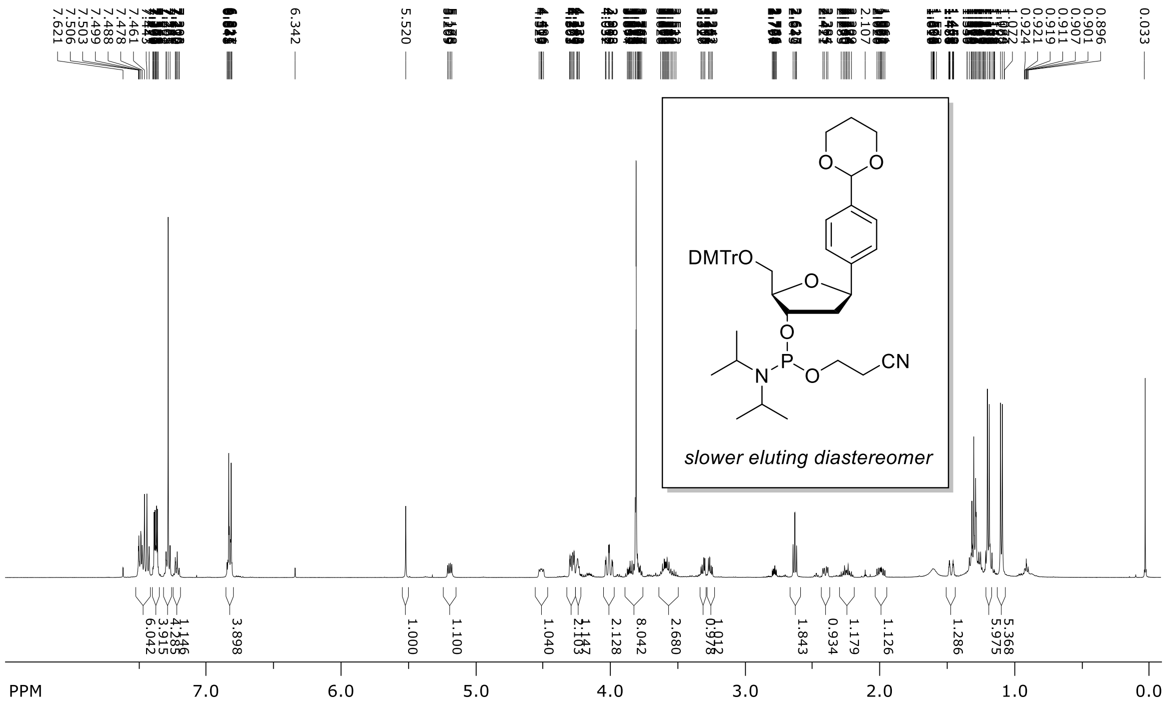
DMTrO
O
CN
P
N
slower eluting diastereomer
6.042
3.915
3.898
1.000
1.100
1.040
2.128
8.042
2.680
1.843
0.934
1.179
1.126
1.286
5.975
5.368
PPM
7.0
6.0
5.0
4.0
3.0
2.0
1.0
0.0

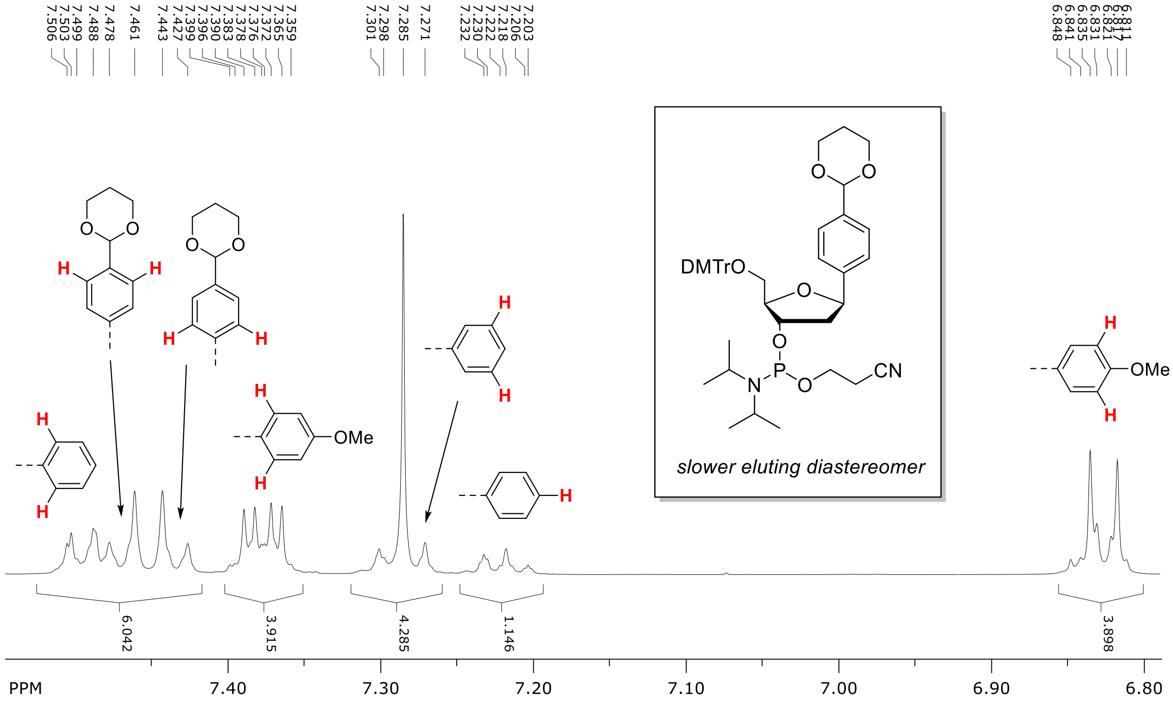
DMTrO
slower eluting diastereomer
OMe
H
6.042
3.915
4.285
1.146
3.898
PPM
7.40
7.30
7.20
7.10
7.00
6.90
6.80

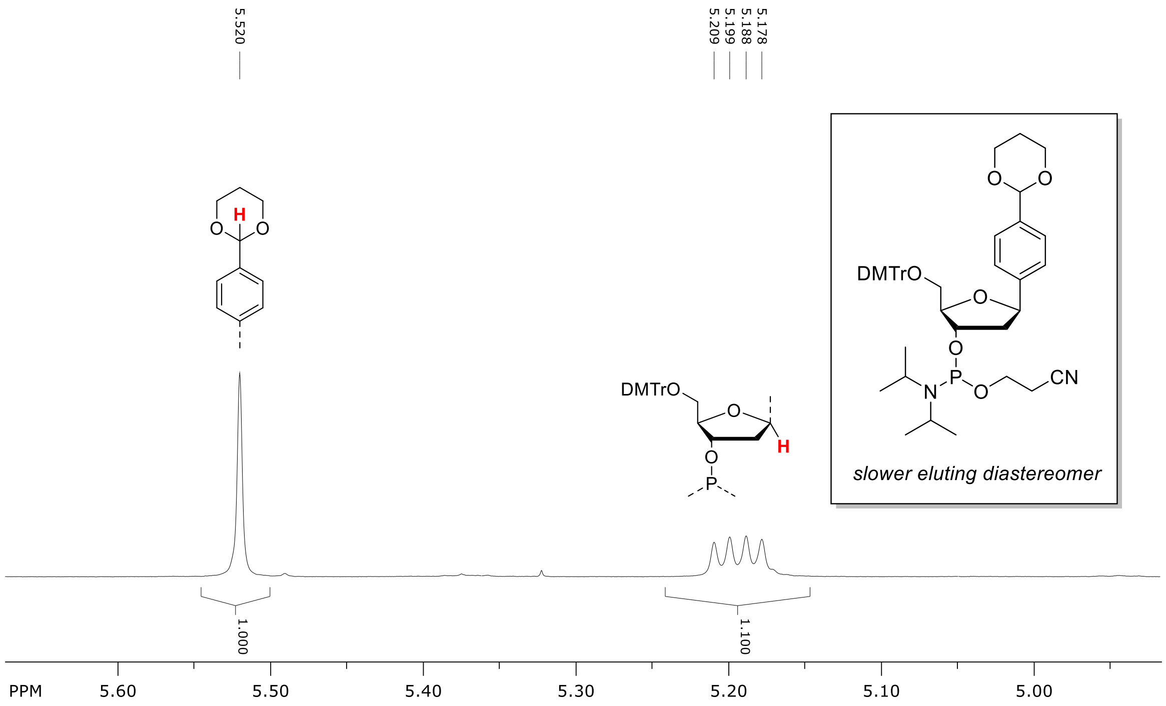
5.520
5.209
5.199
5.188
5.178
DMTrO
slower eluting diastereomer
CN
1.000
1.100
PPM
5.60
5.50
5.40
5.30
5.20
5.10
5.00

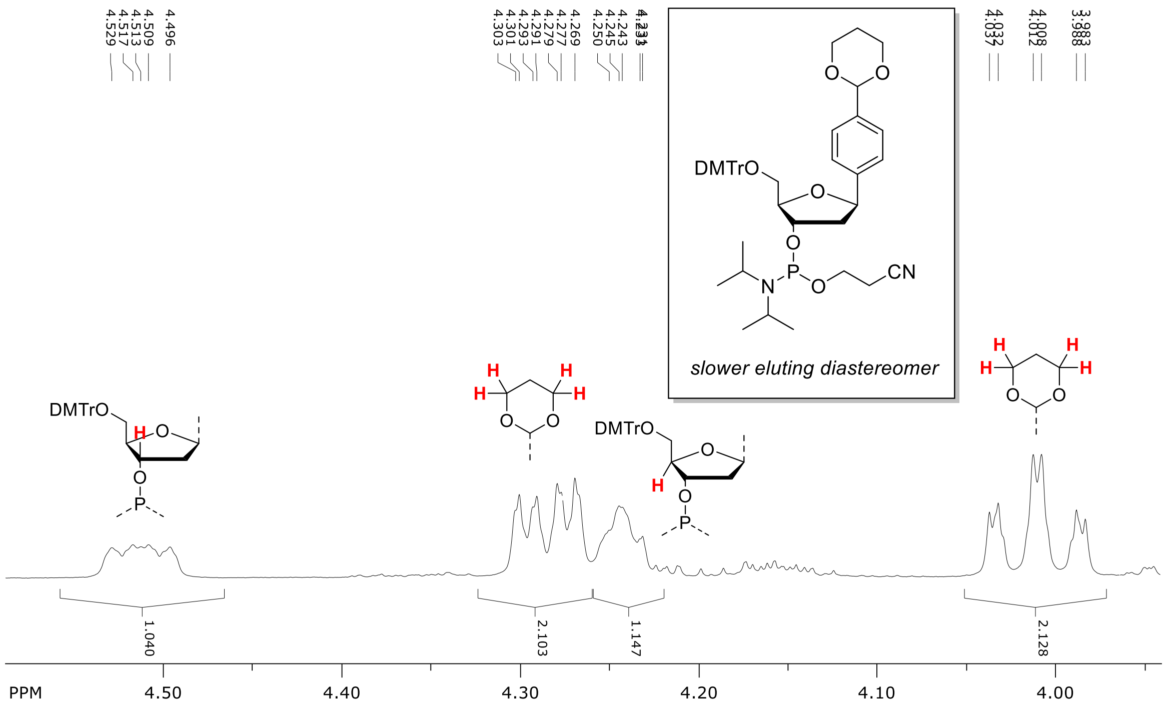
4.529
4.517
4.513
4.509
4.496
4.303
4.301
4.293
4.291
4.279
4.277
4.269
4.250
4.245
4.243
DMTrO
slower eluting diastereomer
CN
1.040
2.103
1.147
2.128
PPM
4.50
4.40
4.30
4.20
4.10
4.00

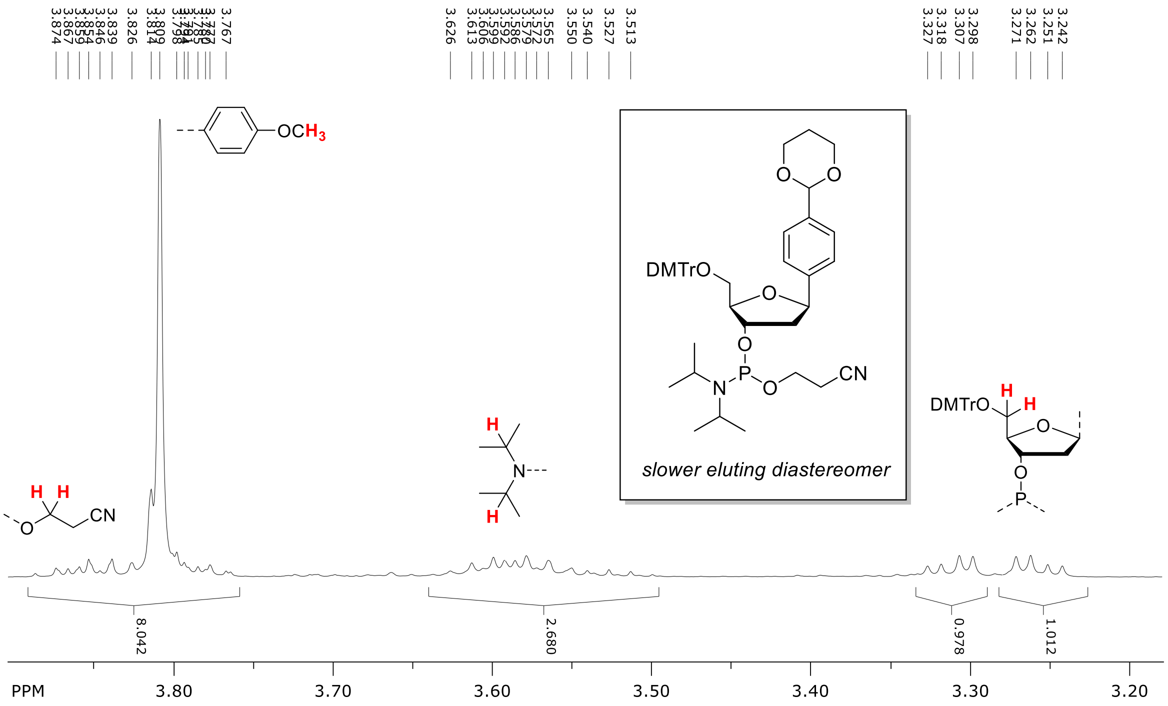
3.874 3.867 3.859 3.854 3.846 3.839 3.826 3.814 3.809 3.798 3.767
3.626 3.613 3.606 3.599 3.592 3.586 3.579 3.572 3.565 3.550 3.540 3.527 3.513
3.327 3.318 3.307 3.298 3.271 3.262 3.251 3.242
OCH3
H H
CN
O
N
DMTrO
O
P
slower eluting diastereomer
8.042
2.680
0.978
1.012
PPM
3.80
3.70
3.60
3.50
3.40
3.30
3.20

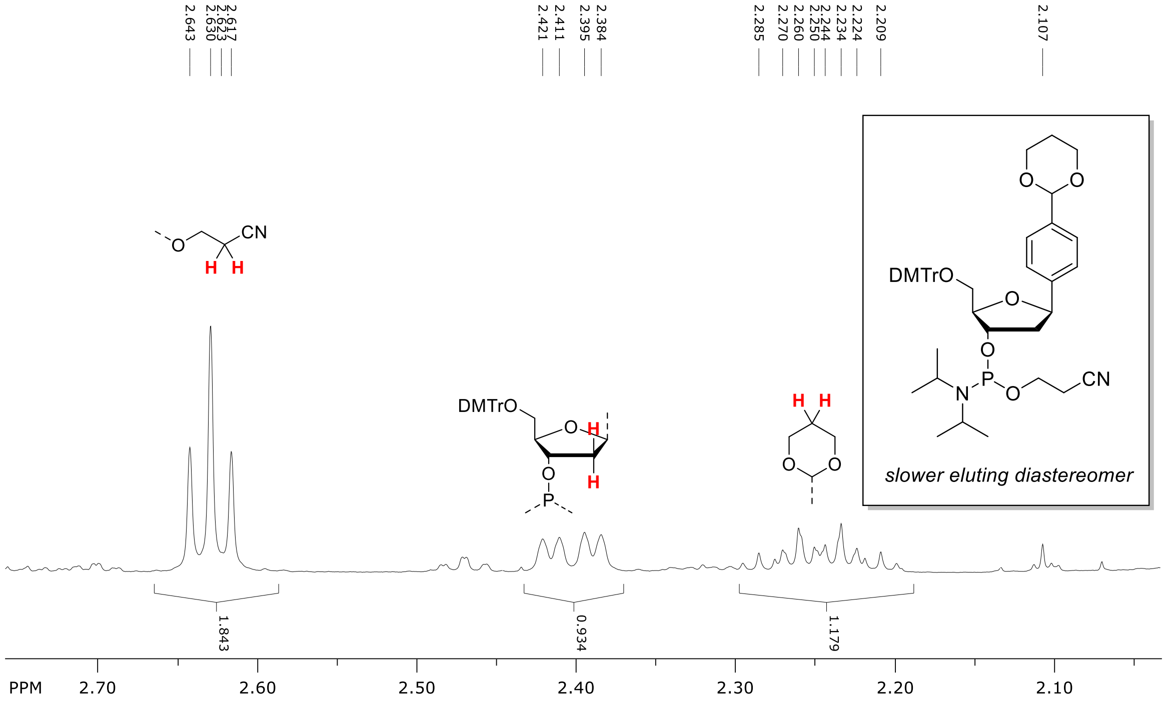
2.643 2.630 2.617
2.421 2.411 2.395 2.384
2.285 2.270 2.260 2.250 2.244 2.234 2.224 2.209
2.107
CN
H H
DMTrO
O
P
slower eluting diastereomer
1.843
0.934
1.179
PPM
2.70
2.60
2.50
2.40
2.30
2.20
2.10

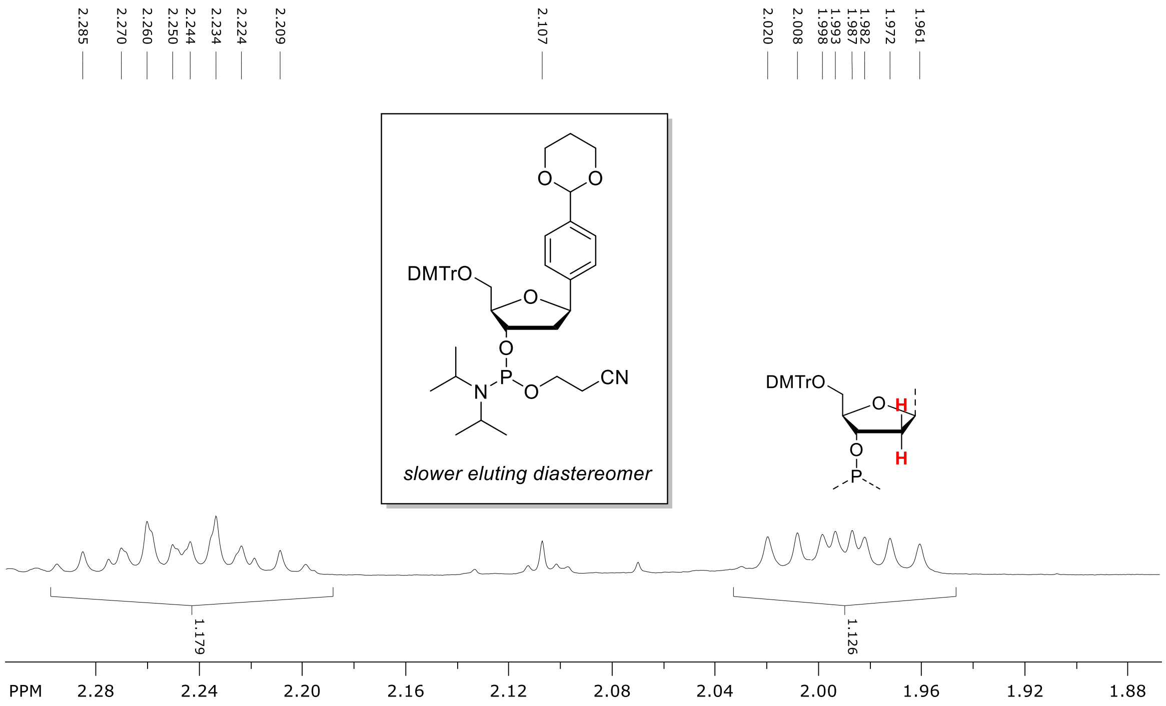

2.285
2.270
2.260
2.250
2.244
2.234
2.224
2.209
2.107
2.020
2.008
1.998
1.993
1.987
1.982
1.972
1.961
DMTrO
O
O
O
P
N
O
CN
slower eluting diastereomer
H
H
1.179
1.126
PPM 2.28 2.24 2.20 2.16 2.12 2.08 2.04 2.00 1.96 1.92 1.88


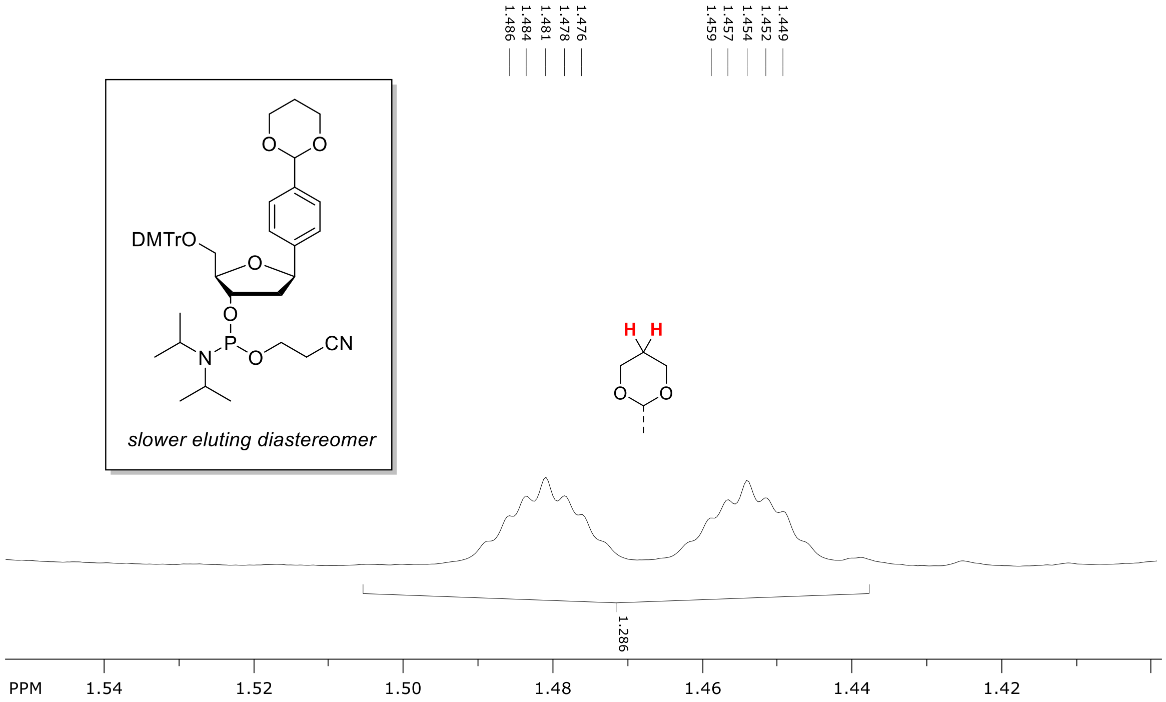

1.486
1.484
1.481
1.478
1.476
1.459
1.457
1.454
1.452
1.449
DMTrO
slower eluting diastereomer
H H
1.286
PPM 1.54 1.52 1.50 1.48 1.46 1.44 1.42

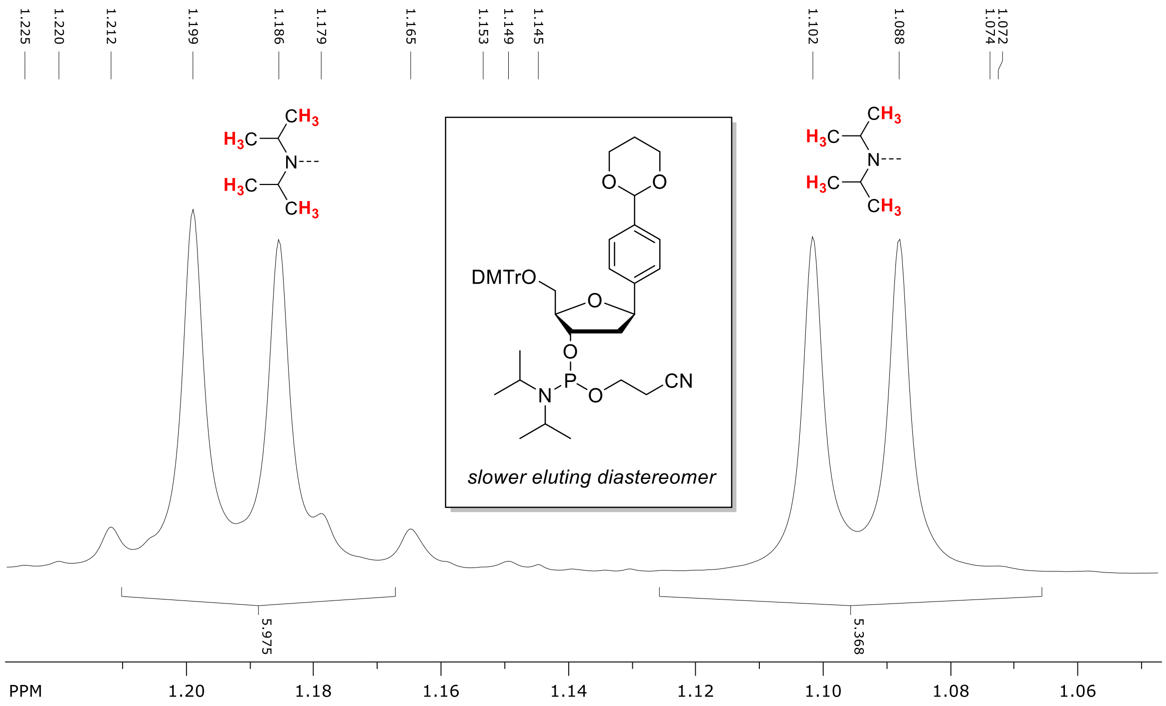

1.225
1.220
1.212
1.199
1.186
1.179
1.165
1.153
1.149
1.145
1.102
1.088
1.072
1.074
5.975
5.368
PPM
1.20
1.18
1.16
1.14
1.12
1.10
1.08
1.06
DMTrO
CN
slower eluting diastereomer


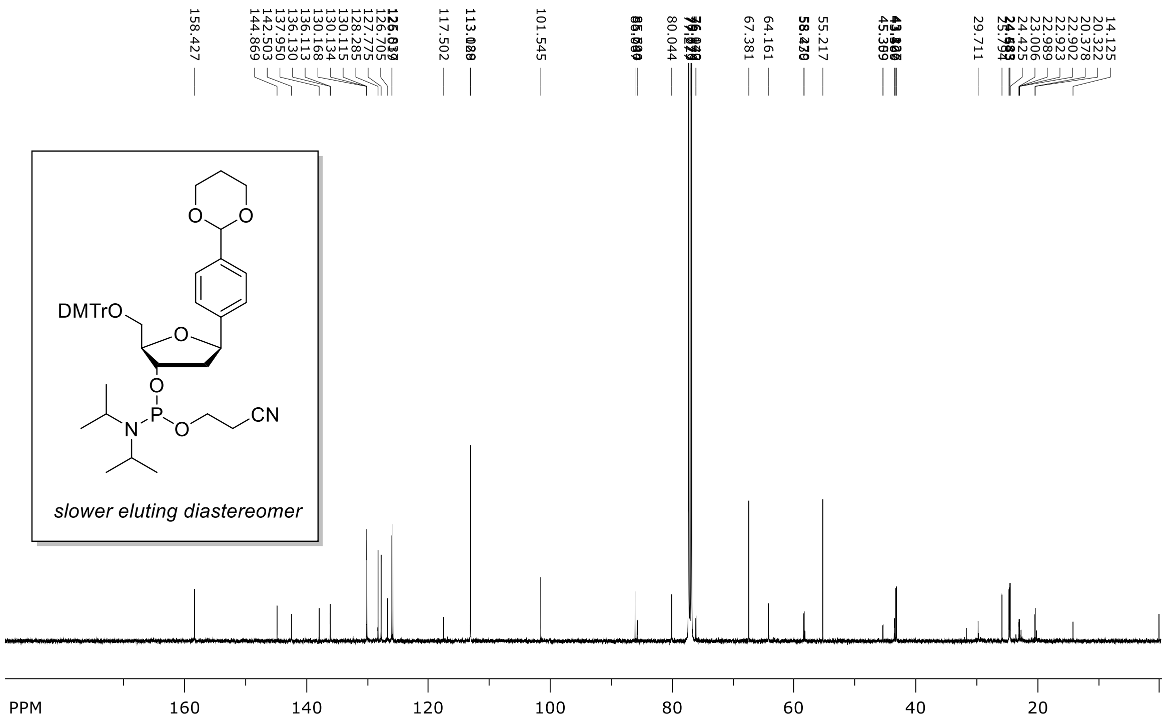

158.427
144.869
142.503
137.950
136.130
136.113
130.168
130.134
130.115
128.285
127.775
126.705
117.502
101.545
80.044
67.381
64.161
55.217
45.369
29.711
24.425
23.006
22.989
22.923
22.902
20.378
20.322
14.125
PPM
160
140
120
100
80
60
40
20
DMTrO
CN
slower eluting diastereomer

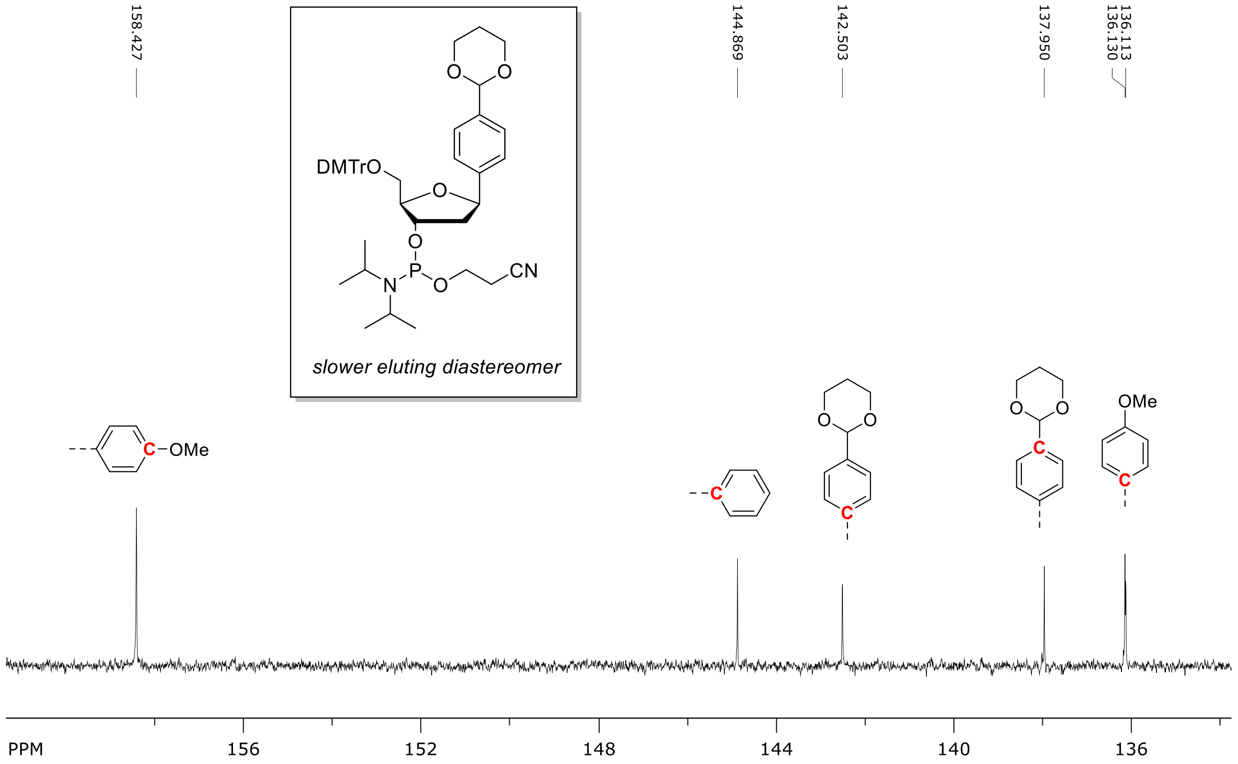
158.427
144.869
142.503
137.950
136.113
136.130
DMTrO
O
O
O
O
P
N
CN
slower eluting diastereomer
C
OMe
PPM
156
152
148
144
140
136

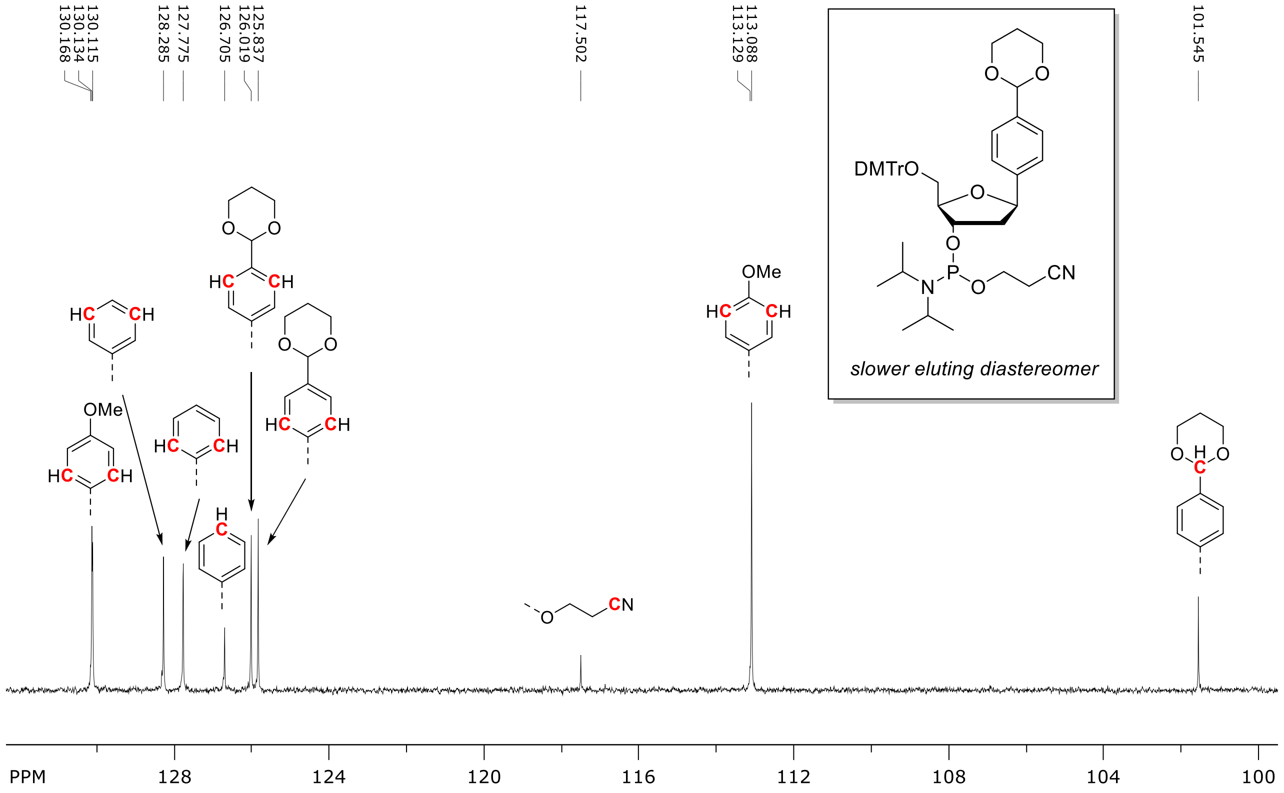
130.115
130.134
130.168
128.285
127.775
126.705
126.019
125.837
117.502
113.088
113.129
101.545
DMTrO
O
P
N
CN
slower eluting diastereomer
HC
CH
OMe
H
C
PPM
128
124
120
116
112
108
104
100

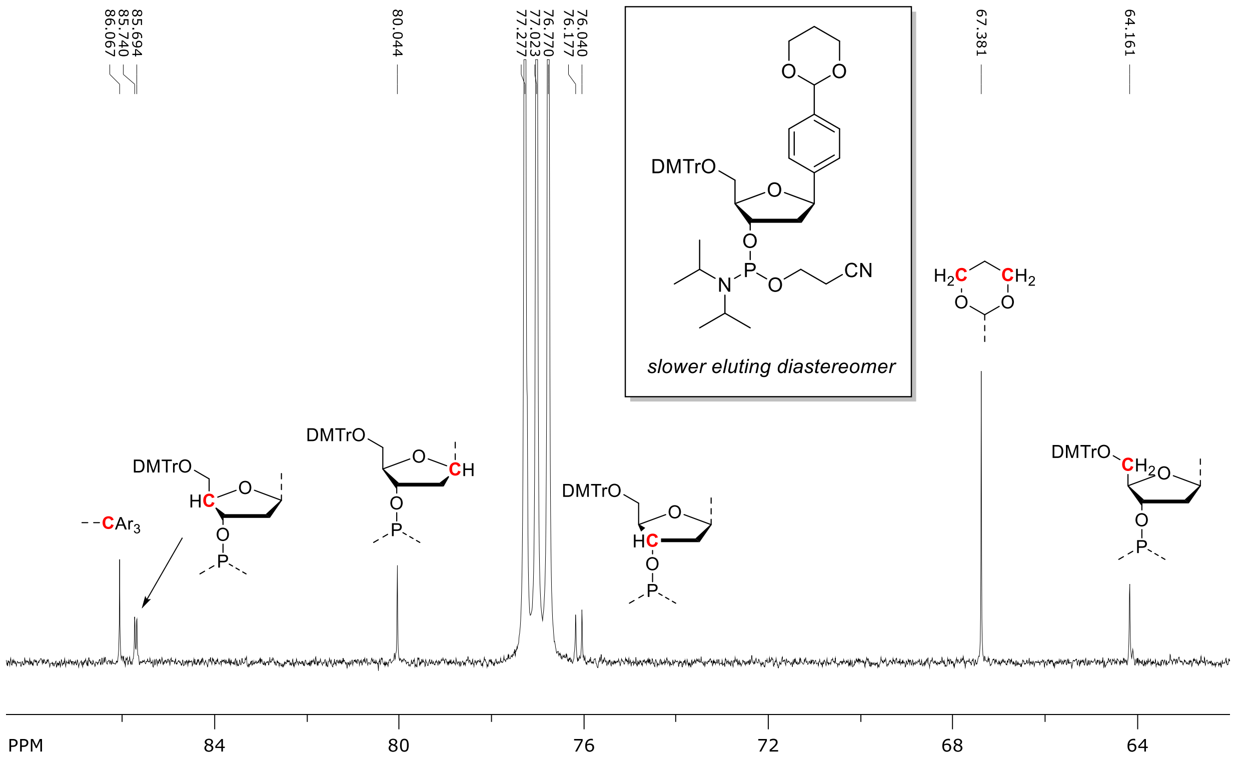
85.694
85.740
86.067
80.044
77.277
77.023
76.770
76.177
76.040
67.381
64.161
DMTrO
slower eluting diastereomer
CAr3
PPM
84
80
76
72
68
64

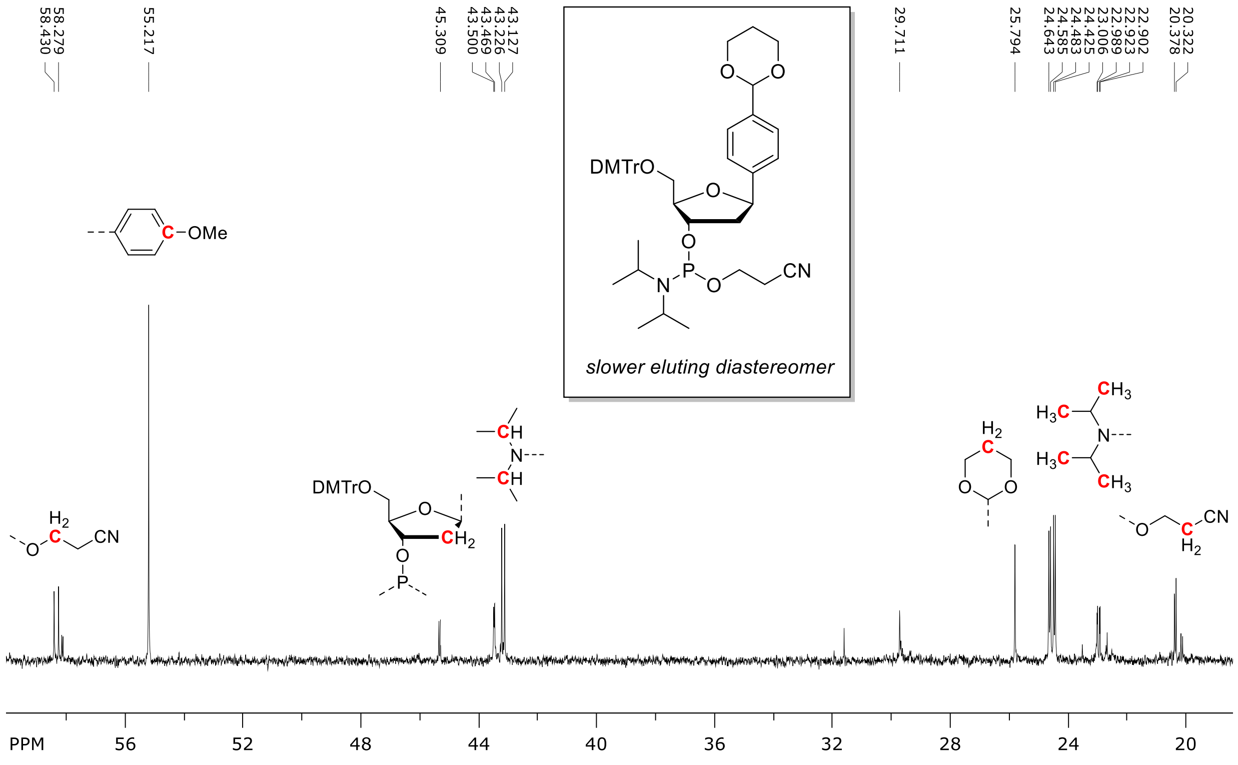
58.279
58.430
55.217
45.309
43.500
43.469
43.226
43.127
29.711
25.794
24.643
24.585
24.483
24.425
23.006
22.989
22.923
22.902
20.322
20.378
DMTrO
slower eluting diastereomer
OMe
CN
PPM
56
52
48
44
40
36
32
28
24
20

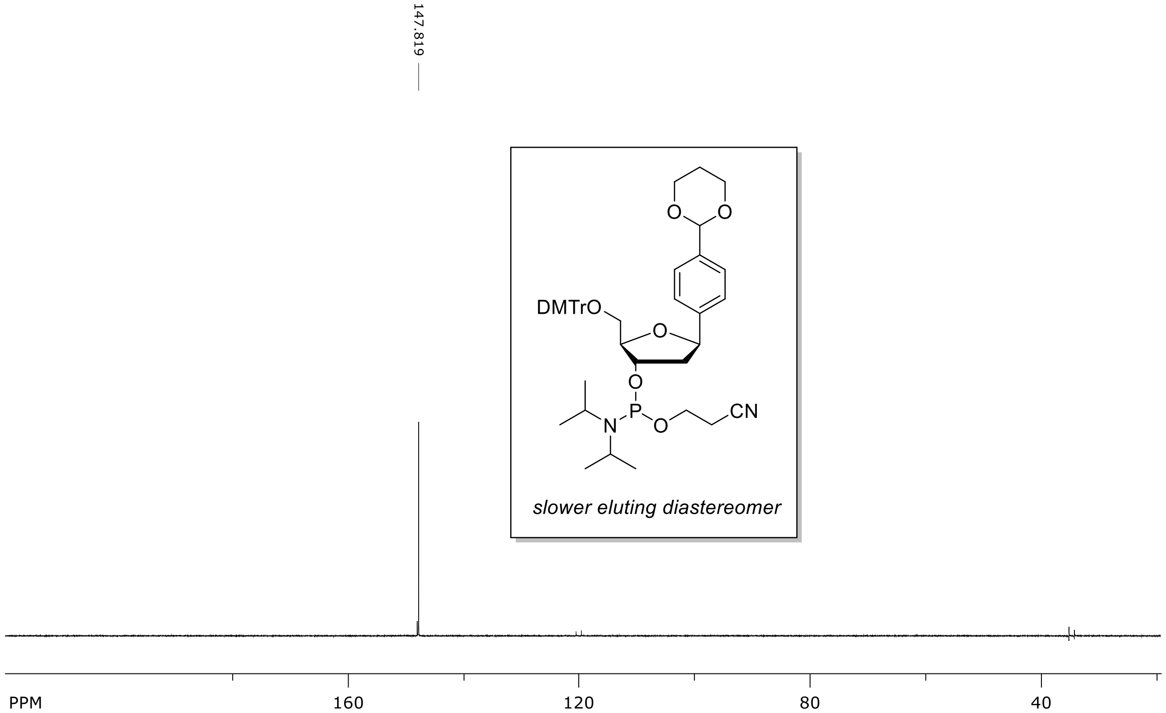
147.819
DMTrO
O
CN
P
N
slower eluting diastereomer
PPM
160
120
80
40

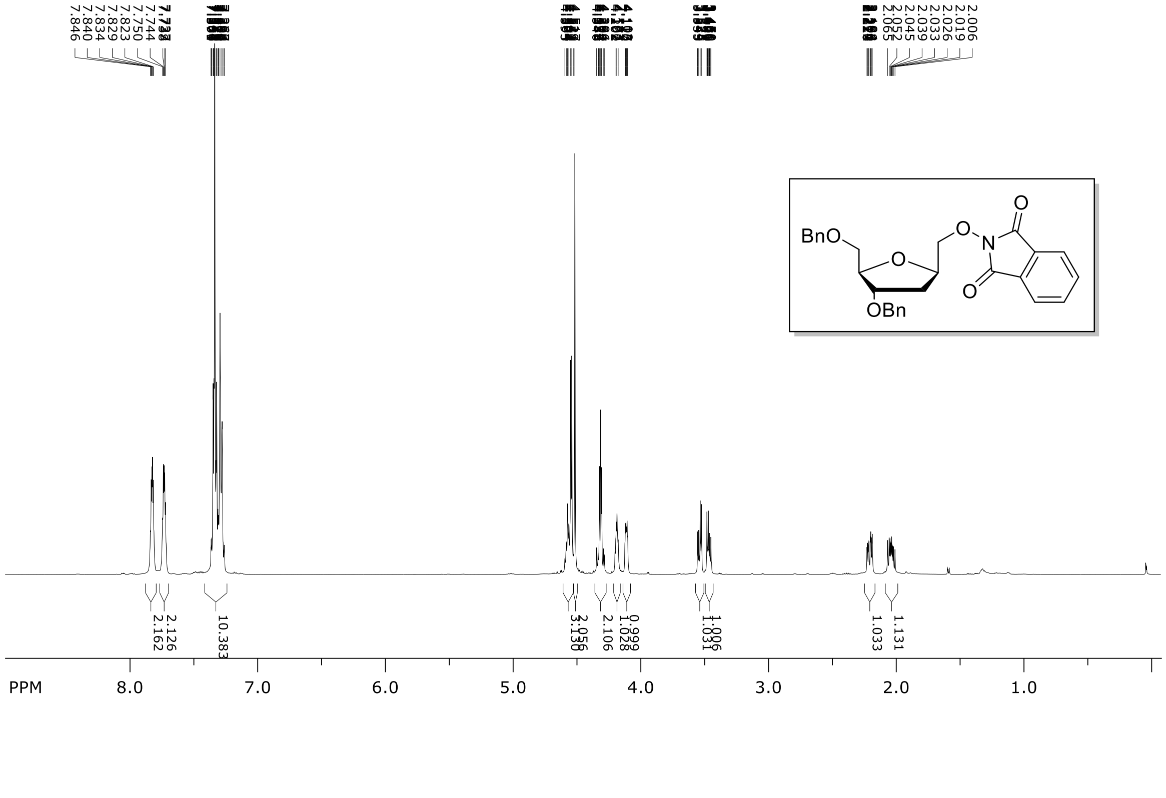
7.846
7.840
7.834
7.829
7.823
7.750
7.744
2.065
2.045
2.039
2.033
2.026
2.019
2.006
BnO
O
N
OBn
2.162
2.126
10.383
2.106
0.999
1.028
1.006
1.031
1.033
1.131
PPM
8.0
7.0
6.0
5.0
4.0
3.0
2.0
1.0

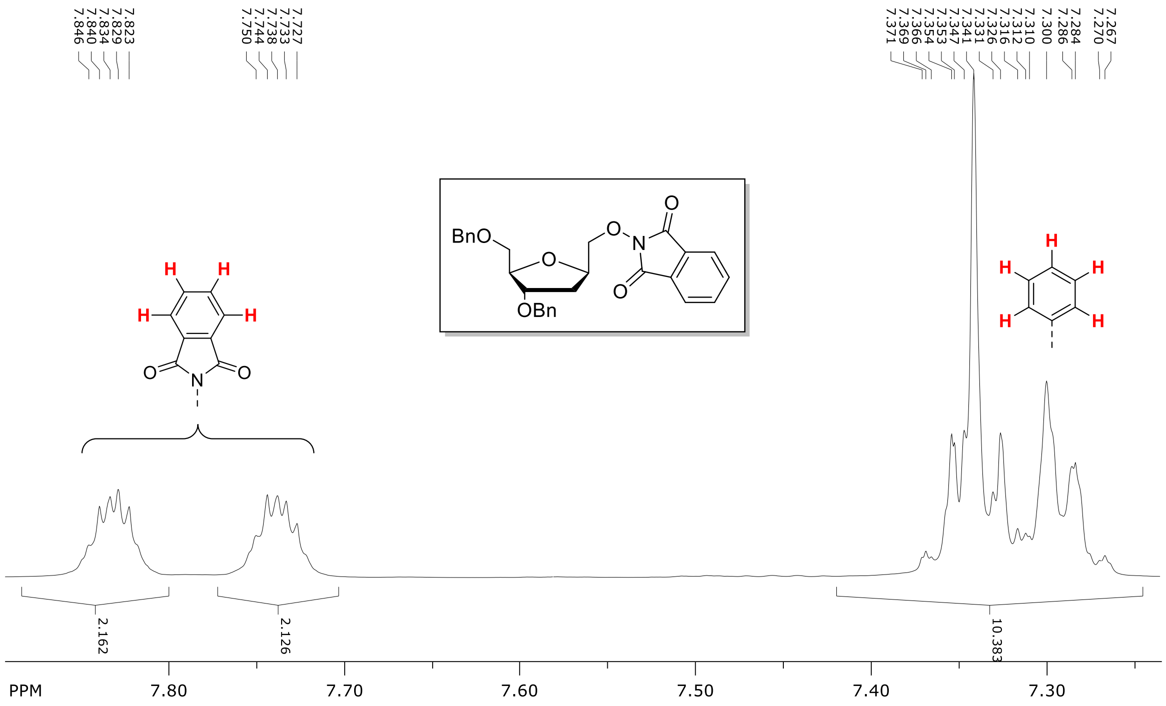

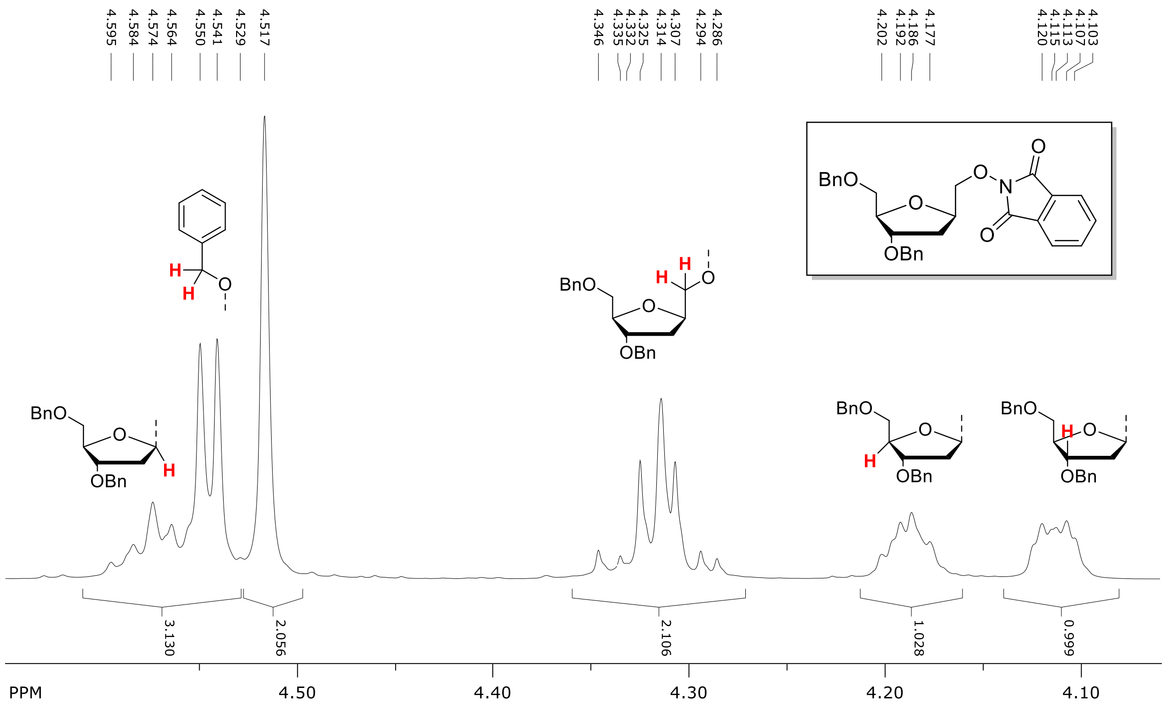

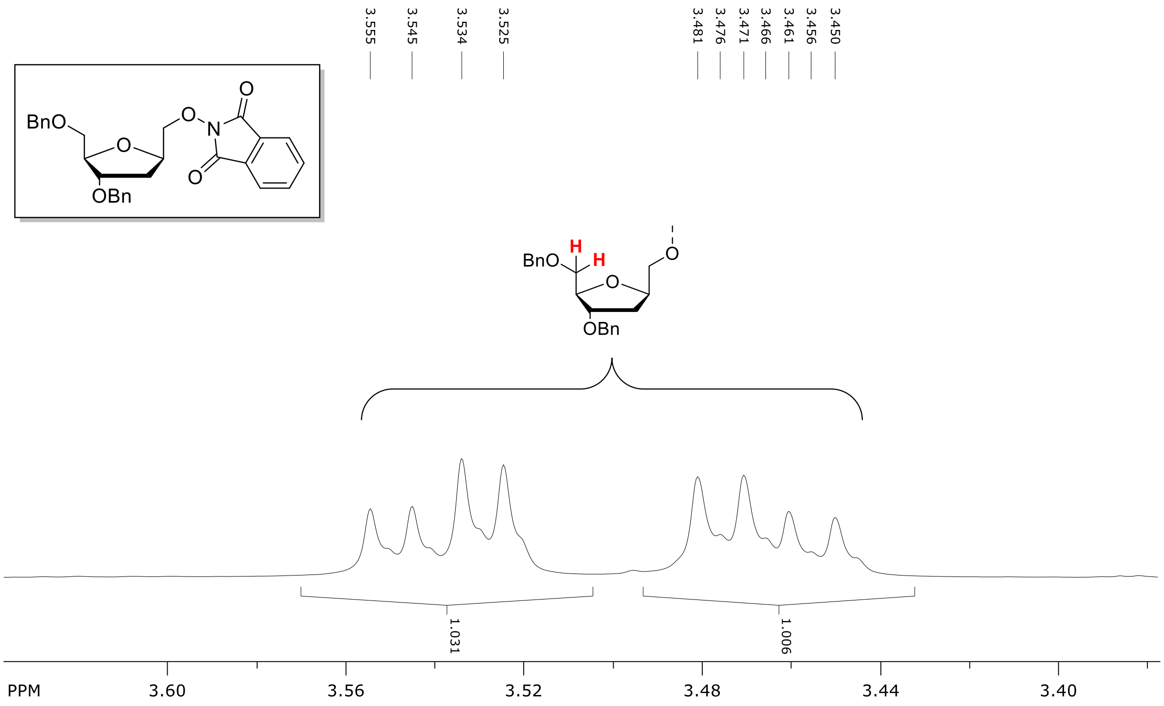
3.555
3.545
3.534
3.525
3.481
3.476
3.471
3.466
3.461
3.456
3.450
BnO
O
N
OBn
BnO
H
H
OBn
1.031
1.006
PPM
3.60
3.56
3.52
3.48
3.44
3.40

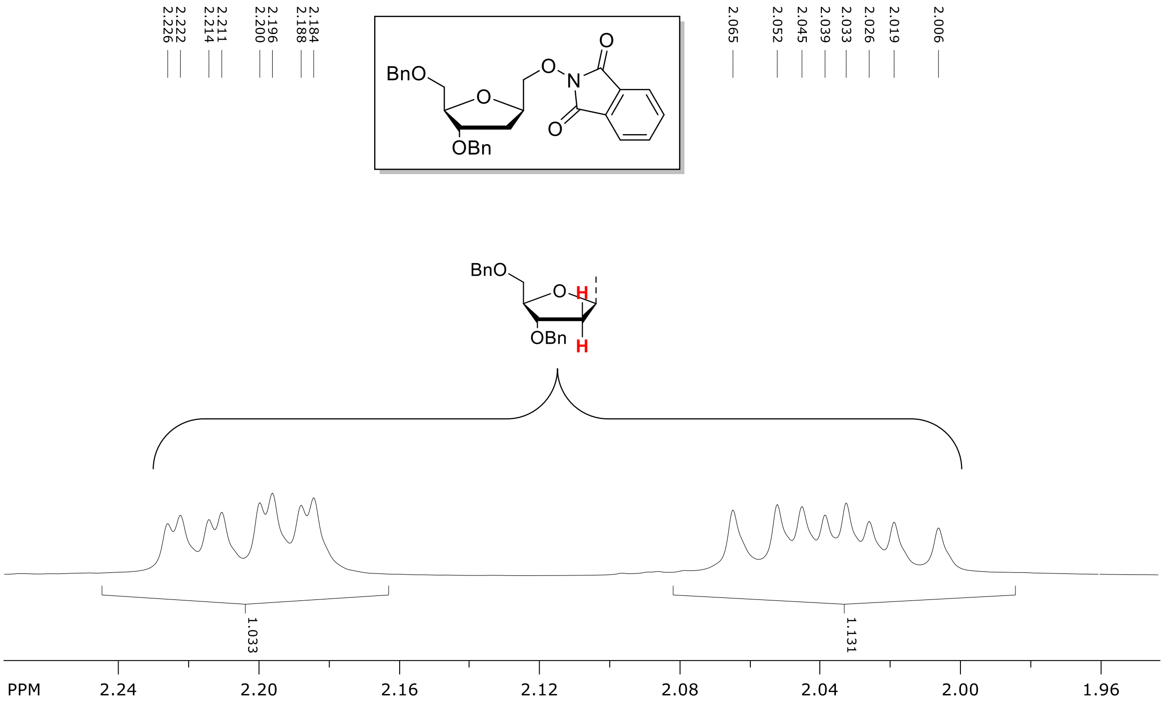
2.226
2.222
2.214
2.211
2.200
2.196
2.188
2.184
2.065
2.052
2.045
2.039
2.033
2.026
2.019
2.006
BnO
O
N
OBn
BnO
H
OBn
H
1.033
1.131
PPM
2.24
2.20
2.16
2.12
2.08
2.04
2.00
1.96

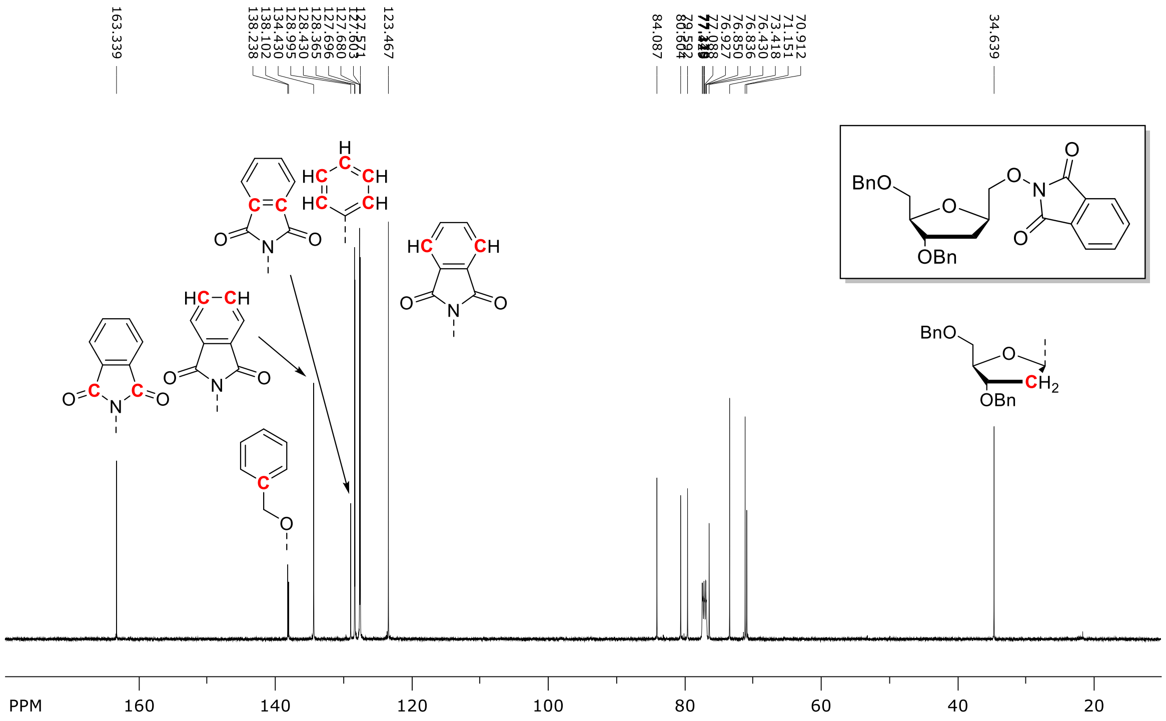
163.339
138.238
138.102
134.430
128.995
128.430
128.365
127.696
127.680
127.571
127.503
123.467
84.087
80.604
79.592
76.927
76.850
76.836
76.430
73.418
71.151
70.912
34.639
BnO
OBn
PPM
160
140
120
100
80
60
40
20

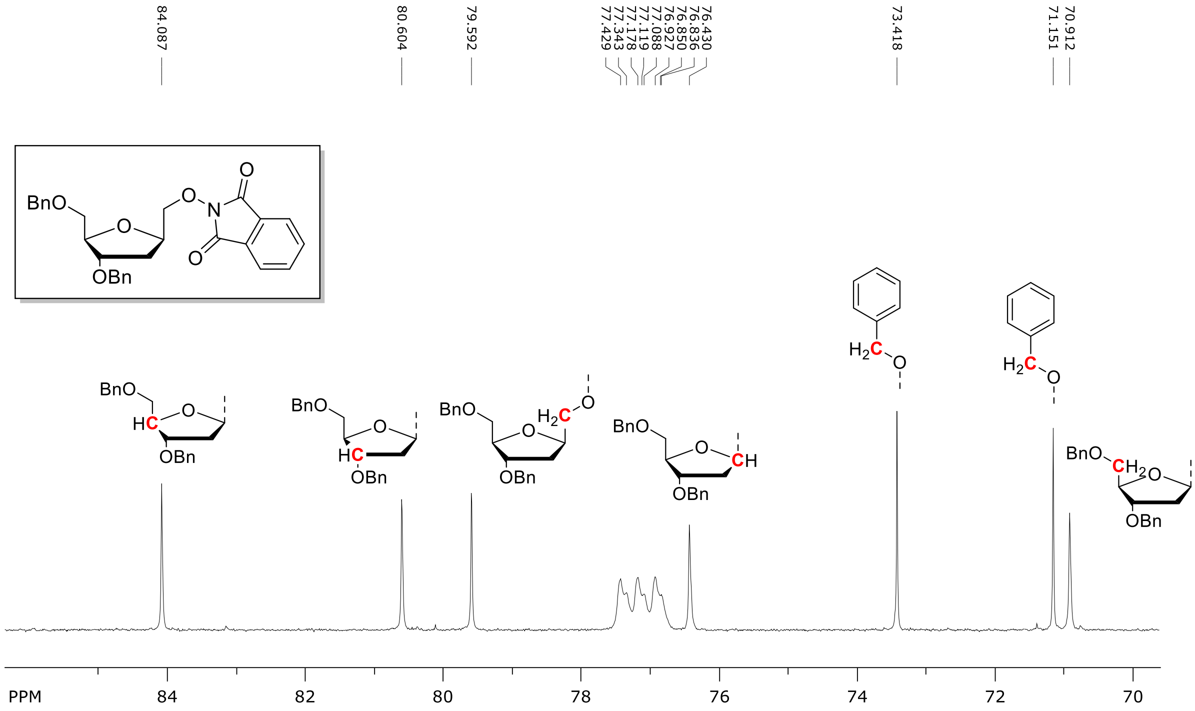
84.087
80.604
79.592
77.429
77.343
77.178
77.119
77.088
76.927
76.850
76.836
76.430
73.418
71.151
70.912
BnO
OBn
PPM
84
82
80
78
76
74
72
70

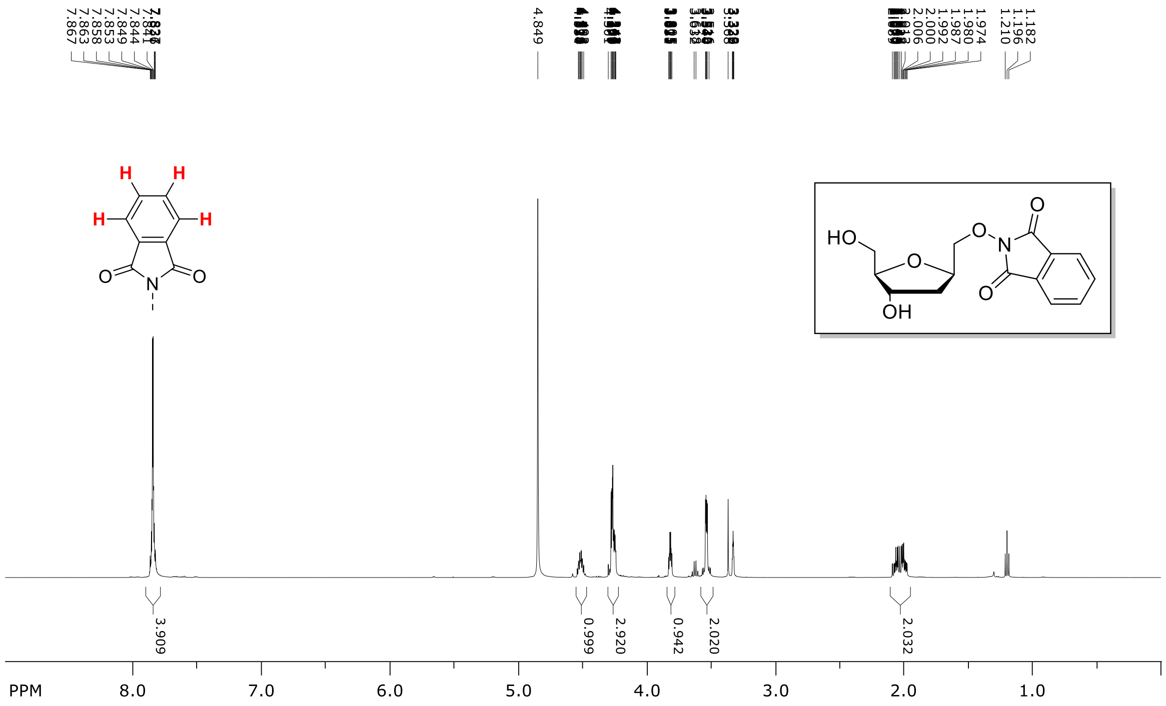

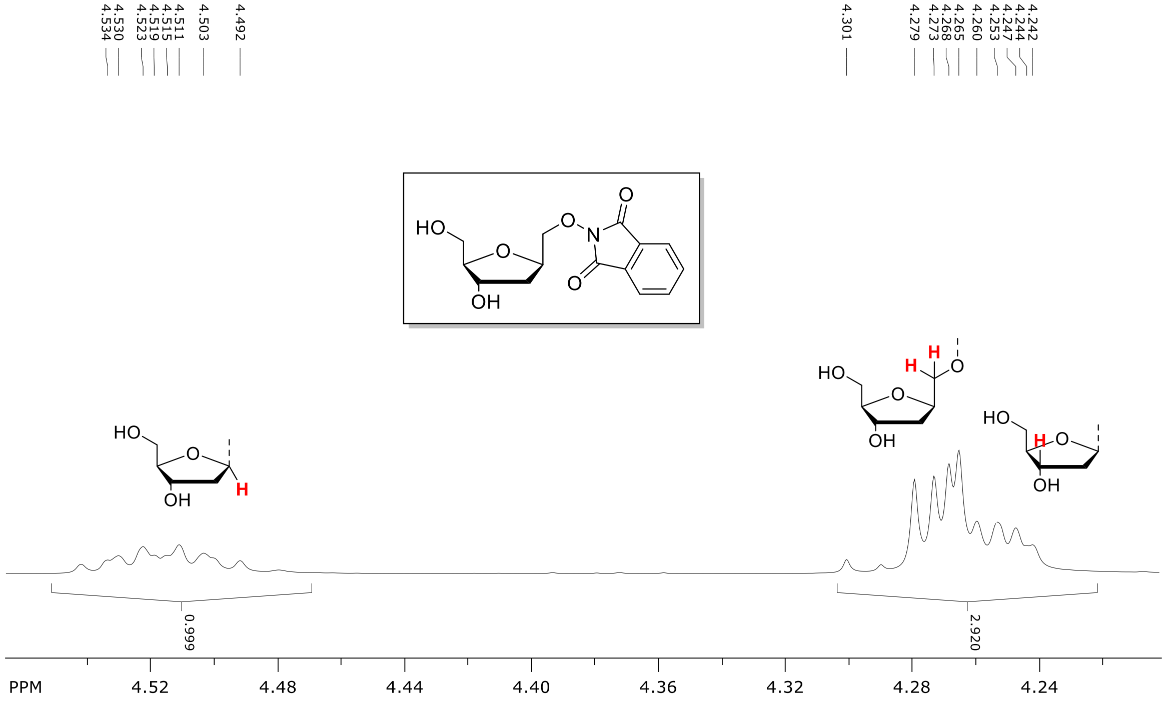

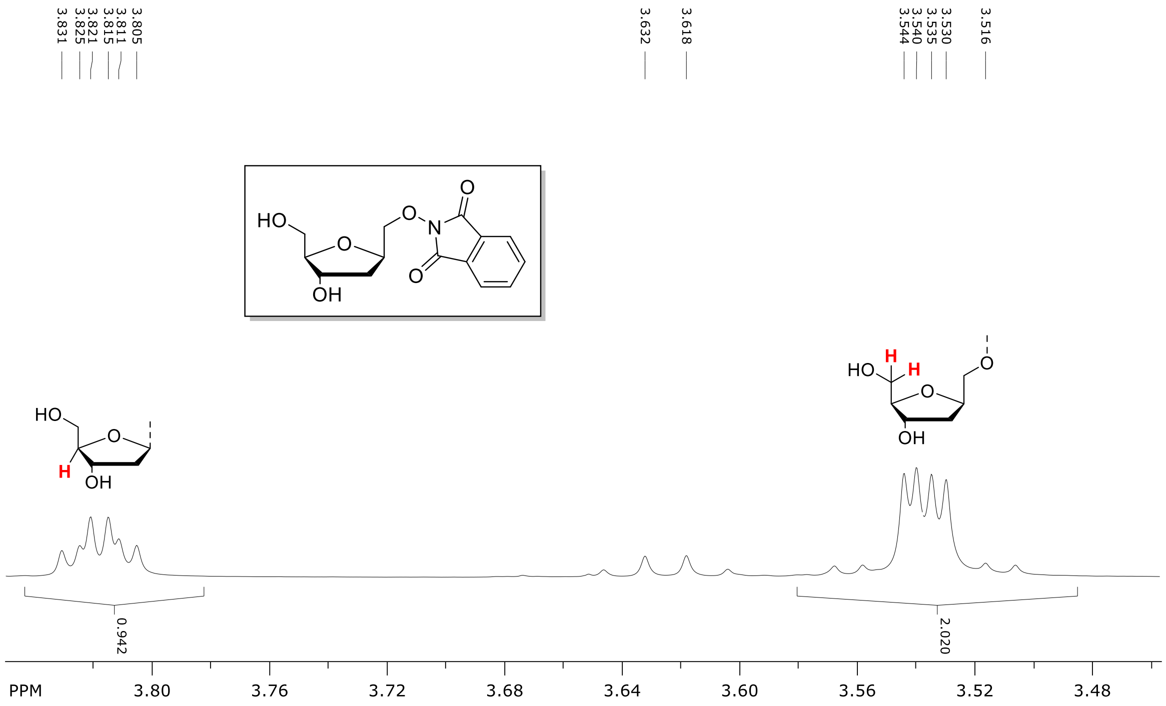
3.831
3.825
3.821
3.815
3.811
3.805
3.632
3.618
3.544
3.540
3.535
3.530
3.516
HO
OH
O
N
0.942
2.020
PPM
3.80
3.76
3.72
3.68
3.64
3.60
3.56
3.52
3.48

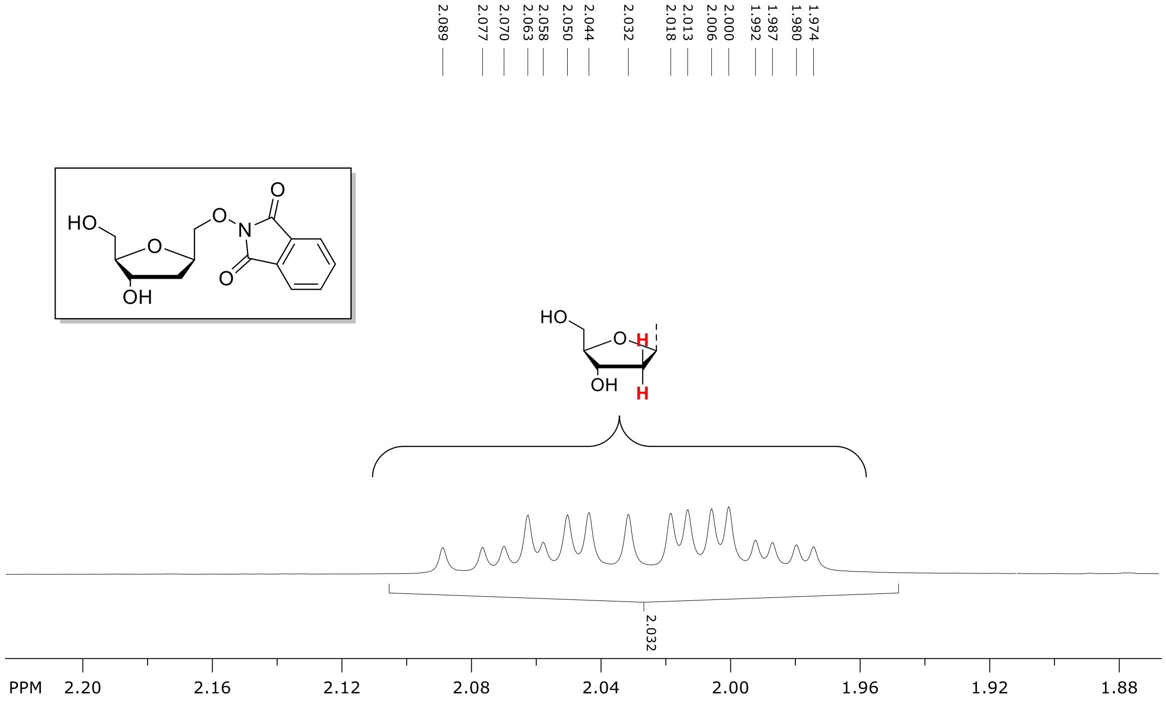
2.089
2.077
2.070
2.063
2.058
2.050
2.044
2.032
2.018
2.013
2.006
2.000
1.992
1.987
1.980
1.974
HO
OH
O
N
2.032
PPM
2.20
2.16
2.12
2.08
2.04
2.00
1.96
1.92
1.88

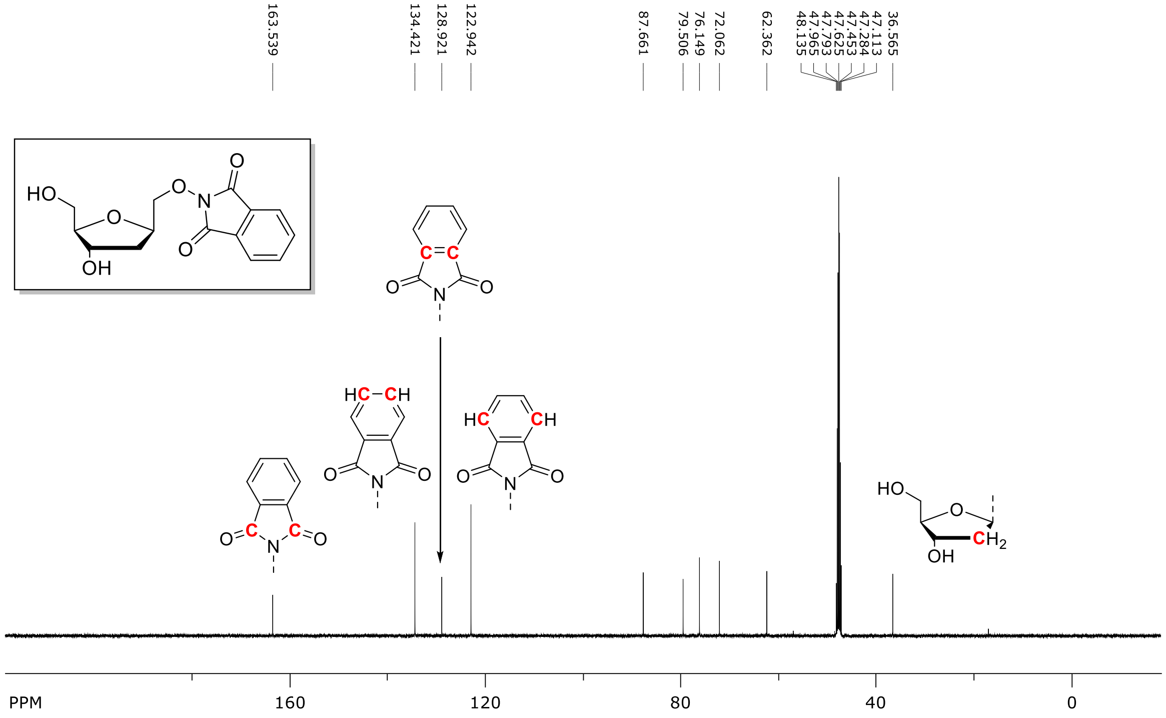
163.539
134.421
128.921
122.942
87.661
79.506
76.149
72.062
62.362
48.135
47.965
47.793
47.625
47.453
47.284
47.113
36.565
HO
O
N
O
O
OH
HC–CH
HC
CH
CH2
PPM
160
120
80
40
0

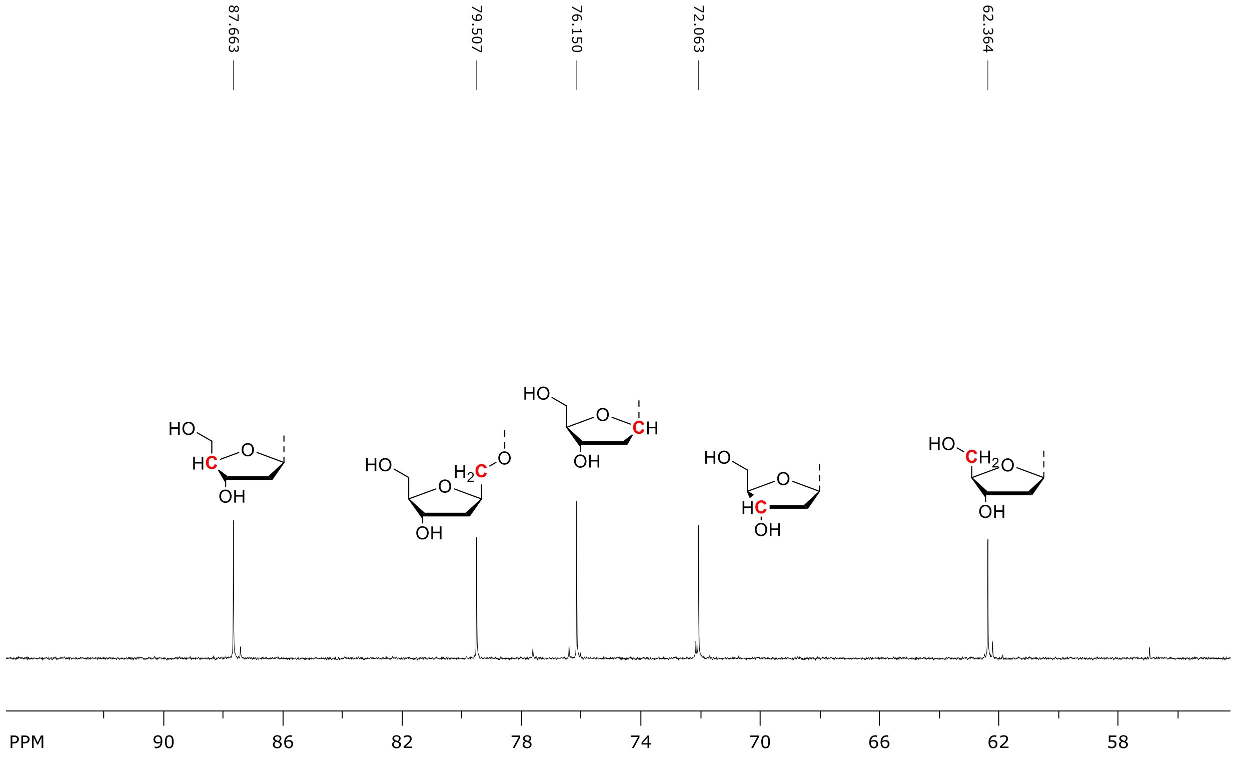
87.663
79.507
76.150
72.063
62.364
HO
HC
O
OH
H2C
CH
HC
CH2
PPM
90
86
82
78
74
70
66
62
58

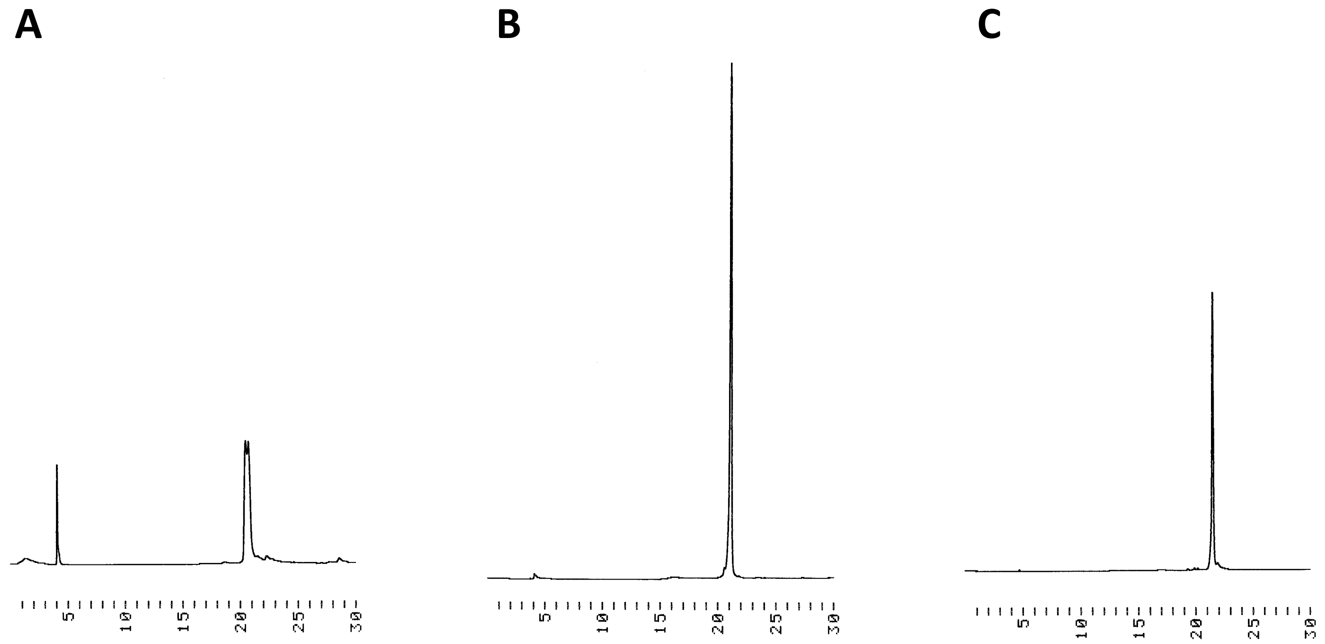


**Figure S1.** HPLC traces of A) crude oligonucleotide **ON1x** and purified B) faster- and C) slower-eluting isomers of oligonucleotide **ON1x**; Hypersil ODS C18 column (250 × 4.6 mm, 5 μm); flow rate = 1.0 mL $min^{-1}$; linear gradient (5-30% over 30 min) of MeCN in 50 mM aqueous triethylammonium acetate.

# Generic Display Report

| | | | |
|---|---|---|---|
| **Analysis Info** | | Acquisition Date | 10/22/2019 8:21:19 AM |
| Analysis Name | D:\Data\Madhuri Hande\221019-MH-Oligo-3-OH-Benzaldehyde-2peaks.d | | |
| Method | oligo.m | Operator | Bruker07 |
| Sample Name | 221019-MH-Oligo-3-OH-Benzaldehyde-2peaks | Instrument | micrOTOF-Q |
| Comment | | | |

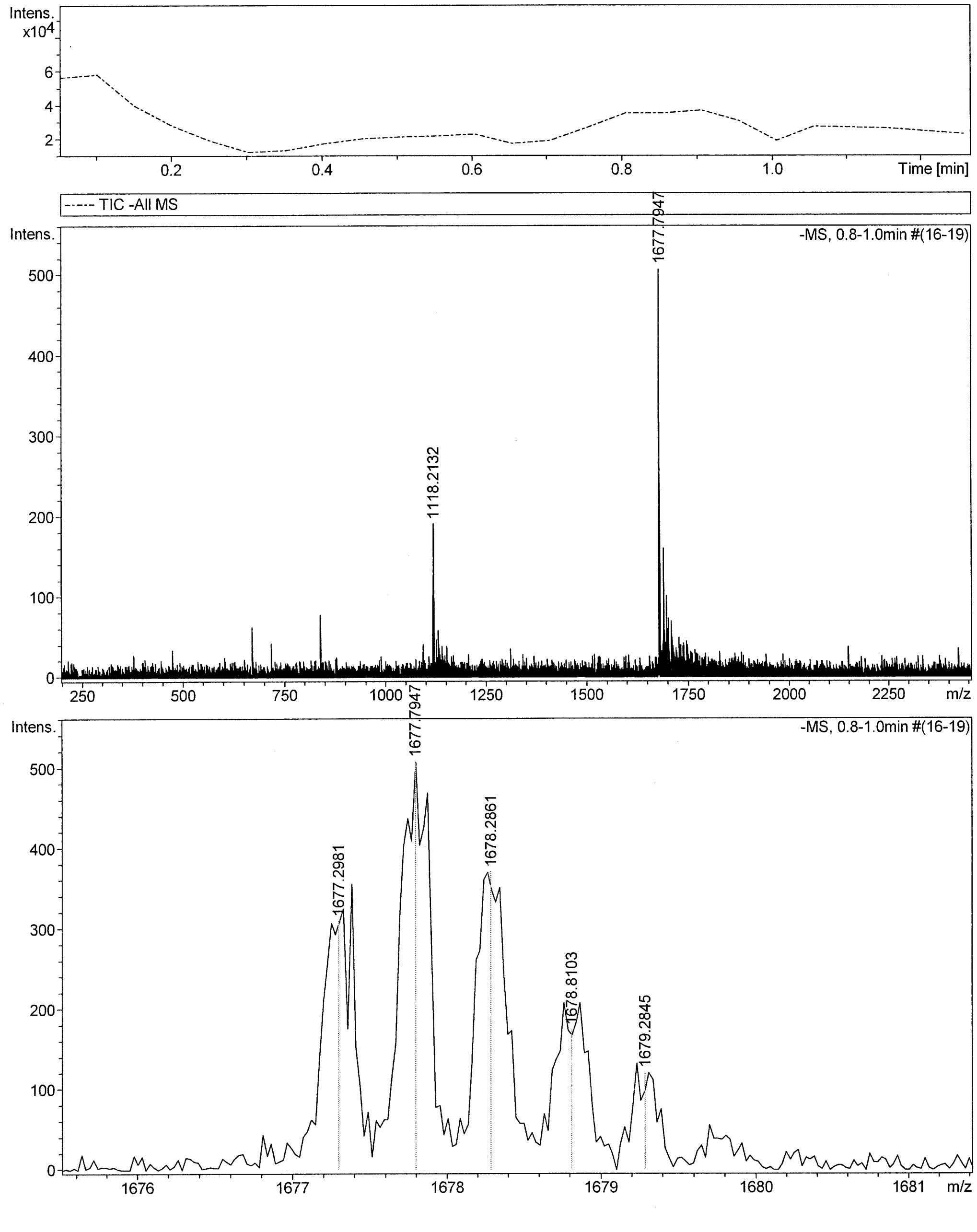


Bruker Compass DataAnalysis 4.0 printed: 10/22/2019 8:27:57 AM Page 1 of 1

**Figure S2.** Mass spectrum of the faster-eluting isomer of oligonucleotide **ON1x**.

# Generic Display Report

**Analysis Info**

| | | | |
|---|---|---|---|
| Analysis Name | D:\Data\Madhuri Hande\06022020-MH-ON-3-OH-Bz-(2).d | Acquisition Date | 2/6/2020 11:08:37 AM |
| Method | oligometodi.m | Operator | Bruker07 |
| Sample Name | 06022020-MH-ON-3-OH-Bz-(2) | Instrument | micrOTOF-Q |
| Comment | | | |

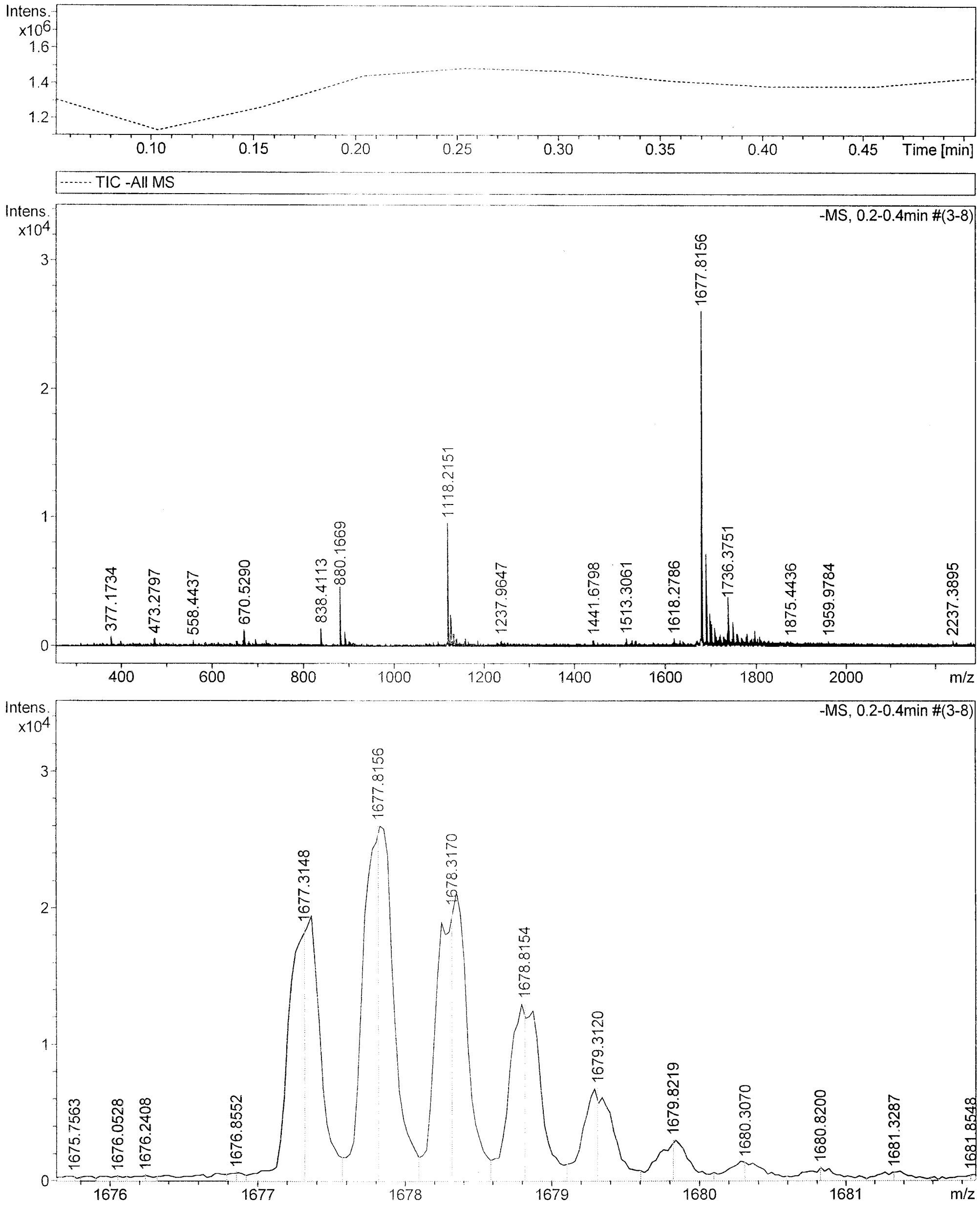


Bruker Compass DataAnalysis 4.0 printed: 2/6/2020 12:12:15 PM Page 1 of 1

**Figure S3.** Mass spectrum of the slower-eluting isomer of oligonucleotide **ON1x**.

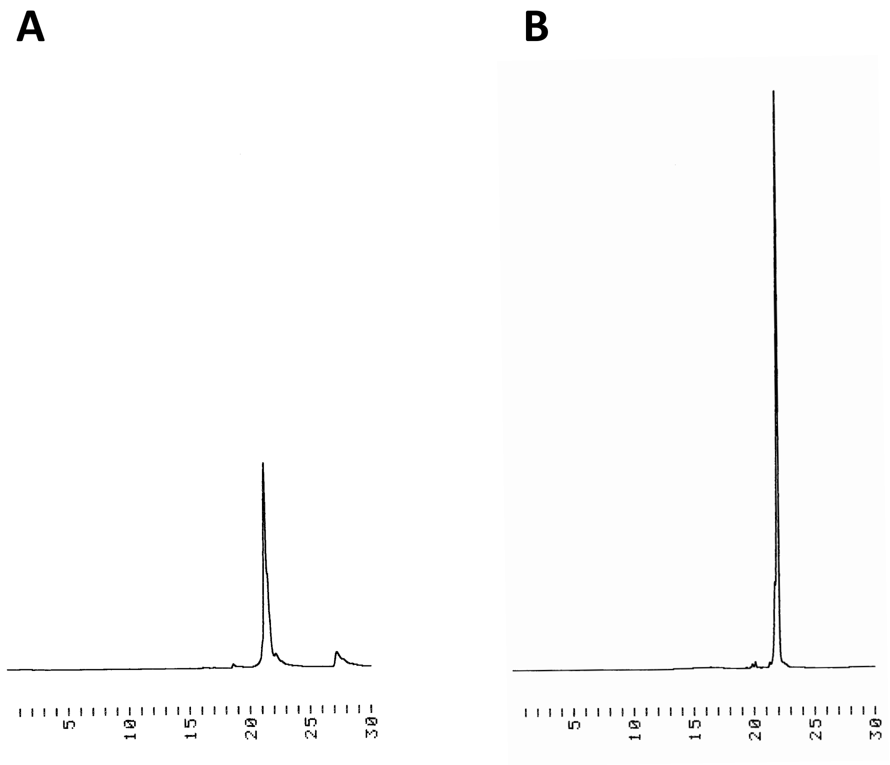


**Figure S4.** HPLC traces of A) crude and B) purified oligonucleotide **ON1y**; Hypersil ODS C18 column (250 × 4.6 mm, 5 μm); flow rate = 1.0 mL $min^{-1}$; linear gradient (5-30% over 30 min) of MeCN in 50 mM aqueous triethylammonium acetate.

## Generic Display Report

| | | | |
|---|---|---|---|
| **Analysis Info** | | Acquisition Date | 10/22/2019 8:12:56 AM |
| Analysis Name | D:\Data\Madhuri Hande\221019-MH-Oligo-Benzaldehyde-Pure.d | | |
| Method | oligo.m | Operator | Bruker07 |
| Sample Name | 221019-MH-Oligo-Benzaldehyde-Pure | Instrument | micrOTOF-Q |
| Comment | | | |

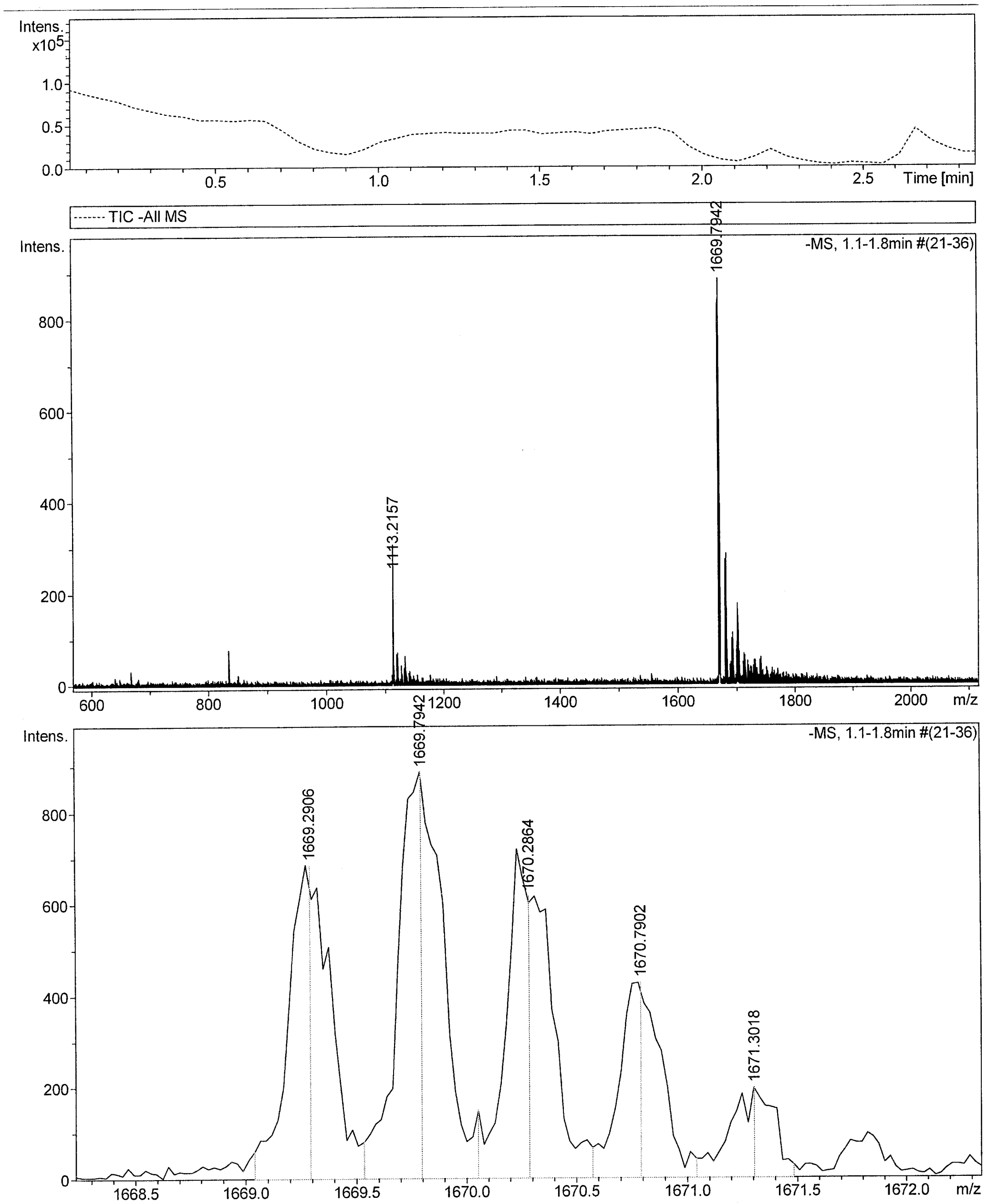


Bruker Compass DataAnalysis 4.0 printed: 10/22/2019 8:26:42 AM Page 1 of 1

**Figure S5.** Mass spectrum of oligonucleotide **ON1y**.

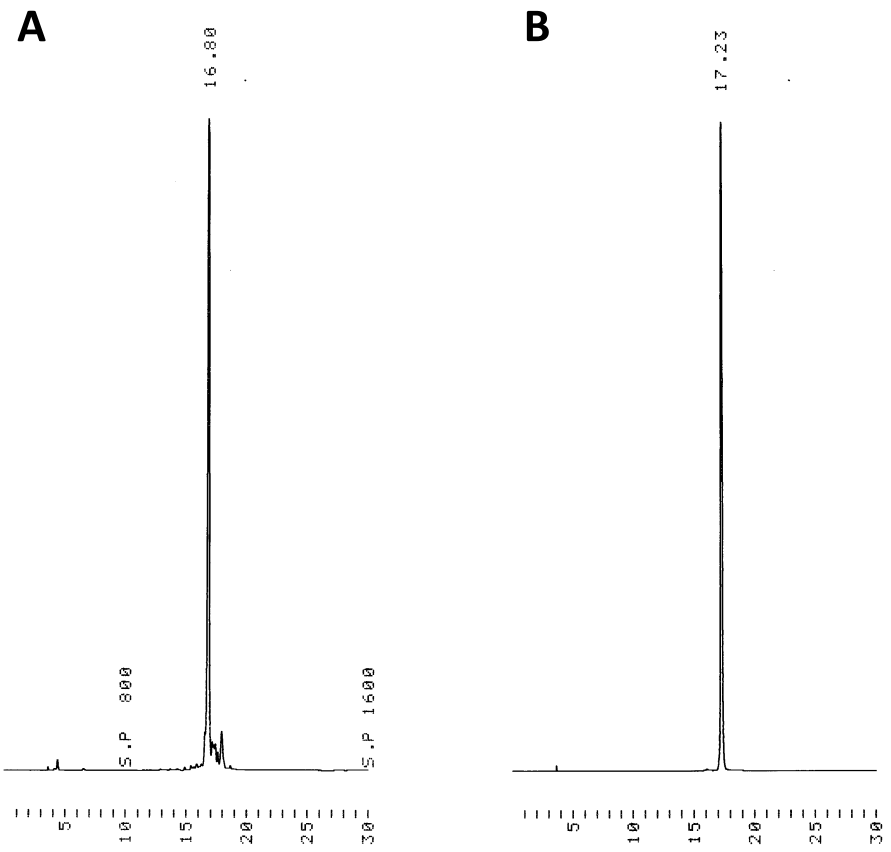


**Figure S6.** HPLC traces of A) crude and B) purified oligonucleotide **ON1b**; Hypersil ODS C18 column (250 × 4.6 mm, 5 μm); flow rate = 1.0 mL min$^{-1}$; linear gradient (5-30% over 30 min) of MeCN in 50 mM aqueous triethylammonium acetate.

# Generic Display Report

| | | | |
|---|---|---|---|
| **Analysis Info** | | Acquisition Date | 11/7/2017 4:45:29 PM |
| Analysis Name | D:\Data\SajalTuomas\SKMTL-NB2-P-68-11-mer-071117.d | | |
| Method | oligo.m | Operator | Bruker07 |
| Sample Name | SKMTL-NB2-P-68-11-mer-071117 | Instrument | micrOTOF-Q |
| Comment | | | |

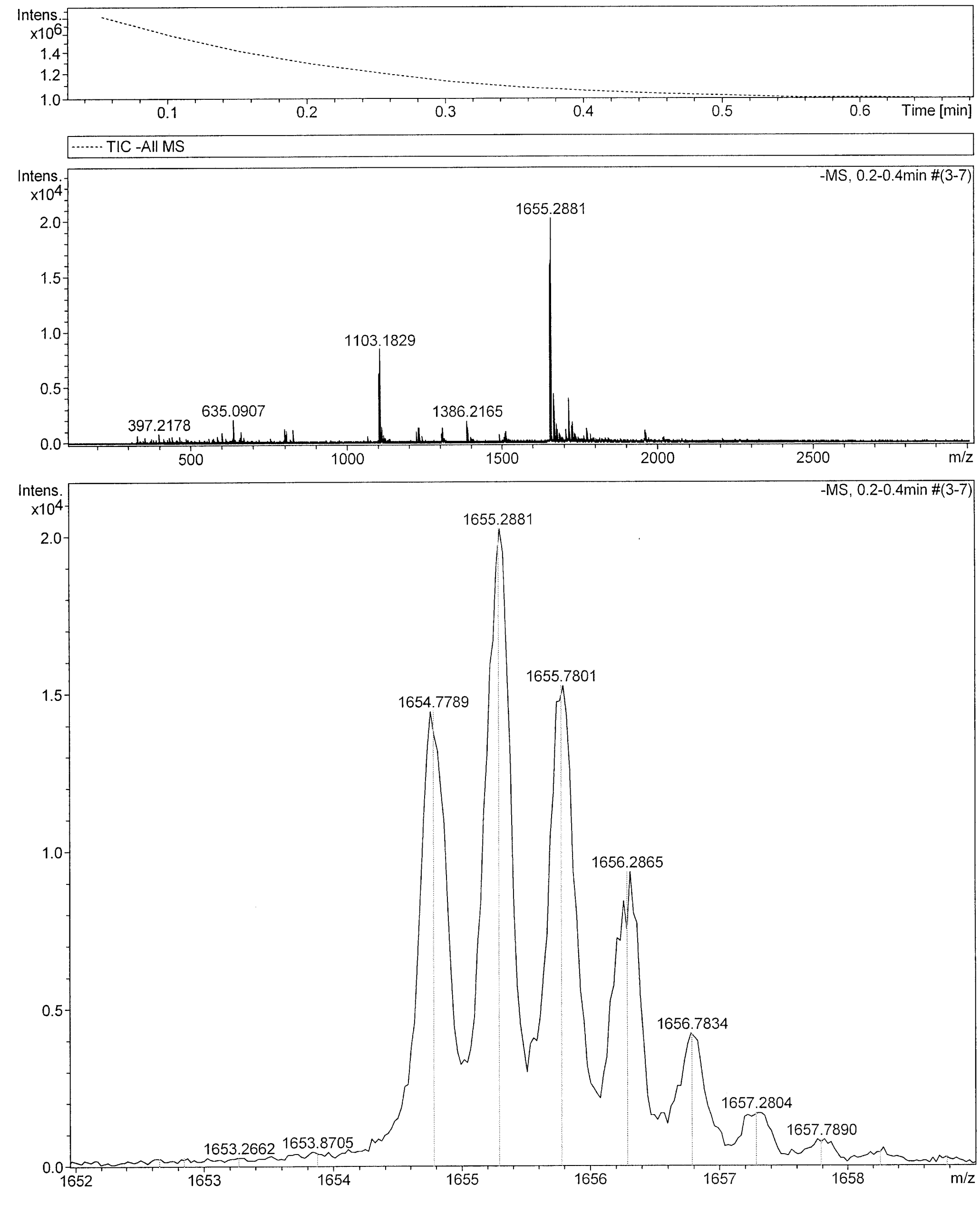


Bruker Compass DataAnalysis 4.0 printed: 11/7/2017 5:09:22 PM Page 1 of 1

**Figure S7.** Mass spectrum of oligonucleotide **ON1b**.

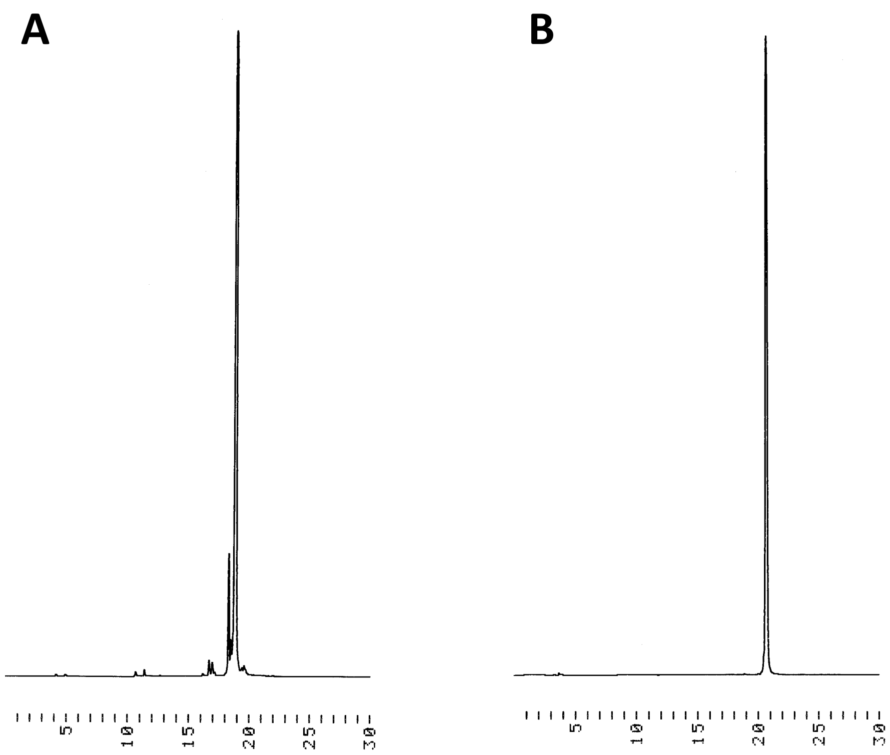


**Figure S8.** HPLC traces of A) crude and B) purified oligonucleotide **ON1z**; Hypersil ODS C18 column (250 × 4.6 mm, 5 μm); flow rate = 1.0 mL $min^{-1}$; linear gradient (5-30% over 30 min) of MeCN in 50 mM aqueous triethylammonium acetate.

# Generic Display Report

| **Analysis Info** | | Acquisition Date | 11/24/2017 2:15:02 PM |
|---|---|---|---|
| Analysis Name | D:\Data\SajalTuomas\SKMTL-NB2-P-77 at rt for 24h bulk_241117.d | | |
| Method . | oligo.m | Operator | Bruker07 |
| Sample Name | SKMTL-NB2-P-77 at rt for 24h bulk_241117 | Instrument | micrOTOF-Q |
| Comment | | | |

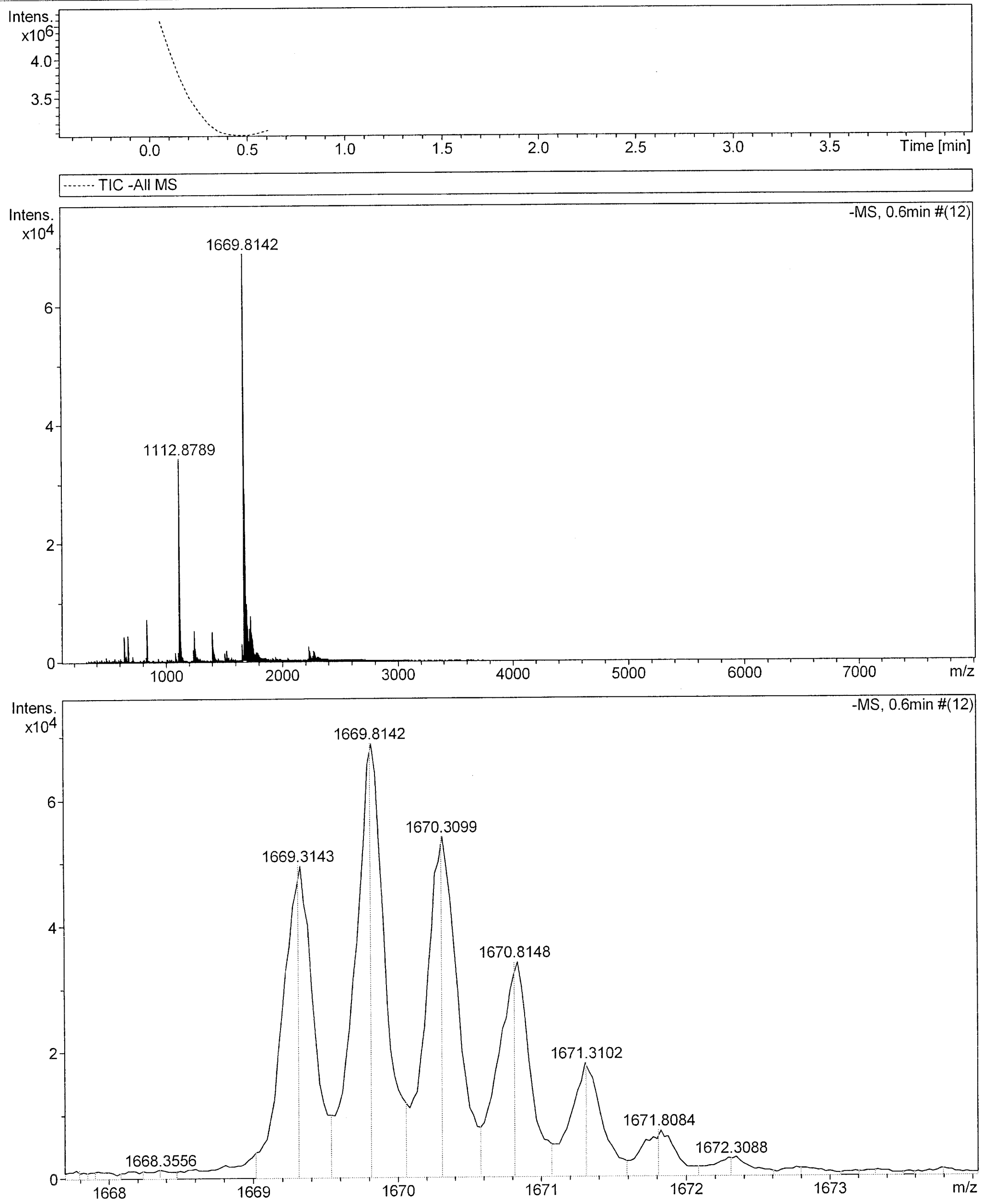


Bruker Compass DataAnalysis 4.0 printed: 11/24/2017 2:24:43 PM Page 1 of 1

**Figure S9.** Mass spectrum of oligonucleotide **ON1z**.

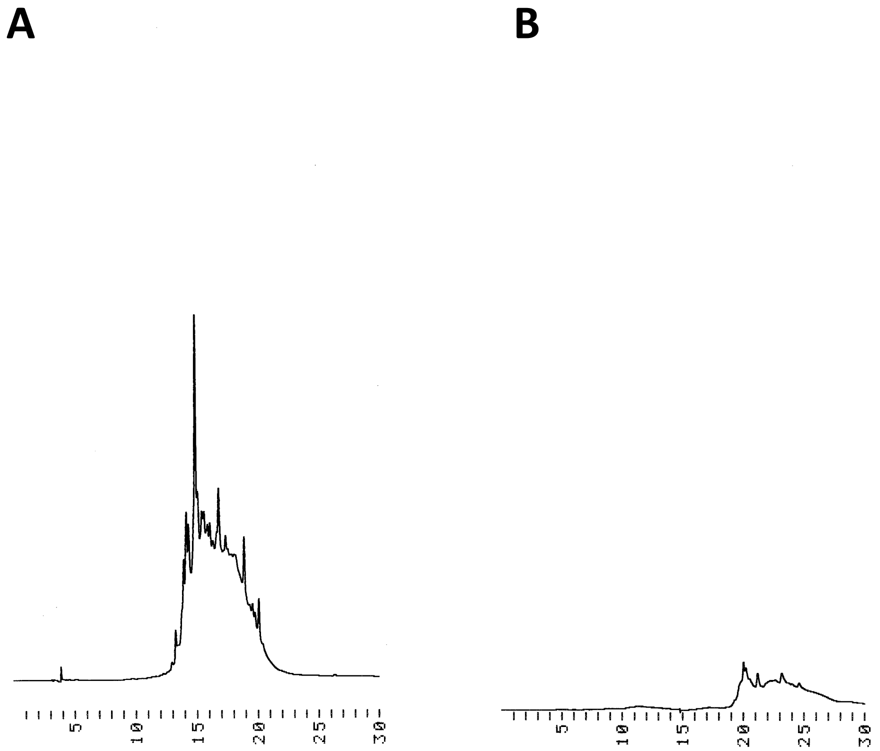


**Figure S10.** HPLC traces of A) crude and B) purified oligonucleotide **ON1z-Pd**; Hypersil ODS C18 column (250 × 4.6 mm, 5 μm); flow rate = 1.0 mL min$^{-1}$; linear gradient (5-30% over 30 min) of MeCN in 50 mM aqueous triethylammonium acetate.

## Generic Display Report

| | | | |
|---|---|---|---|
| **Analysis Info** | | Acquisition Date | 2/23/2018 9:47:32 AM |
| Analysis Name | D:\Data\SajalTuomas\230218-SKMTL-NB-2-P-83-HPLC_frac 3.d | | |
| Method | oligo.m | Operator | Bruker07 |
| Sample Name | 230218-SKMTL-NB-2-P-83-HPLC_frac 3 | Instrument | micrOTOF-Q |
| Comment | | | |

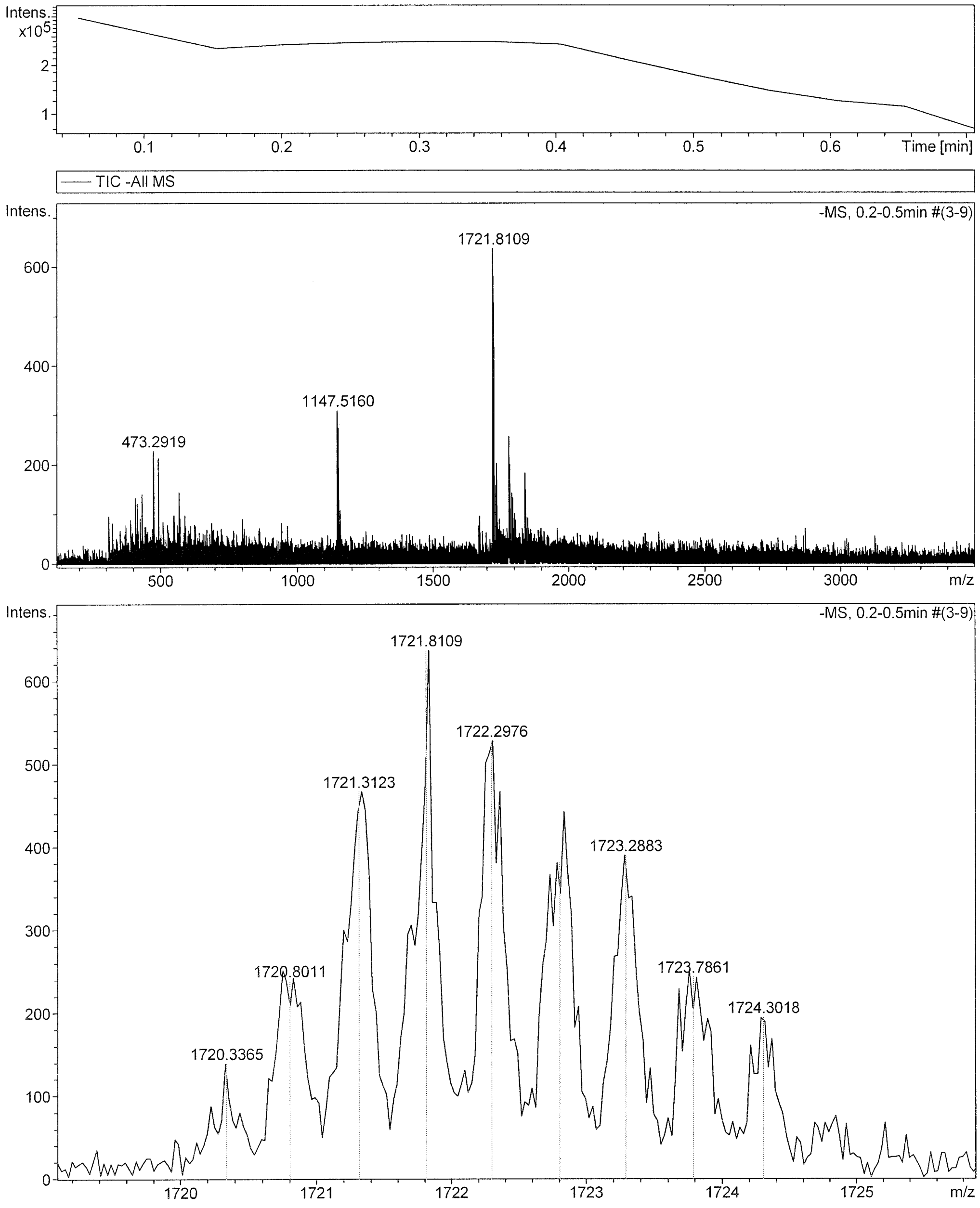


Bruker Compass DataAnalysis 4.0 printed: 2/23/2018 10:35:52 AM Page 1 of 1

**Figure S11.** Mass spectrum of oligonucleotide **ON1z-Pd**.

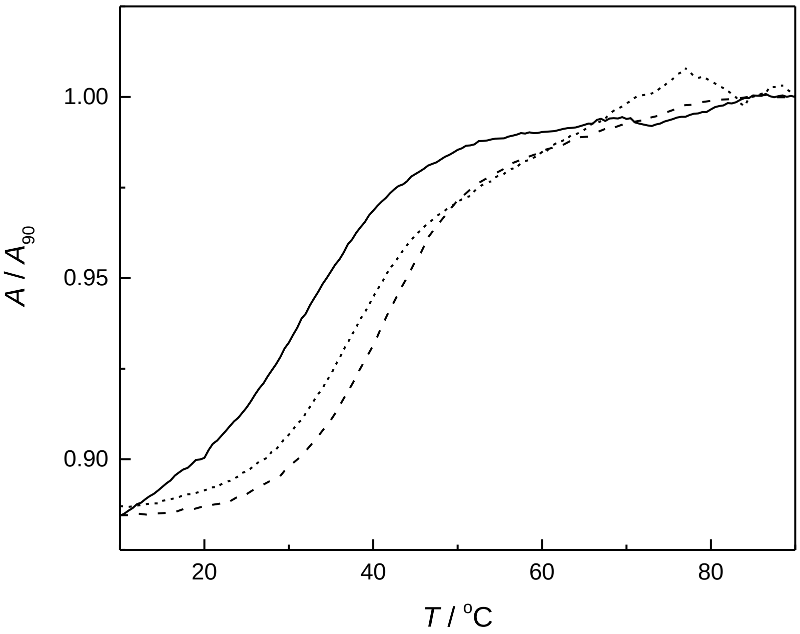


**Figure S12.** UV melting profiles for duplexes **ON1x•ON2a** (dotted and dashed line for faster and slower eluting isomer, respectively) and **ON1x-Hg•ON2a** (solid line); pH = 7.4 (20 mM cacodylate buffer); [oligonucleotides] = 1.0 μM; $I(NaClO_4)$ = 0.10 M.

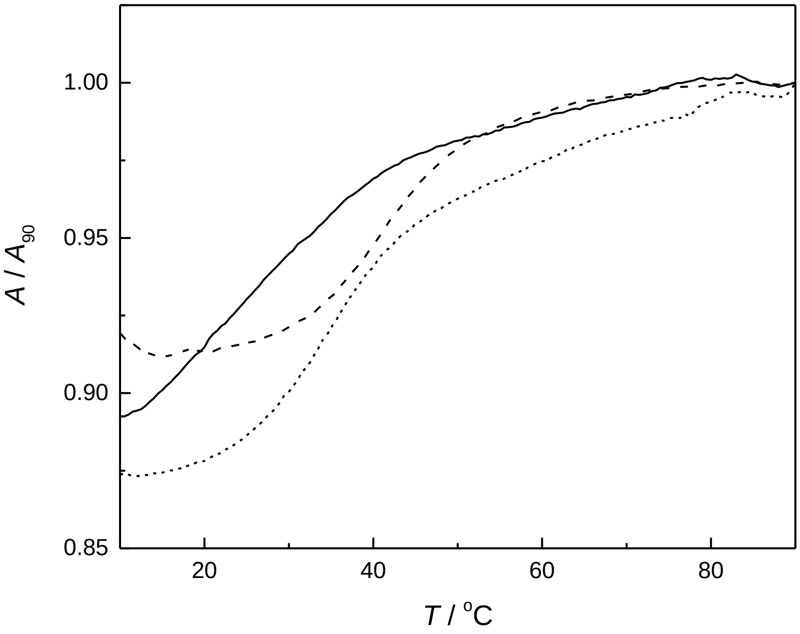


**Figure S13.** UV melting profiles for duplexes **ON1x•ON2c** (dotted and dashed line for faster and slower eluting isomer, respectively) and **ON1x-Hg•ON2c** (solid line); pH = 7.4 (20 mM cacodylate buffer); [oligonucleotides] = 1.0 μM; $I(NaClO_4)$ = 0.10 M.

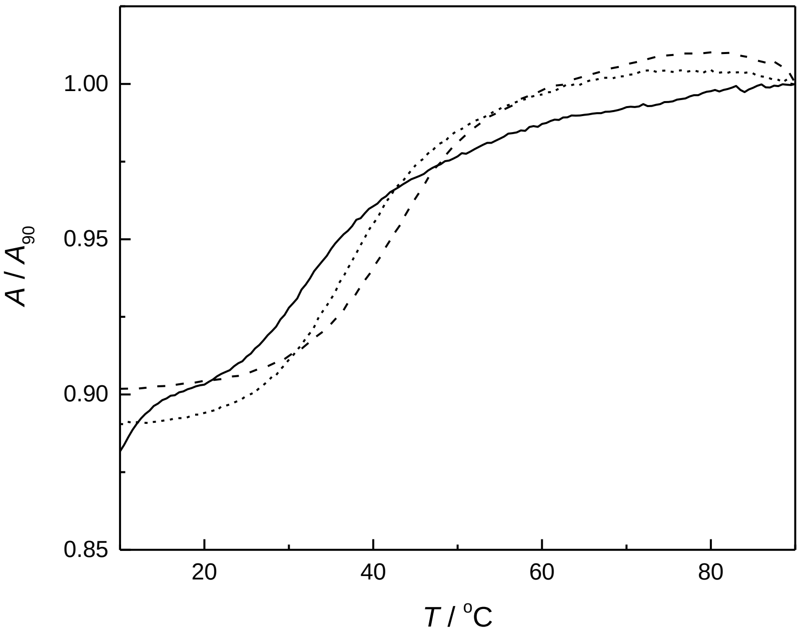


**Figure S14.** UV melting profiles for duplexes **ON1x•ON2g** (dotted and dashed line for faster and slower eluting isomer, respectively) and **ON1x-Hg•ON2g** (solid line); pH = 7.4 (20 mM cacodylate buffer); [oligonucleotides] = 1.0 μM; *I*($NaClO_4$) = 0.10 M.

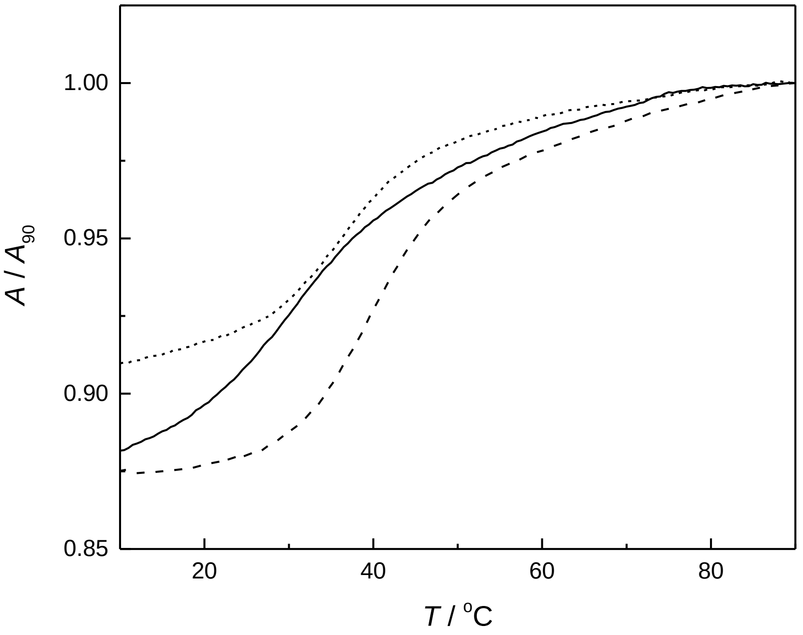


**Figure S15.** UV melting profiles for duplexes **ON1x•ON2t** (dotted and dashed line for faster and slower eluting isomer, respectively) and **ON1x-Hg•ON2t** (solid line); pH = 7.4 (20 mM cacodylate buffer); [oligonucleotides] = 1.0 μM; *I*($NaClO_4$) = 0.10 M.

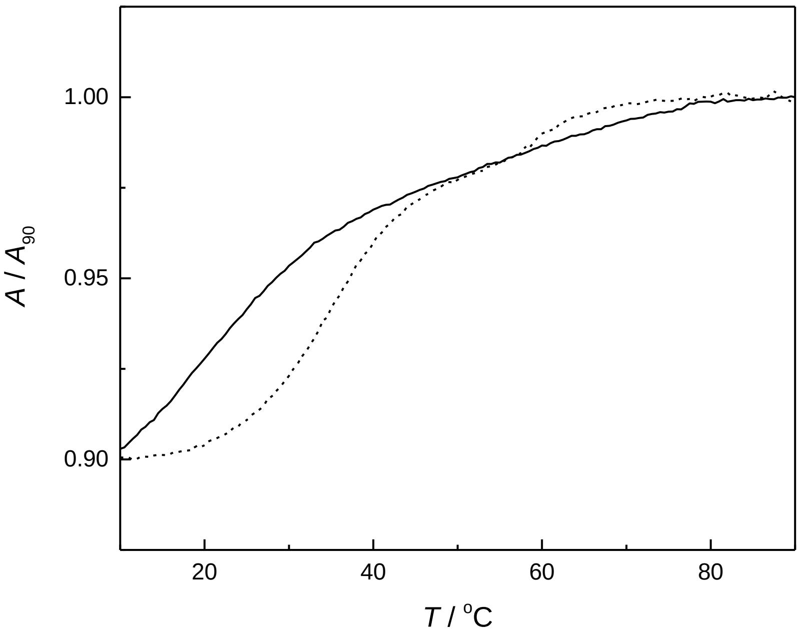


**Figure S16.** UV melting profiles for duplexes **ON1y•ON2a** (dotted line) and **ON1y-Pd•ON2a** (solid line); pH = 7.4 (20 mM cacodylate buffer); [oligonucleotides] = 1.0 μM; $I(NaClO_4)$ = 0.10 M.

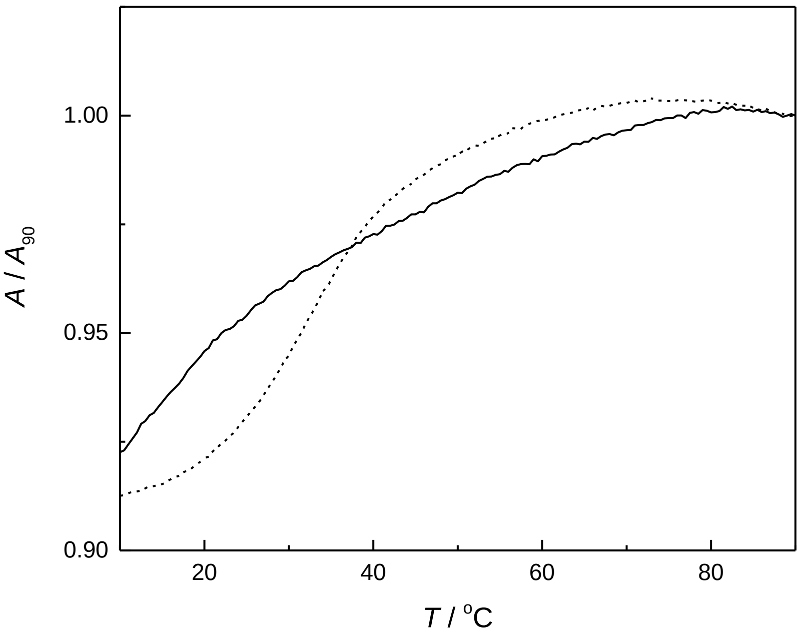


**Figure S17.** UV melting profiles for duplexes **ON1y•ON2c** (dotted line) and **ON1y-Pd•ON2c** (solid line); pH = 7.4 (20 mM cacodylate buffer); [oligonucleotides] = 1.0 μM; $I(NaClO_4)$ = 0.10 M.

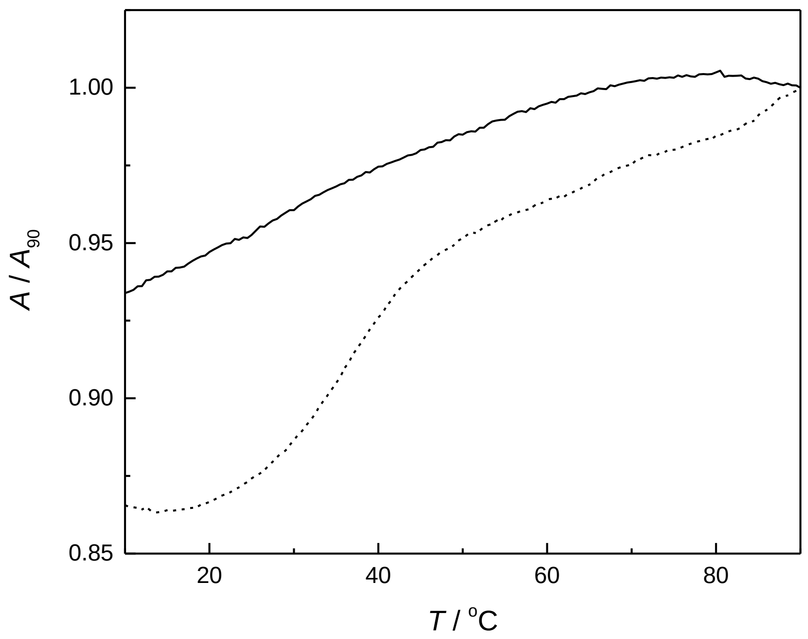


**Figure S18.** UV melting profiles for duplexes **ON1y•ON2g** (dotted line) and **ON1y-Pd•ON2g** (solid line); pH = 7.4 (20 mM cacodylate buffer); [oligonucleotides] = 1.0 μM; $I(NaClO_4)$ = 0.10 M.

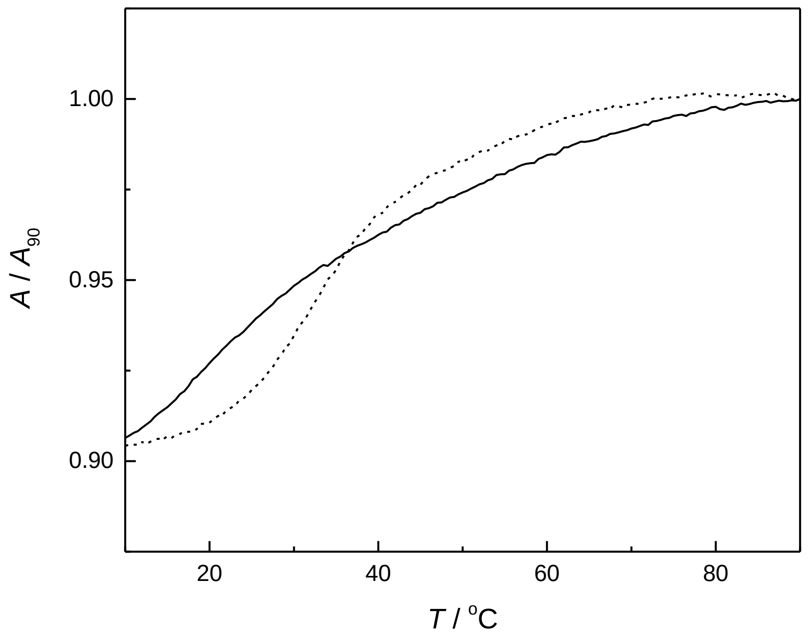


**Figure S19.** UV melting profiles for duplexes **ON1y•ON2t** (dotted line) and **ON1y-Pd•ON2t** (solid line); pH = 7.4 (20 mM cacodylate buffer); [oligonucleotides] = 1.0 μM; $I(NaClO_4)$ = 0.10 M.

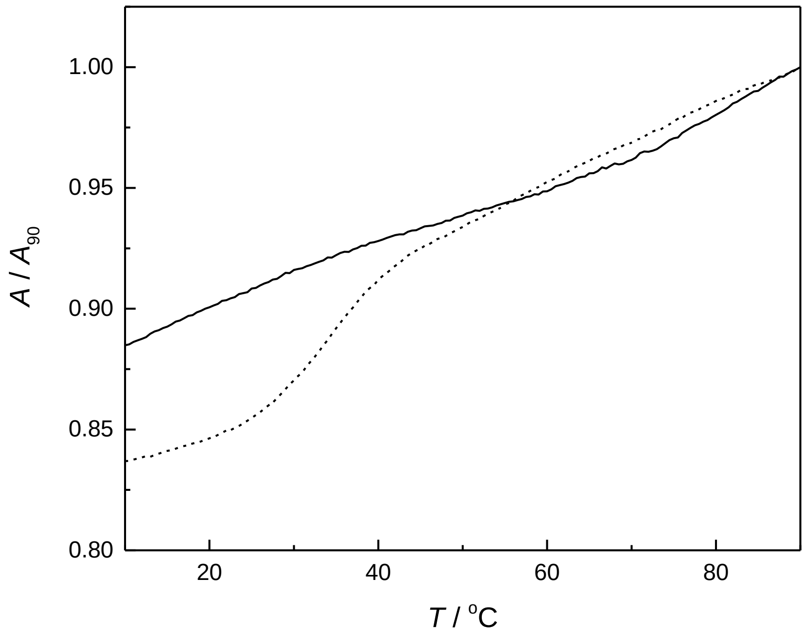


**Figure S20.** UV melting profiles for duplexes **ON1z•ON2a** (dotted line) and **ON1z-Pd•ON2a** (solid line); pH = 7.4 (20 mM cacodylate buffer); [oligonucleotides] = 1.0 μM; *I*($NaClO_4$) = 0.10 M.

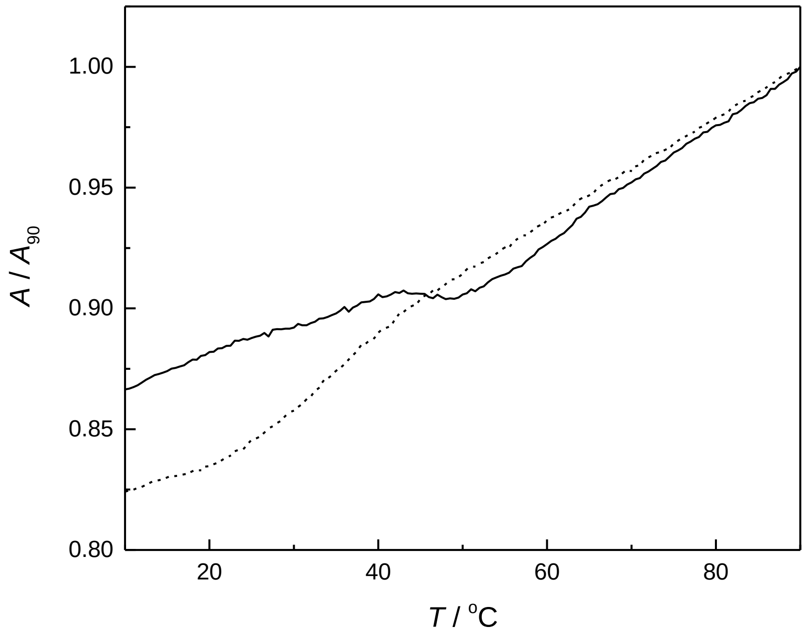


**Figure S21.** UV melting profiles for duplexes **ON1z•ON2c** (dotted line) and **ON1z-Pd•ON2c** (solid line); pH = 7.4 (20 mM cacodylate buffer); [oligonucleotides] = 1.0 μM; *I*($NaClO_4$) = 0.10 M.

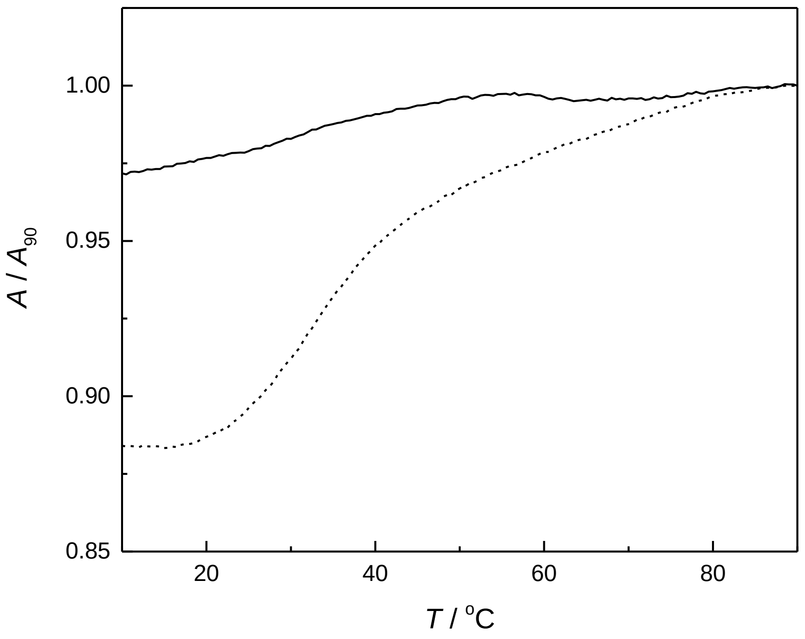


**Figure S22.** UV melting profiles for duplexes **ON1z•ON2g** (dotted line) and **ON1z-Pd•ON2g** (solid line); pH = 7.4 (20 mM cacodylate buffer); [oligonucleotides] = 1.0 μM; *I*($NaClO_4$) = 0.10 M.

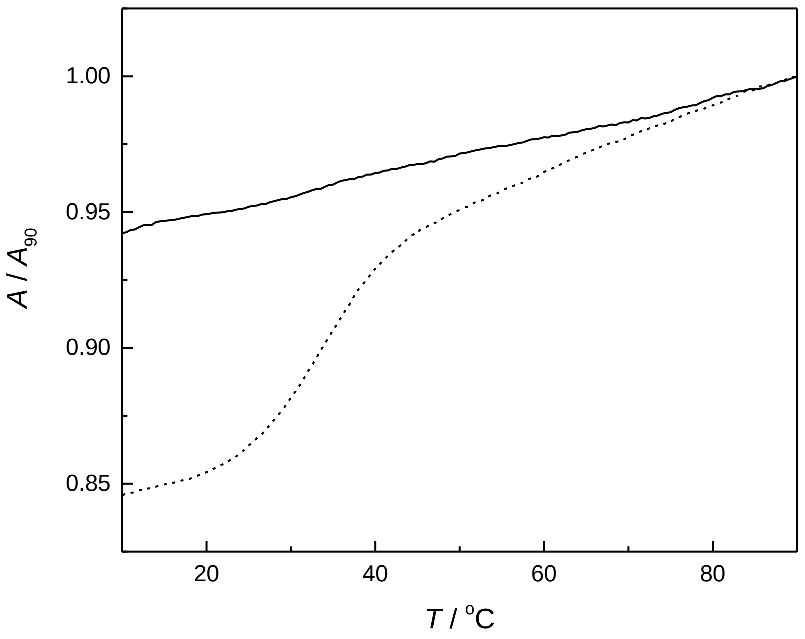


**Figure S23.** UV melting profiles for duplexes **ON1z•ON2t** (dotted line) and **ON1z-Pd•ON2t** (solid line); pH = 7.4 (20 mM cacodylate buffer); [oligonucleotides] = 1.0 μM; *I*($NaClO_4$) = 0.10 M.

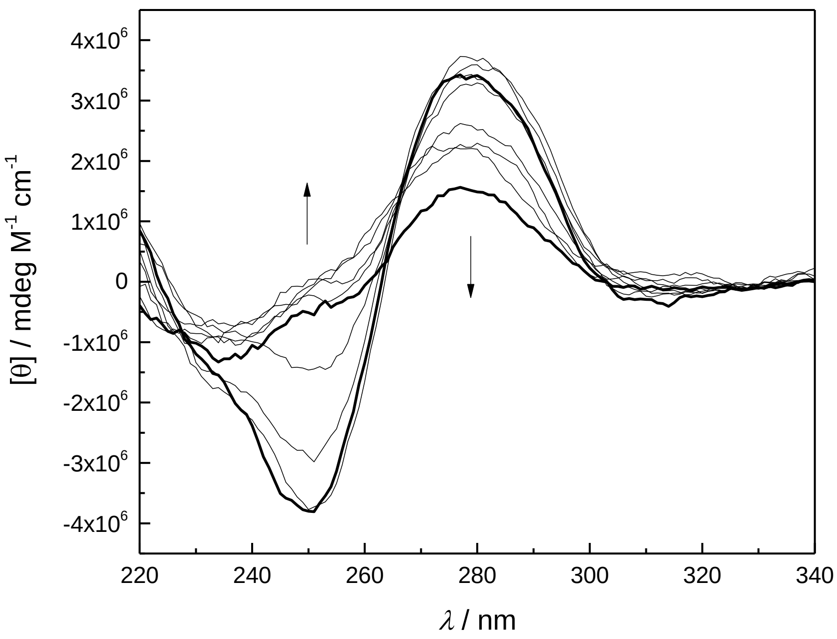


**Figure S24.** CD spectra of duplex **ON1x•ON2a** (faster eluting isomer of **ON1x**) recorded at 10 °C intervals between 10 and 90 °C; pH = 7.4 (20 mM cacodylate buffer); [oligonucleotides] = 1.0 μM; $I(NaClO_4)$ = 0.10 M. The spectra acquired at the extreme temperatures are indicated by thicker lines and the thermal loss of ellipticity by arrows.

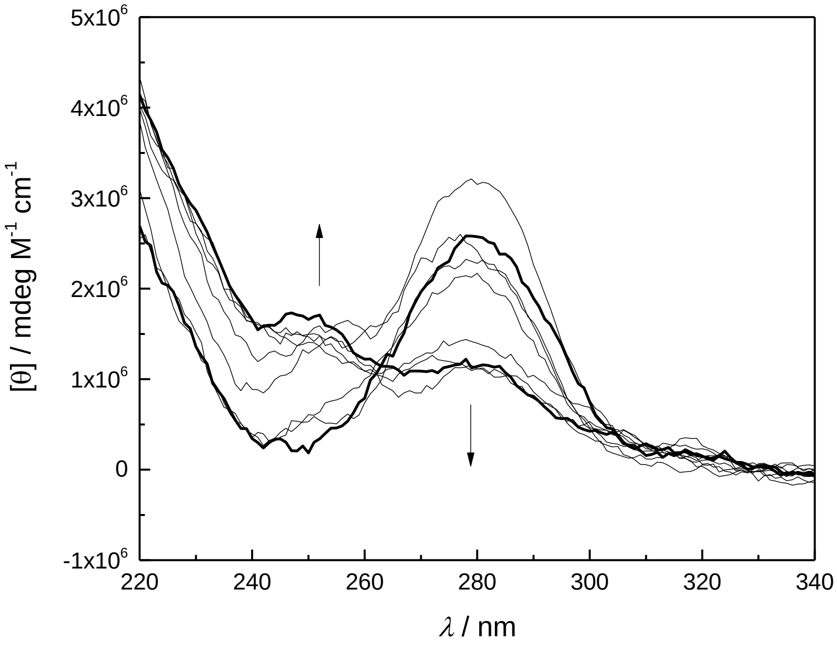


**Figure S25.** CD spectra of duplex **ON1x•ON2c** (faster eluting isomer of **ON1x**) recorded at 10 °C intervals between 10 and 90 °C; pH = 7.4 (20 mM cacodylate buffer); [oligonucleotides] = 1.0 μM; $I(NaClO_4)$ = 0.10 M. The spectra acquired at the extreme temperatures are indicated by thicker lines and the thermal loss of ellipticity by arrows.

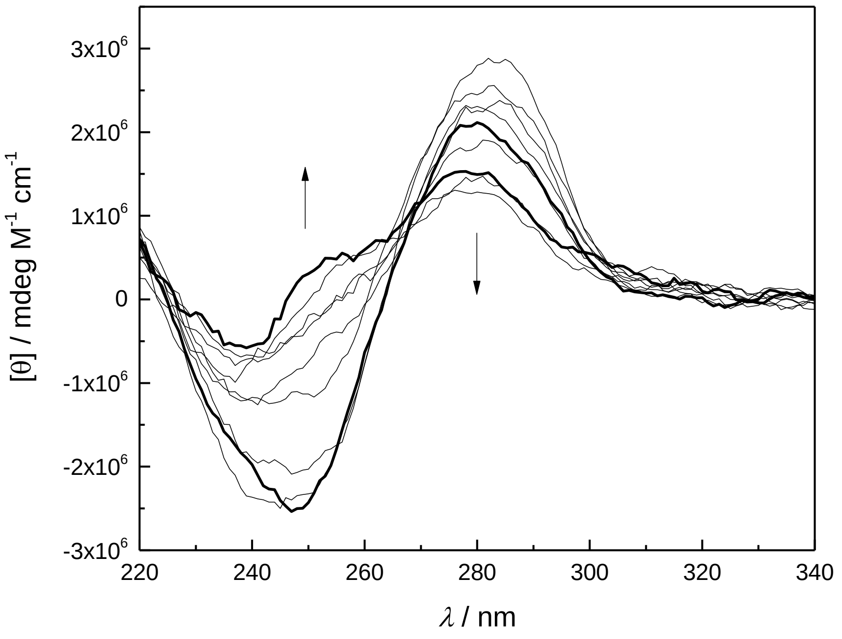


**Figure S26.** CD spectra of duplex **ON1x•ON2g** (faster eluting isomer of **ON1x**) recorded at 10 °C intervals between 10 and 90 °C; pH = 7.4 (20 mM cacodylate buffer); [oligonucleotides] = 1.0 μM; *I*($NaClO_4$) = 0.10 M. The spectra acquired at the extreme temperatures are indicated by thicker lines and the thermal loss of ellipticity by arrows.

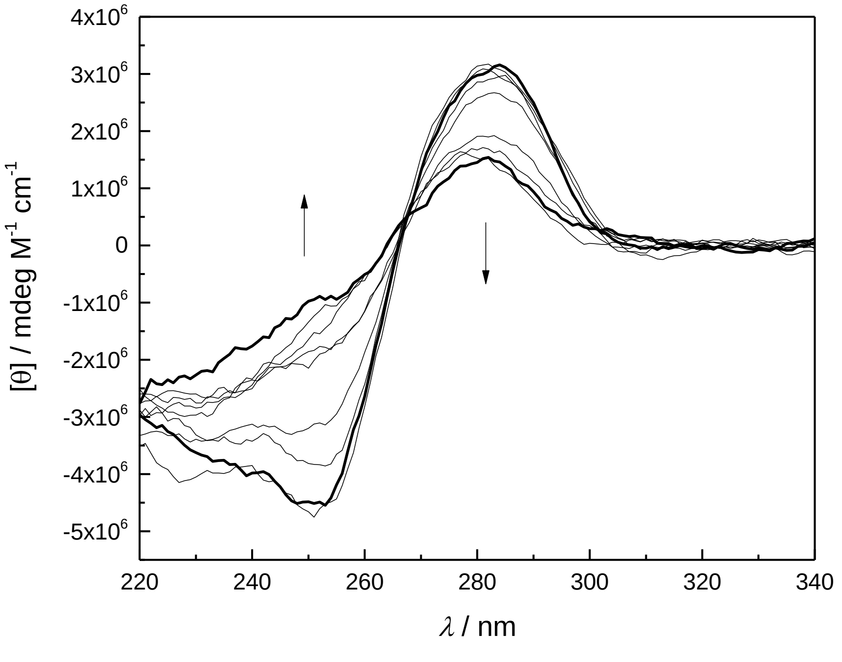


**Figure S27.** CD spectra of duplex **ON1x•ON2t** (faster eluting isomer of **ON1x**) recorded at 10 °C intervals between 10 and 90 °C; pH = 7.4 (20 mM cacodylate buffer); [oligonucleotides] = 1.0 μM; *I*($NaClO_4$) = 0.10 M. The spectra acquired at the extreme temperatures are indicated by thicker lines and the thermal loss of ellipticity by arrows.

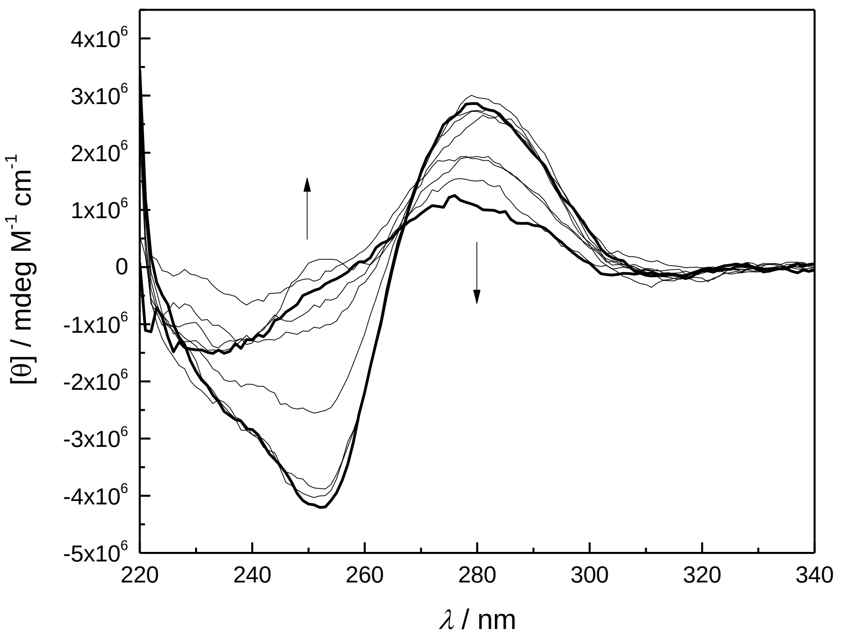


**Figure S28.** CD spectra of duplex **ON1x•ON2a** (slower eluting isomer of **ON1x**) recorded at 10 °C intervals between 10 and 90 °C; pH = 7.4 (20 mM cacodylate buffer); [oligonucleotides] = 1.0 μM; $I(NaClO_4)$ = 0.10 M. The spectra acquired at the extreme temperatures are indicated by thicker lines and the thermal loss of ellipticity by arrows.

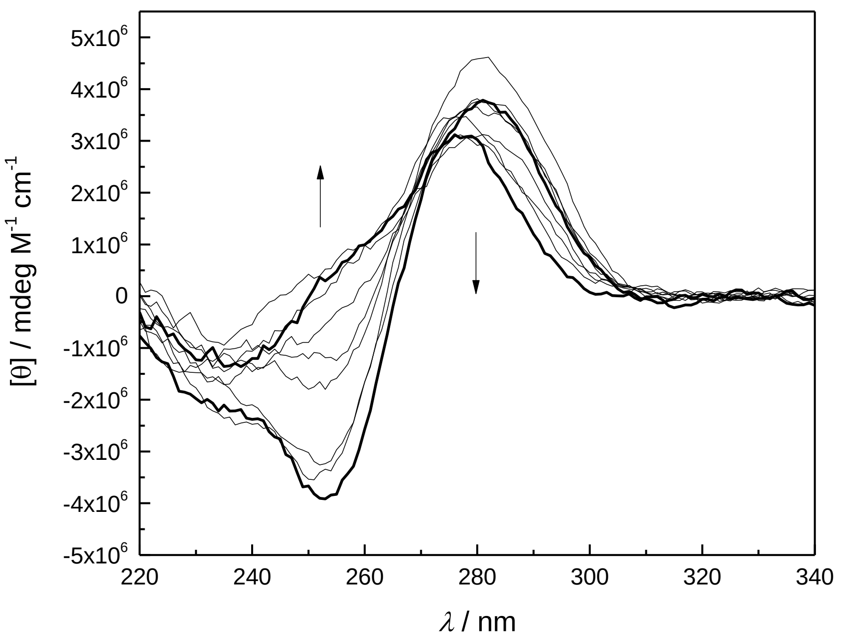


**Figure S29.** CD spectra of duplex **ON1x•ON2c** (slower eluting isomer of **ON1x**) recorded at 10 °C intervals between 10 and 90 °C; pH = 7.4 (20 mM cacodylate buffer); [oligonucleotides] = 1.0 μM; $I(NaClO_4)$ = 0.10 M. The spectra acquired at the extreme temperatures are indicated by thicker lines and the thermal loss of ellipticity by arrows.

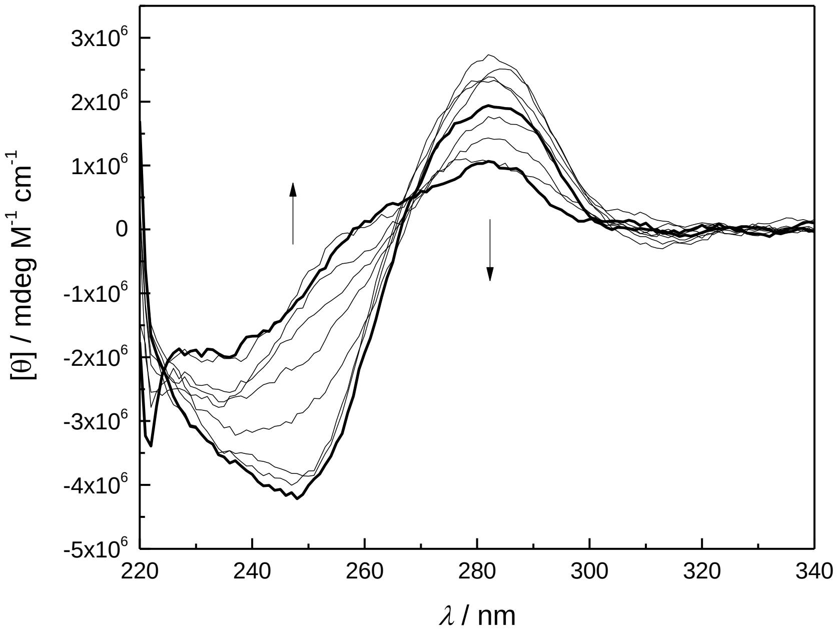


**Figure S30.** CD spectra of duplex **ON1x•ON2g** (slower eluting isomer of **ON1x**) recorded at 10 °C intervals between 10 and 90 °C; pH = 7.4 (20 mM cacodylate buffer); [oligonucleotides] = 1.0 μM; *I*($NaClO_4$) = 0.10 M. The spectra acquired at the extreme temperatures are indicated by thicker lines and the thermal loss of ellipticity by arrows.

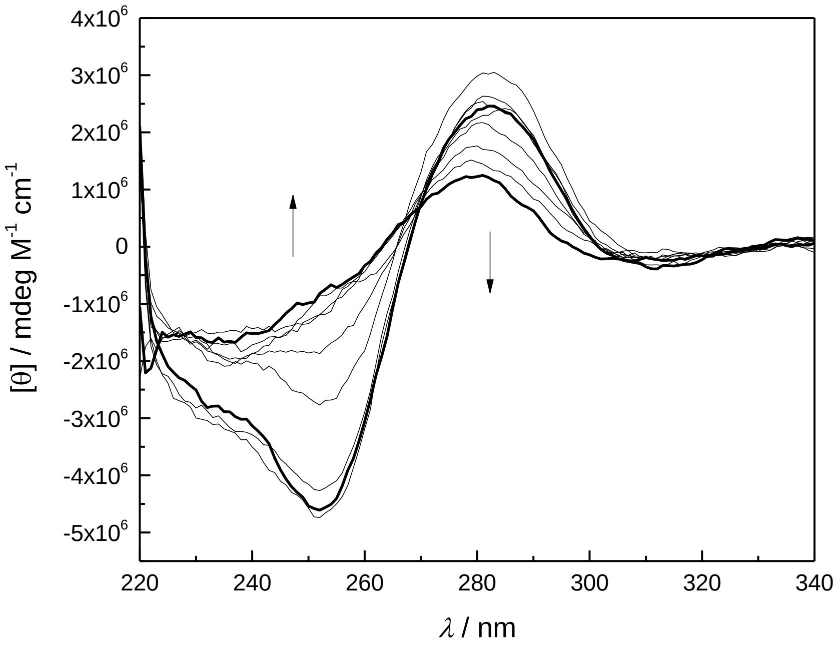


**Figure S31.** CD spectra of duplex **ON1x•ON2t** (slower eluting isomer of **ON1x**) recorded at 10 °C intervals between 10 and 90 °C; pH = 7.4 (20 mM cacodylate buffer); [oligonucleotides] = 1.0 μM; *I*($NaClO_4$) = 0.10 M. The spectra acquired at the extreme temperatures are indicated by thicker lines and the thermal loss of ellipticity by arrows.

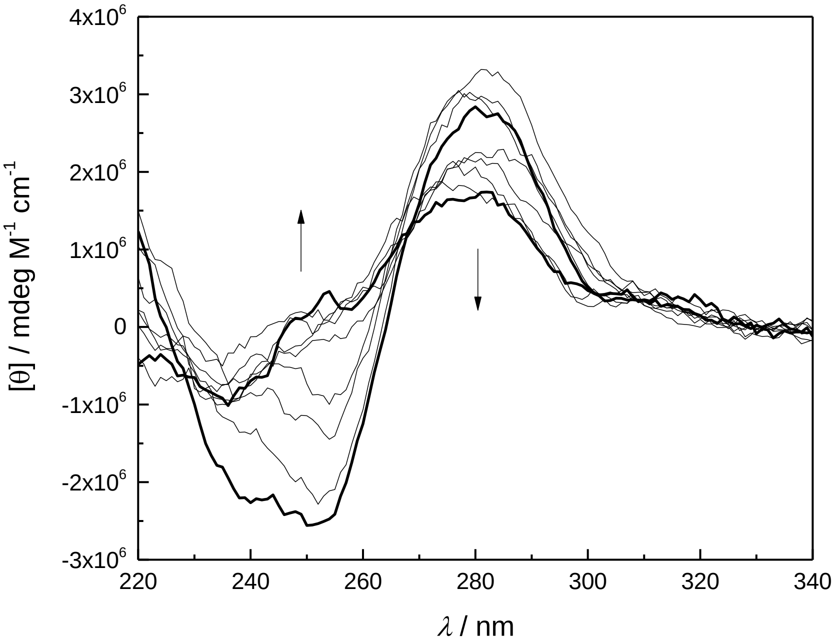


**Figure S32.** CD spectra of duplex **ON1x-Hg•ON2a**, recorded at 10 °C intervals between 10 and 90 °C; pH = 7.4 (20 mM cacodylate buffer); [oligonucleotides] = 1.0 μM; $I(NaClO_4)$ = 0.10 M. The spectra acquired at the extreme temperatures are indicated by thicker lines and the thermal loss of ellipticity by arrows.

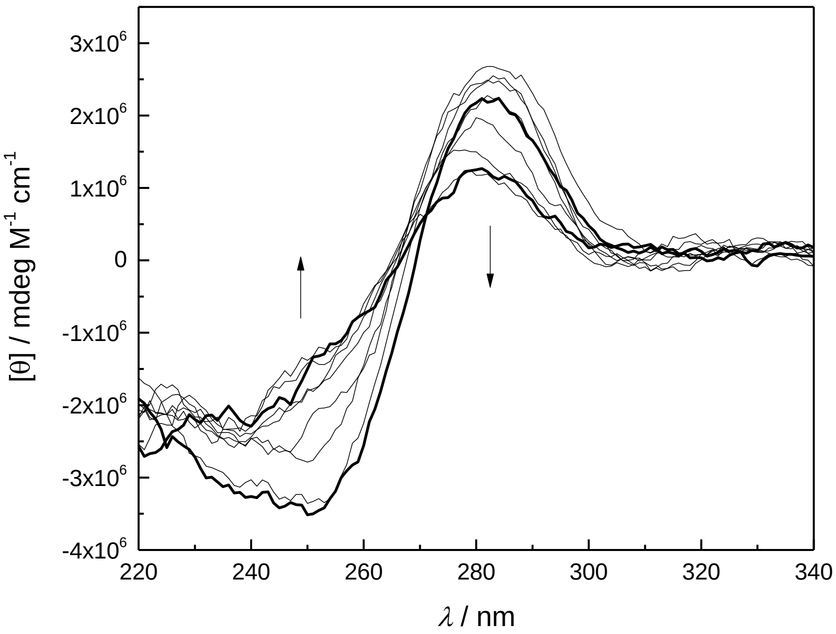


**Figure S33.** CD spectra of duplex **ON1x-Hg•ON2c**, recorded at 10 °C intervals between 10 and 90 °C; pH = 7.4 (20 mM cacodylate buffer); [oligonucleotides] = 1.0 μM; $I(NaClO_4)$ = 0.10 M. The spectra acquired at the extreme temperatures are indicated by thicker lines and the thermal loss of ellipticity by arrows.

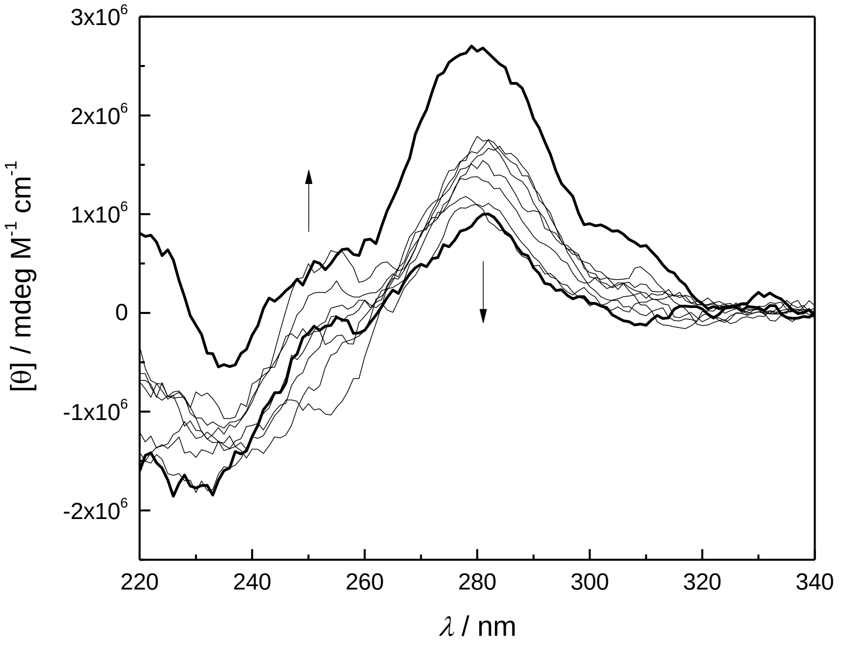


**Figure S34.** CD spectra of duplex **ON1x-Hg•ON2g**, recorded at 10 °C intervals between 10 and 90 °C; pH = 7.4 (20 mM cacodylate buffer); [oligonucleotides] = 1.0 μM; $I(NaClO_4)$ = 0.10 M. The spectra acquired at the extreme temperatures are indicated by thicker lines and the thermal loss of ellipticity by arrows.

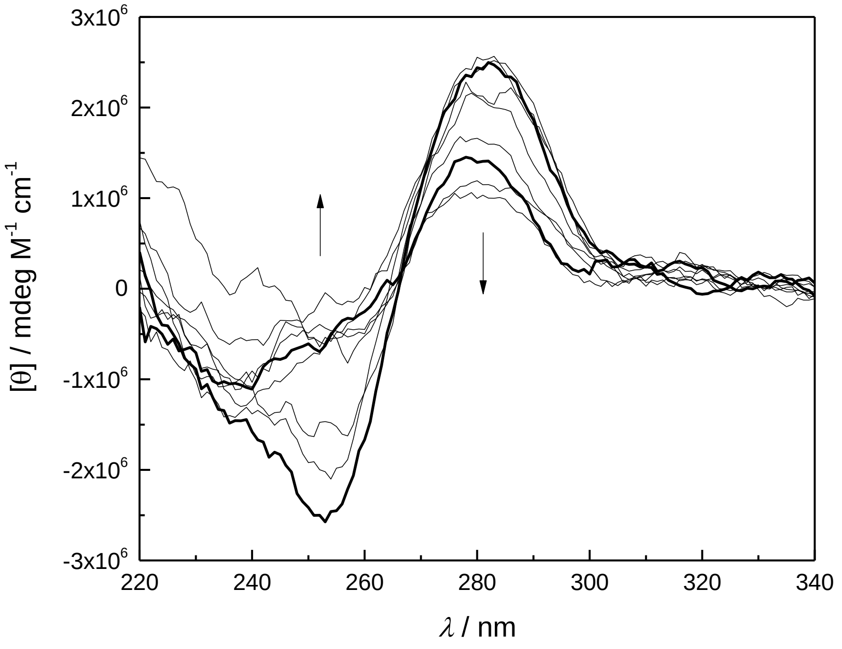


**Figure S35.** CD spectra of duplex **ON1x-Hg•ON2t**, recorded at 10 °C intervals between 10 and 90 °C; pH = 7.4 (20 mM cacodylate buffer); [oligonucleotides] = 1.0 μM; $I(NaClO_4)$ = 0.10 M. The spectra acquired at the extreme temperatures are indicated by thicker lines and the thermal loss of ellipticity by arrows.

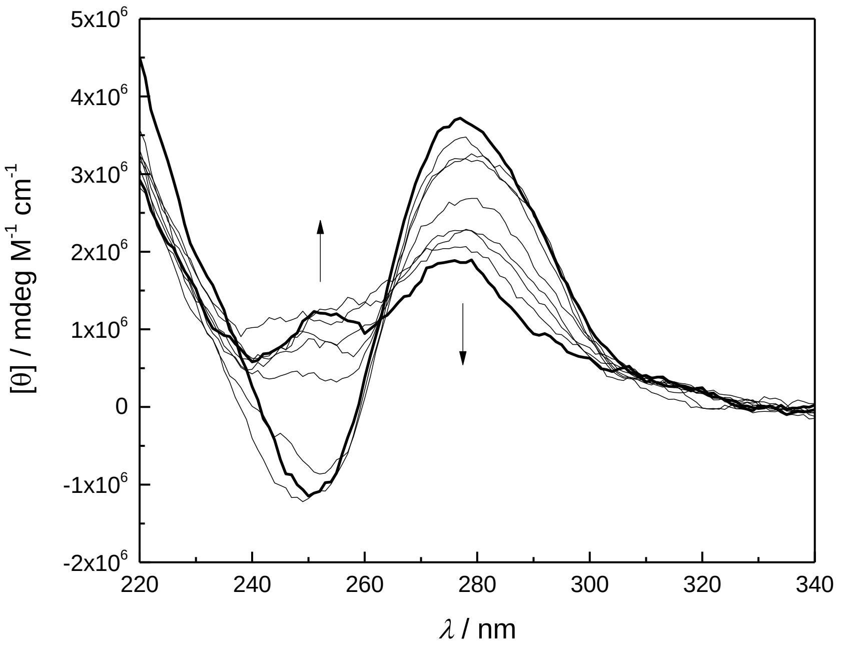


**Figure S36.** CD spectra of duplex **ON1y•ON2a**, recorded at 10 °C intervals between 10 and 90 °C; pH = 7.4 (20 mM cacodylate buffer); [oligonucleotides] = 1.0 μM; $I(NaClO_4)$ = 0.10 M. The spectra acquired at the extreme temperatures are indicated by thicker lines and the thermal loss of ellipticity by arrows.

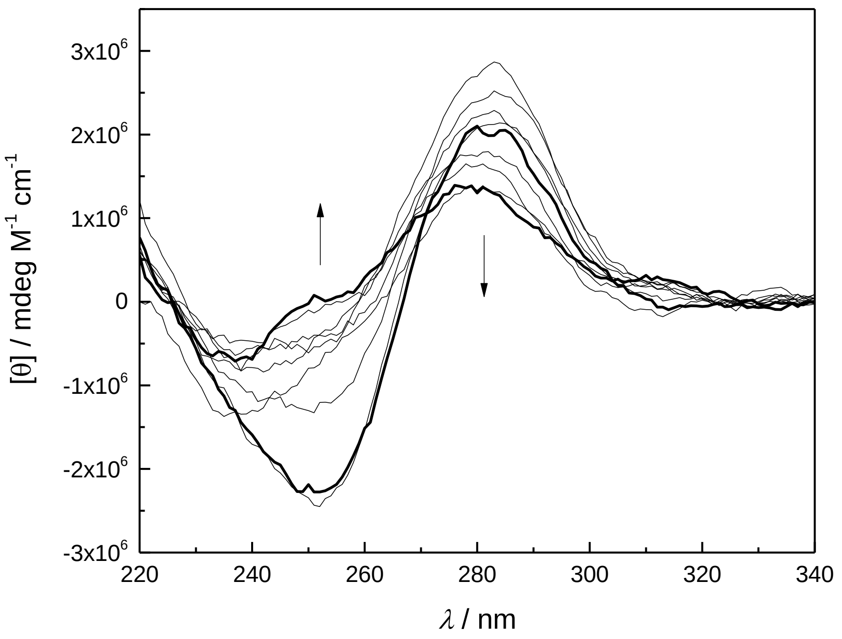


**Figure S37.** CD spectra of duplex **ON1y•ON2c**, recorded at 10 °C intervals between 10 and 90 °C; pH = 7.4 (20 mM cacodylate buffer); [oligonucleotides] = 1.0 μM; $I(NaClO_4)$ = 0.10 M. The spectra acquired at the extreme temperatures are indicated by thicker lines and the thermal loss of ellipticity by arrows.

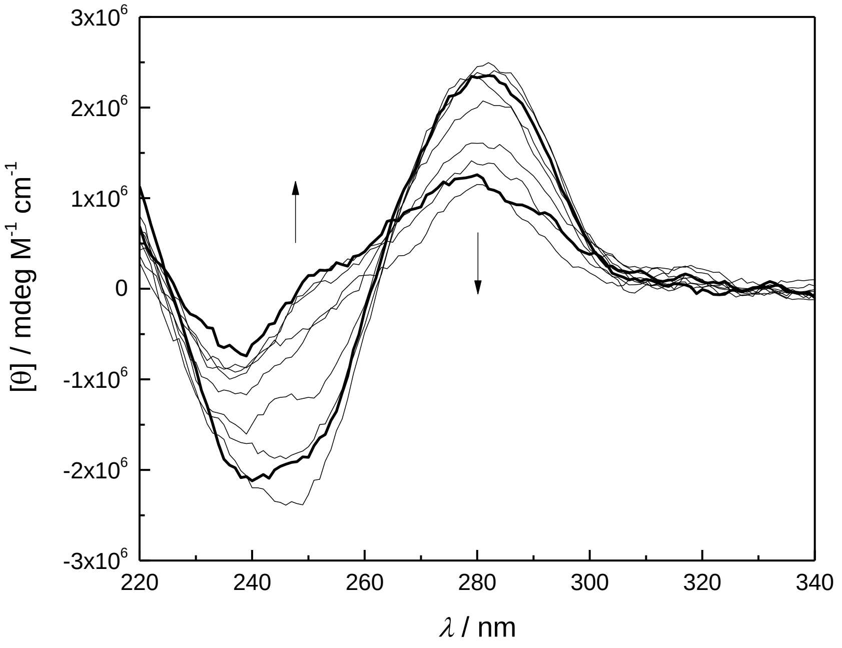


**Figure S38.** CD spectra of duplex **ON1y•ON2g**, recorded at 10 °C intervals between 10 and 90 °C; pH = 7.4 (20 mM cacodylate buffer); [oligonucleotides] = 1.0 μM; *I*($NaClO_4$) = 0.10 M. The spectra acquired at the extreme temperatures are indicated by thicker lines and the thermal loss of ellipticity by arrows.

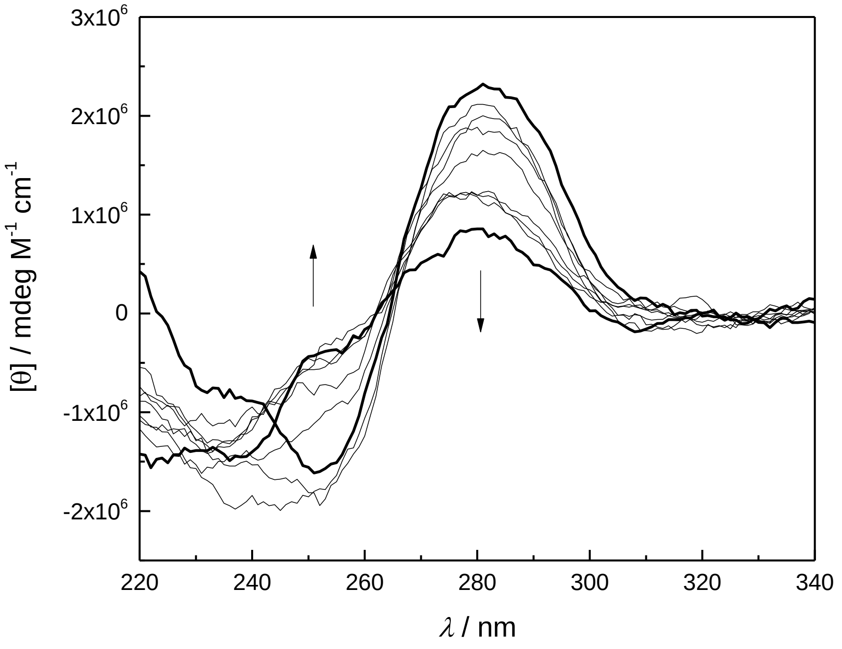


**Figure S39.** CD spectra of duplex **ON1y•ON2t**, recorded at 10 °C intervals between 10 and 90 °C; pH = 7.4 (20 mM cacodylate buffer); [oligonucleotides] = 1.0 μM; *I*($NaClO_4$) = 0.10 M. The spectra acquired at the extreme temperatures are indicated by thicker lines and the thermal loss of ellipticity by arrows.

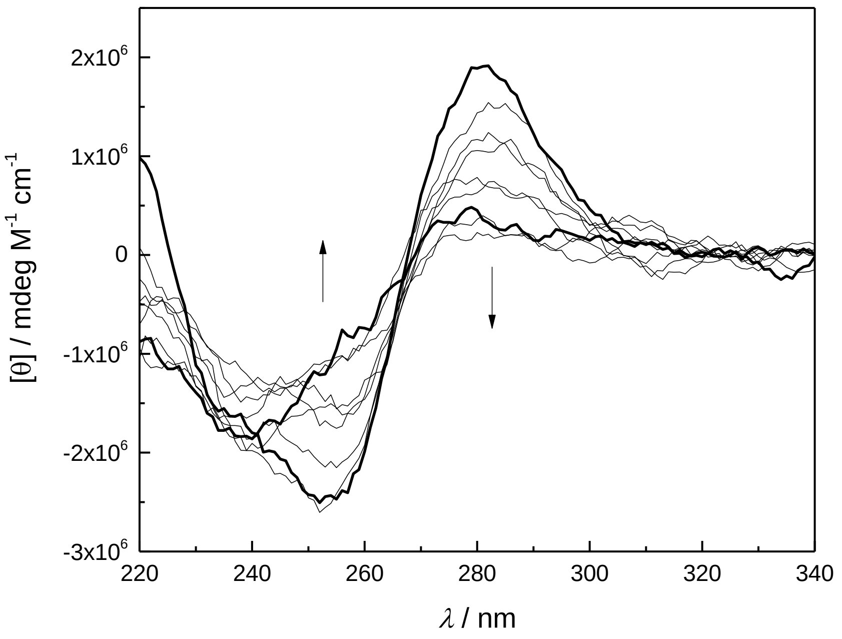


**Figure S40.** CD spectra of duplex **ON1y-Pd•ON2a**, recorded at 10 °C intervals between 10 and 90 °C; pH = 7.4 (20 mM cacodylate buffer); [oligonucleotides] = 1.0 μM; $I(NaClO_4)$ = 0.10 M. The spectra acquired at the extreme temperatures are indicated by thicker lines and the thermal loss of ellipticity by arrows.

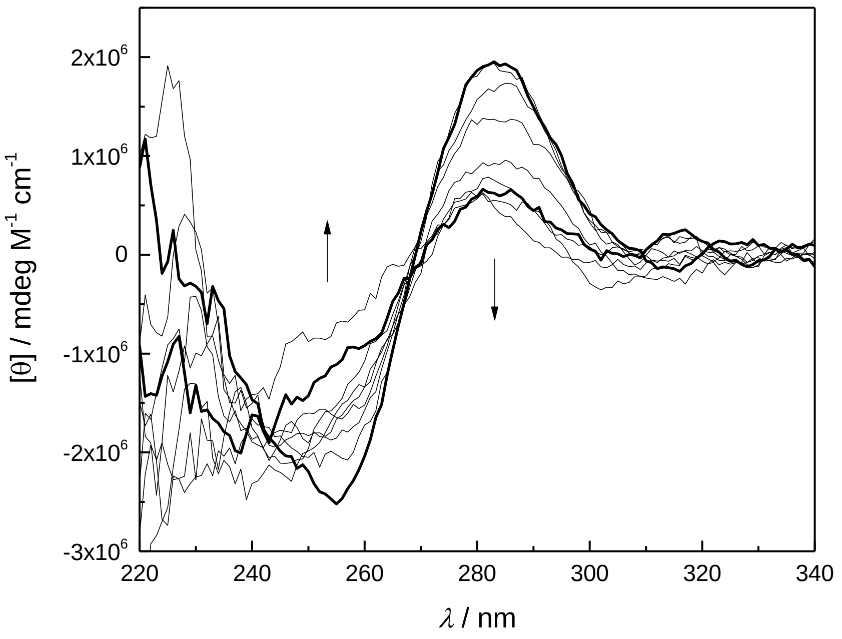


**Figure S41.** CD spectra of duplex **ON1y-Pd•ON2c**, recorded at 10 °C intervals between 10 and 90 °C; pH = 7.4 (20 mM cacodylate buffer); [oligonucleotides] = 1.0 μM; $I(NaClO_4)$ = 0.10 M. The spectra acquired at the extreme temperatures are indicated by thicker lines and the thermal loss of ellipticity by arrows.

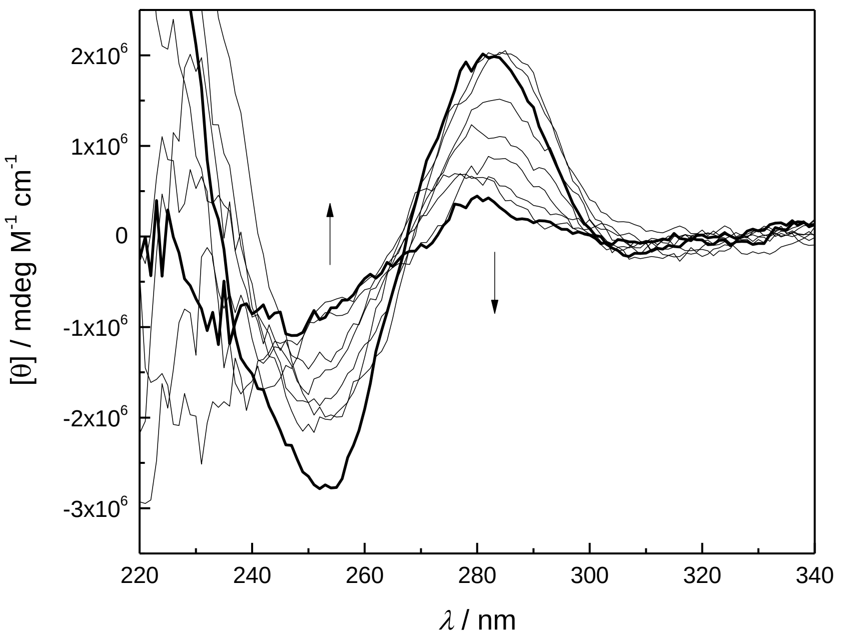


**Figure S42.** CD spectra of duplex **ON1y-Pd•ON2g**, recorded at 10 °C intervals between 10 and 90 °C; pH = 7.4 (20 mM cacodylate buffer); [oligonucleotides] = 1.0 μM; *I*($NaClO_4$) = 0.10 M. The spectra acquired at the extreme temperatures are indicated by thicker lines and the thermal loss of ellipticity by arrows.

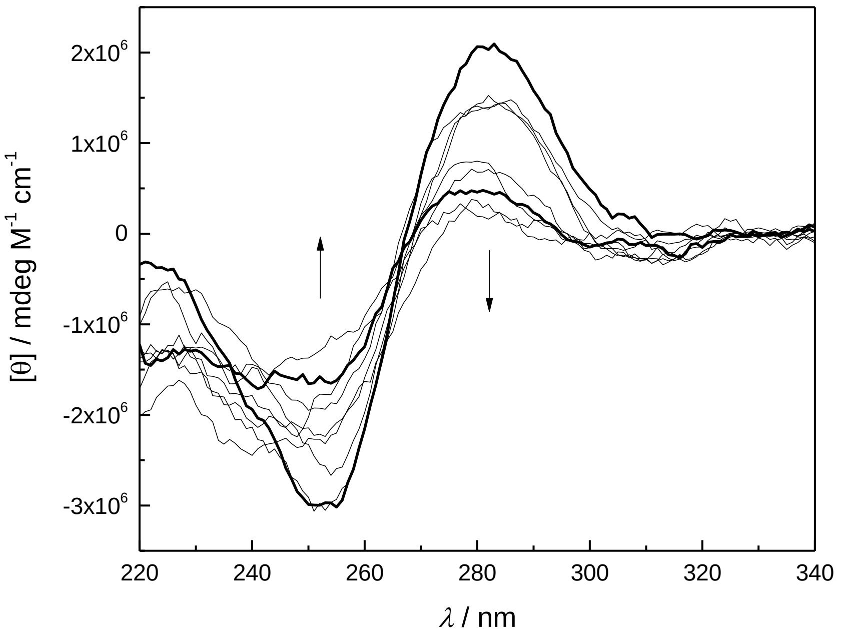


**Figure S43.** CD spectra of duplex **ON1y-Pd•ON2t**, recorded at 10 °C intervals between 10 and 90 °C; pH = 7.4 (20 mM cacodylate buffer); [oligonucleotides] = 1.0 μM; *I*($NaClO_4$) = 0.10 M. The spectra acquired at the extreme temperatures are indicated by thicker lines and the thermal loss of ellipticity by arrows.

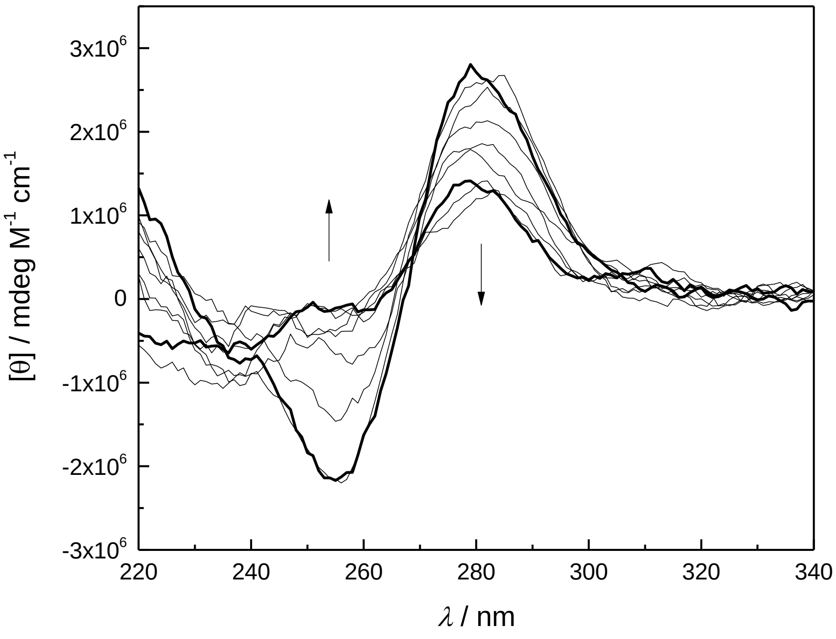


**Figure S44.** CD spectra of duplex **ON1z•ON2a**, recorded at 10 °C intervals between 10 and 90 °C; pH = 7.4 (20 mM cacodylate buffer); [oligonucleotides] = 1.0 μM; *I*($NaClO_4$) = 0.10 M. The spectra acquired at the extreme temperatures are indicated by thicker lines and the thermal loss of ellipticity by arrows.

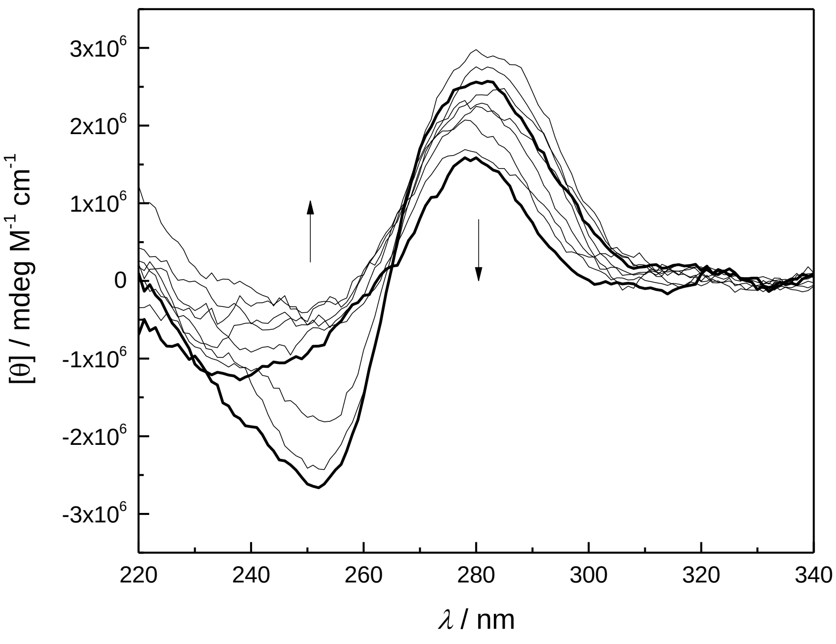


**Figure S45.** CD spectra of duplex **ON1z•ON2c**, recorded at 10 °C intervals between 10 and 90 °C; pH = 7.4 (20 mM cacodylate buffer); [oligonucleotides] = 1.0 μM; *I*($NaClO_4$) = 0.10 M. The spectra acquired at the extreme temperatures are indicated by thicker lines and the thermal loss of ellipticity by arrows.

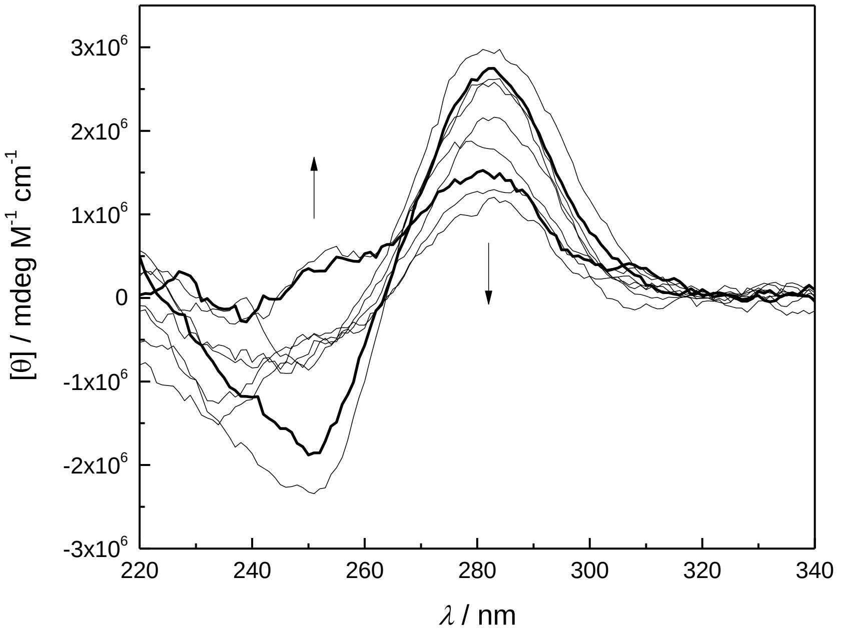


**Figure S46.** CD spectra of duplex **ON1z•ON2g**, recorded at 10 °C intervals between 10 and 90 °C; pH = 7.4 (20 mM cacodylate buffer); [oligonucleotides] = 1.0 μM; $I(NaClO_4)$ = 0.10 M. The spectra acquired at the extreme temperatures are indicated by thicker lines and the thermal loss of ellipticity by arrows.

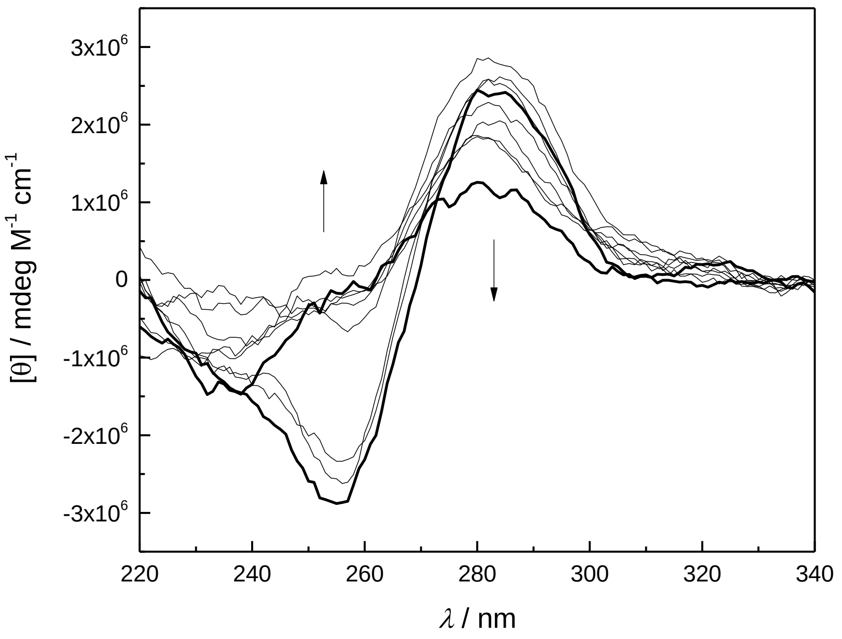


**Figure S47.** CD spectra of duplex **ON1z•ON2t**, recorded at 10 °C intervals between 10 and 90 °C; pH = 7.4 (20 mM cacodylate buffer); [oligonucleotides] = 1.0 μM; $I(NaClO_4)$ = 0.10 M. The spectra acquired at the extreme temperatures are indicated by thicker lines and the thermal loss of ellipticity by arrows.

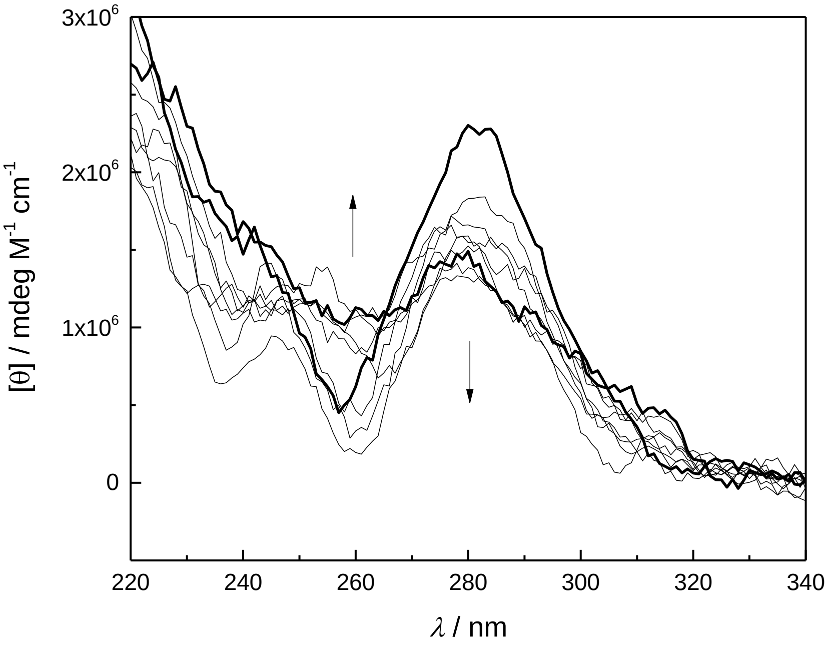


**Figure S48.** CD spectra of duplex **ON1z-Pd•ON2a**, recorded at 10 °C intervals between 10 and 90 °C; pH = 7.4 (20 mM cacodylate buffer); [oligonucleotides] = 1.0 μM; $I(NaClO_4)$ = 0.10 M. The spectra acquired at the extreme temperatures are indicated by thicker lines and the thermal loss of ellipticity by arrows.

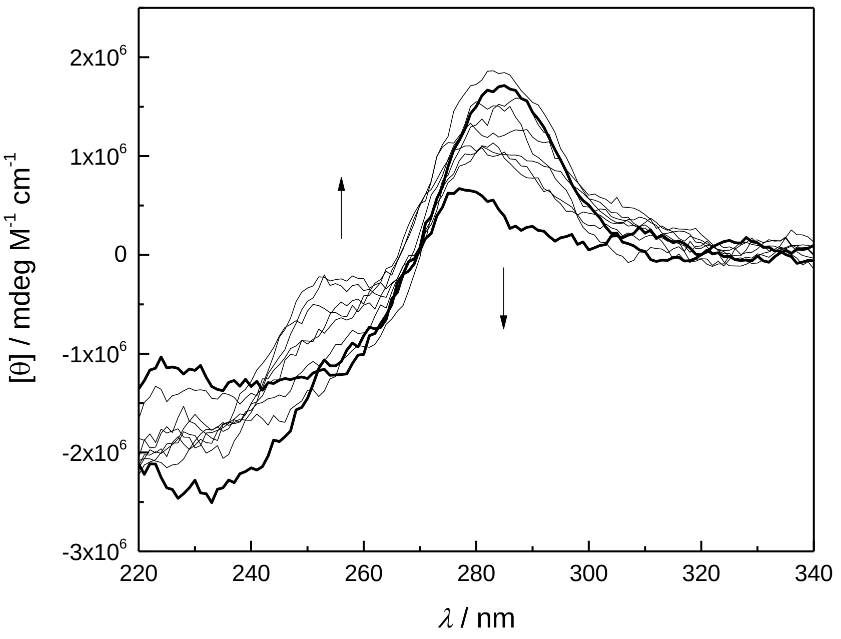


**Figure S49.** CD spectra of duplex **ON1z-Pd•ON2c**, recorded at 10 °C intervals between 10 and 90 °C; pH = 7.4 (20 mM cacodylate buffer); [oligonucleotides] = 1.0 μM; $I(NaClO_4)$ = 0.10 M. The spectra acquired at the extreme temperatures are indicated by thicker lines and the thermal loss of ellipticity by arrows.

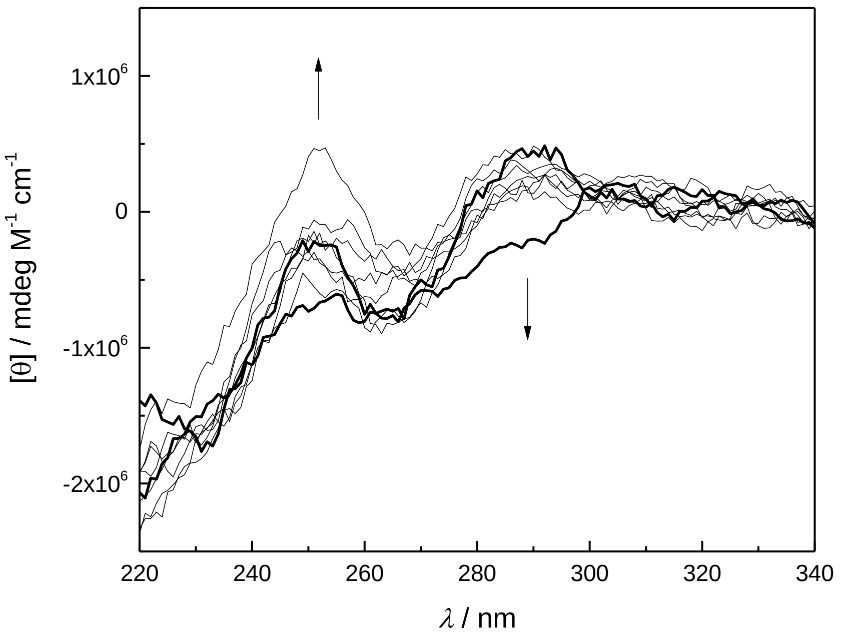


**Figure S50.** CD spectra of duplex **ON1z-Pd•ON2g**, recorded at 10 °C intervals between 10 and 90 °C; pH = 7.4 (20 mM cacodylate buffer); [oligonucleotides] = 1.0 μM; *I*($NaClO_4$) = 0.10 M. The spectra acquired at the extreme temperatures are indicated by thicker lines and the thermal loss of ellipticity by arrows.

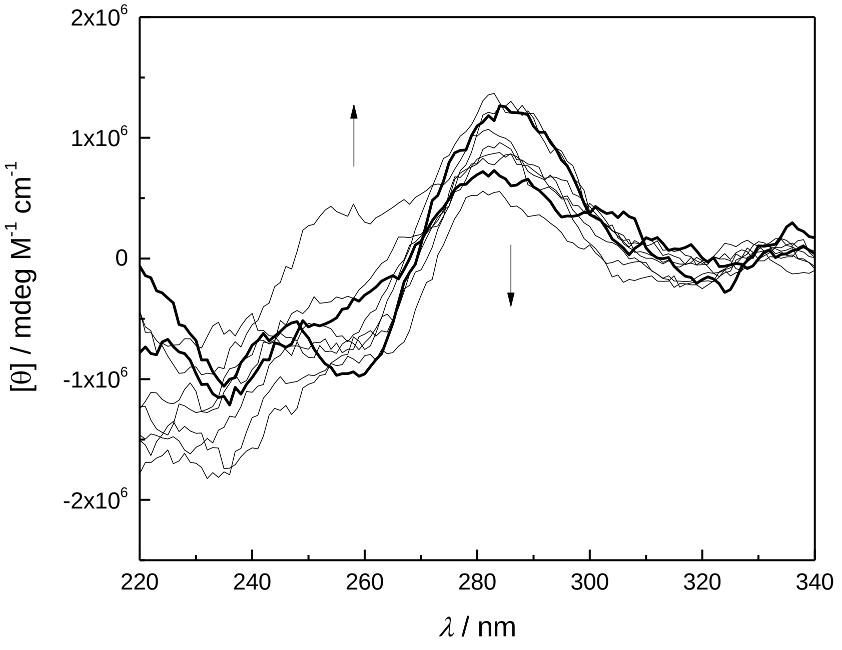


**Figure S51.** CD spectra of duplex **ON1z-Pd•ON2t**, recorded at 10 °C intervals between 10 and 90 °C; pH = 7.4 (20 mM cacodylate buffer); [oligonucleotides] = 1.0 μM; *I*($NaClO_4$) = 0.10 M. The spectra acquired at the extreme temperatures are indicated by thicker lines and the thermal loss of ellipticity by arrows.
